# Characterization of rumen microbiota in lactating Holstein cows fed molasses versus corn grain at two levels of rumen-degradable protein

**DOI:** 10.3389/frmbi.2023.1204988

**Published:** 2023-08-15

**Authors:** E. Guduk, M. B. Hall, G. I. Zanton, A. J. Steinberger, P. J. Weimer, G. Suen, K. A. Weigel

**Affiliations:** ^1^ Department of Animal and Dairy Sciences, University of Wisconsin-Madison, Madison, WI, United States; ^2^ U. S. Dairy Forage Research Center, USDA-Agricultural Research Service, Madison, WI, United States; ^3^ Department of Bacteriology, University of Wisconsin-Madison, Madison, WI, United States

**Keywords:** rumen, sugar, starch, degradable protein, microbiome, ruminant, dairy cattle

## Abstract

We evaluated the influence of diets differing in non-fiber carbohydrates and rumen-degradable protein (RDP) levels on changes in the ruminal bacterial populations in lactating Holstein cows. In all, 12 ruminally cannulated cows were assigned to diets with high or low RDP levels. Within each RDP level, molasses was substituted for corn grain at a concentration of 0%, 5.25%, or 10.5% of diet dry matter in a replicated 3 × 3 Latin square design with 28-day periods. Liquid and solid rumen digesta fractions collected at the end of each period underwent 16S rRNA gene sequencing to identify operational taxonomic units and were analyzed for short-chain fatty acids. Protein degradability affected 6 bacterial genera, whereas carbohydrate alteration impacted 13 genera (*p* < 0.05). Of the 30 genera with the highest relative abundance, 26 differed by digesta fraction (*p* < 0.05), with Bacteroidetes genera showing a greater abundance in solids and Firmicutes genera demonstrating a greater prevalence in liquids. Regarding relative abundances, with increasing molasses, *Succiniclasticum* decreased in liquid (*p* < 0.05), and *CF231*, *YRC22*, *Clostridium*, *Desulfovibrio*, *BF311*, and *Oscillospira* increased in solids (*p* < 0.05). In contrast, at higher RDP levels, *Succiniclasticum* increased while *YRC22* and *Pseudobutyrivibrio* decreased in solids (*p* < 0.05). Genera with abundances found to be correlated with fermentation products in the liquid included *Shuttleworthia*, *Treponema*, *Lachnospira*, and *Schwartzia*, which typically have lower relative abundances, showing strong positive correlations with molar proportions (mol%) of propionate, butyrate, and valerate (*p* < 0.05), and negative correlations with pH and acetate mol% (*p* < 0.05). *Fibrobacter* was positively correlated with lactate mol% (*p* < 0.05). Butyrate mol% exhibited a quadratic increase as molasses increased (*p* = 0.017), and lactate mol% rose with increased RDP levels (*p* = 0.042). No treatment effects were detected for pH propionate and valerate mol%; however, we observed a tendency (*p* = 0.075) for a quadratic effect of molasses treatment on the mol% of acetate. These findings substantiate the pivotal role of diet in shaping rumen microbiota and metabolism, elucidating a nuanced relationship between dietary components, bacterial community structure, and metabolic output. This offers a more detailed understanding of rumen function and the potential for high-precision dietary management in lactating cows.

## 1 Introduction

Different sources of carbohydrates and the interaction of dietary carbohydrate sources and protein degradability can alter the ruminal fermentation of carbohydrates, including the yields and profiles of fermentation products (Hall, 2013). However, the effects of that interaction on the rumen microbial profile in dairy cattle and on animal performance have not been systematically evaluated. Information on ruminal responses to carbohydrates and rumen-degradable protein (RDP) will allow nutritionists to formulate rations that will better support higher production and greater efficiency.

Naturally occurring plant sugars such as sucrose and free glucose are rapidly utilized by rumen microbes (Weisbjerg et al., 1998), and there is evidence that they can affect the lactation performance of dairy cows differently from starch. Replacing dietary starch with purified sugars such as sucrose or with feed sources such as molasses, in which sucrose predominates and lesser amounts of glucose and fructose are present (Binkley and Wolfrom, 1953), can increase milk fat production (Broderick et al., 2008; Penner and Oba, 2009). However, the mode of action of such supplementation is not well understood. The animal response to the consumption of sugars such as sucrose may differ from responses to other water-soluble carbohydrates such as fructans, which are found in cool-season grasses, or the associated oligofructose, which is available commercially; the latter has been used to induce laminitis in dairy cattle (Danscher et al., 2009).

The impact of plant sugar or starch sources and the level of RDP on rumen fermentation or bacterial community composition (BCC) *in vivo* has not been well characterized. Increasing peptide RDP increases the yield of microbes per unit of carbohydrate for glucose (Hall, 2017). Although rapid fermentation can occur, most *in vivo* studies report that rumen pH is not depressed by feeding sugars (Oba, 2011). Compared with starch sources, ruminal short-chain fatty acid (SCFA) profiles with sucrose supplementation showed an increased molar proportion of butyrate (Ribeiro et al., 2005), although this response can vary. Replacing dietary corn grain with liquid molasses decreased ruminal concentrations of branched-chain SCFA and urinary excretion of urea-N as a percentage of N intake (Zanton and Hall, 2022). Such responses are normally correlated with improved ruminal N efficiency, but those associated with sucrose feeding did not increase microbial N flow from the rumen (Broderick et al., 2008).

An unanswered question is how the ruminal BCC contributes to the noted ruminal changes according to dietary carbohydrates and protein. SCFAs serve as good indicators of metabolic activity because they are the primary products of microbial fermentation. Changes in BCC and in molar proportions of SCFA have a bidirectional relationship in the rumen. Ruminal BCC is highly responsive to changes in the physical, chemical, and predatory environment created by protozoa in the rumen, as well as by the genetics of the host animal (Benson et al., 2010; Weimer et al., 2010). Plant sugars are water soluble, whereas native plant starch is insoluble and may be associated with the particles of grain from which it is derived. The differences in the solubility of these carbohydrates suggest that they may be associated with microbial responses in the liquid vs. solid digesta with which they associate. Different dietary regimes are a primary cause of changes in the ruminal environment, thus affecting ruminal BCC (Dehority and Orpin, 1997; Mohammed et al., 2014). With a ruminant’s estimated dependence on SFCA for up to 80% of the energy (Bergman, 1990) and 50% of the protein (Doi and Kosugi, 2004) they require, changes in ruminal BCC could affect an animal’s production (Jewell et al., 2015). The central goal of this study was to evaluate changes in rumen BCC as influenced by altering dietary carbohydrates and protein in lactating Holstein cows. We hypothesized that replacing starch-rich corn grain with different levels of plant sugar-rich molasses would modulate the BCC of cattle in favor of fibrolytic and plant sugar-degrading microbes at the expense of amylolytic bacteria, with different results in rumen liquid and solid fractions. As a secondary objective in this research, we evaluated the correlation between fermentation products and identified genera.

## 2 Materials and methods

### 2.1 Animal trial and treatments

The study was conducted at the USDA-ARS research dairy farm in Prairie du Sac, WI, under protocols approved by the University of Wisconsin–Madison Institutional Animal Care and Use Committee. The animal study is described in detail in the study by Zanton and Hall (2022). In a split-plot replicated 3 × 3 Latin square design, with periods of 28 days, the six experimental diets provided a 2 × 3 factorial arrangement of treatments. Diets had either a higher level of RDP (HiRDP) or a lower level of RDP (LoRDP) and contained a molasses (M) concentration of 0%, 5.25%, or 10.5% of dry matter (DM) substituting dry corn grain (**Table 1**), thus modifying the level of starch and sugar content of the diets. “Sugar” content was approximated as water-soluble carbohydrates plus total sugars as invert (WSC+TSI). Diets were designed to be isonitrogenous, with the same forage content, and similar in neutral detergent fiber (aNDFom) content. Dietary RDP was changed by replacing highly degradable soybean meal with less degradable expeller soybean meal. Dietary RDP supply calculated with the diet evaluation program of the Dairy National Research Council (2001) estimated the RDP concentrations of HiRDP and LoRDP diets to be 100.0 g/kg and 92.6 g RDP/kg of diet DM, respectively, and 67.7 g/kg and 73.0 g rumen-undegraded protein/kg of diet DM, respectively. In all, 12 ruminally cannulated, multiparous Holstein cows (parity 2.25 ± 0.62; 185 ± 56 days in milk; 41.3 ± 6.3 kg milk/day initially) were allocated to HiRDP or LoRDP treatments and, within those whole-plot treatments, were assigned to 3 × 3 Latin square molasses/corn grain treatment sequences with 28-day periods. The first 21 days of each period were for adaptation to the dietary treatment and the last 7 days were for sample collection. The whole-plot factor was the level of dietary RDP. Six cows remained on either HiRDP or LoRDP and received the three diets differing in molasses/corn grain formulation over three periods. Cow was the experimental unit. Cows were housed in straw-bedded tie stalls and fed individually once daily, with *ad libitum* access to feed and water. Refusals (approximately 10% of offered feed) were collected and weighed daily. Subsamples of feed offered, feed refused, and forage were collected daily and composited by week, and concentrates were sampled once weekly.

**Table 1:** Ingredients and chemical composition of mixed diets fed during the last week of each period of the 3 × 3 Latin square with cannulated cows fed two levels of rumen-degradable protein and three levels of molasses (M)^1^.

	HiRDP^3^			LoRDP^3^		
Item (% of DM, unless indicated)^2^	0%M^3^	5.25%M	10.5%M	0%M	5.25%M	10.5%M
Brown midrib corn silage	34.6	34.6	34.4	34.7	34.5	34.6
Alfalfa silage	21.2	21.1	21.0	21.2	21.1	21.2
Dried ground corn	18.6	14.3	10.0	18.7	14.5	10.0
Cane molasses	0.0	5.1	10.4	0.0	5.2	10.3
Soybean hulls	7.5	6.8	6.0	6.5	5.9	5.3
Soybean meal	13.0	13.2	13.5	6.4	6.3	6.4
Expeller soybean meal	0.0	0.0	0.0	7.4	7.5	7.7
Distillers grains	2.0	2.0	2.0	2.1	2.0	2.0
Limestone	0.27	0.14	0.00	0.27	0.14	0.00
K–Mg–S mineral	0.27	0.14	0.00	0.27	0.14	0.00
Vitamin and mineral premix	2.6	2.6	2.6	2.6	2.6	2.6
DM (% of diet as fed)	53.90	53.36	52.87	53.86	53.38	52.79
OM	91.56	91.06	90.59	91.60	91.11	90.63
Ash	8.44	8.94	9.41	8.40	8.89	9.37
WSC +TSI	5.57	8.45	11.5	5.61	8.54	11.5
Starch	27.6	24.4	21.2	27.7	24.5	21.4
NDF	26.9	26.0	25.0	27.0	26.1	25.3
CP	17.4	17.4	17.4	17.4	17.3	17.3
RDP (% of CP)	59	60	60	55	56	56

^1^Chemical composition is based on actual feeding amounts and chemical analysis of the feed ingredients.

^2^CP, crude protein; DM, dry matter; NDF, ash-free neutral detergent fiber; OM, organic matter; TSI, total sugars as invert; WSC, water-soluble carbohydrates. Vitamin and mineral premix contained 523 mg/kg monensin. Rumen-degradable protein calculated using NRC (2001) model from diets and mean animal DM intakes.

^3^HiRDP, higher rumen-degradable protein; LoRDP, lower rumen-degradable protein; M, molasses. K-Mg-S = sulfate forms of potassium and magnesium.

### 2.2 Ruminal sampling

Ruminal solid and liquid digesta samples for rumen BCC analysis were collected from each cow on 3 successive days in each period (days 26–28), 6 hours after morning feeding, and were pooled within phase (solid or liquid) by cow within a treatment period. Whole rumen contents were manually sampled from the medial ventral rumen at 1400 hours (midway between the a.m. and p.m. feedings) using a 250-mL plastic cup and separated by squeezing through four layers of cheesecloth to isolate rumen liquid from the rumen solids. Rumen liquid was defined as the liquid squeezed out of the whole rumen contents through the cheesecloth, of which 15 mL was retained for subsequent processing. Rumen solids were defined as the content that was retained on the cheesecloth after the rumen fluid was removed, which was packed tightly into a separate 50-mL conical tube for subsequent processing. Samples were stored on dry ice in a −20°C freezer until all 3 days of samples were collected; then they were transferred for storage at −80°C until processing and analysis. The liquid and solid samples from the 3 days of sampling within a period were combined by cow by digesta type into a 50-mL conical tube for the liquid and a 250-mL wide-mouth bottle for the solids and frozen at −80°C.

Note that the pooled samples were not mixed at this point. Mixing of samples was carried out after the thawing of the complete pooled sample at the start of the DNA isolation procedure. For ruminal pH, NH_3_, total amino acid (TAA), and SCFA analysis, rumen fluid was also removed from three locations in the rumen (anterior dorsal, medial ventral, and posterior dorsal) 6 hours after morning feeding on day 27 using a sampling probe. Rumen fluid pH was measured immediately, and a subsample of liquid frozen at −20°C was retained for further analysis.

### 2.3 Analysis of rumen samples

Rumen samples used in analyses of SCFA, NH_3_, and TAA were thawed at room temperature and centrifuged (15,300 × g for 20 minutes at 4°C). The supernatant was analyzed by flow-injection analyses (Lachat Quik-Chem 8000 FIA; Lachat Instruments, Milwaukee, WI) to determine NH_3_, using a phenol-hypochlorite method (Lachat Method 18-107-06-1-A; Lachat) and TAA as leucine equivalents. Ruminal SCFAs were determined by high-performance liquid chromatography (HPLC) (Weimer et al., 1991).

Data were analyzed in SAS version 9.4 (SAS Institute Inc., Cary, NC) as a split-plot Latin square design with fixed effects of the period, protein degradability, molasses level, and the interaction between protein degradability and molasses level. Cow within protein degradability was treated as a random effect. Significance was declared when *p* < 0.05 and tendency when 0.05 ≤ *p* ≤ 0.10.

### 2.4 DNA isolation

Total genomic DNA from both separately pooled liquid and solid samples of ruminal contents were isolated as follows. Samples were thawed in a water bath at room temperature, and then extracted separately following mechanical disruption and a hot/cold phenol extraction protocol as described by Stevenson and Weimer (2007). This procedure is very similar to the phenol-chloroform with bead beating II (PCSA, phenol:chloroform:isoamyl alcohol) method of Henderson et al. (2013). In brief, microbial DNA was extracted from the liquid phase directly, but the solid phase (50 mL of pooled rumen solids) was first homogenized in a blender with chilled extraction buffer (100 mM Tris/HCl, 10 mM EDTA, 0.15 M NaCl pH 8.0), centrifuged for 15 minutes at 500 × g, and filtered through four layers of cheesecloth to remove large particles. Filtrate was centrifuged for 1 hour at 5,000 *×* g to collect loosened, fiber-adherent cells. Solid-phase digesta was not rinsed during processing to avoid likely loss of weakly adherent bacteria. Pooled ruminal liquid (15 mL each from 3 collection days in a period) was centrifuged directly to collect cells. For each collected cell pellet, 1 mL was mechanically disrupted with 0.5 g of 0.1-mm zirconia/silica beads (BioSpec Products, Bartlesville, OK) with 50 μL of 20% SDS and 700 μL of equilibrated cold phenol on a Mini-Beadbeater (Biospec Products, Bartlesville, OK) for 2 minutes for lysis of the microbial cells, then placed in a 60°C water bath for 10 minutes and beaten again. Separation of the phases by centrifugation at 4°C was followed successively by three phenol extractions and two 25:24:1 phenol–chloroform–isopropanol extractions, with final overnight ethanol precipitation of the DNA. DNA was stored in varied resuspension volumes of DNase-free water based on the expected yield. After quantification of DNA concentration on a NanoDrop spectrophotometer (Thermo 179 Scientific, Wilmington, DE), samples were stored at 4°C before preparation of the DNA library.

### 2.5 Amplification and sequencing of bacterial 16S rRNA genes

Universal primers flanking the variable 4 (V4) region of the bacterial 16S rRNA coding region were used to perform PCR (Kozich et al., 2013). A total of 50 ng of DNA, 5 pmol of each primer, 12.5 µL of 2X HotStart ReadyMix (KAPA Biosystems, Wilmington, MA), and water to a total volume of 25 µL were used. Cycling conditions were as follows: initial denaturation of 95°C for 3 minutes; 25 cycles of 95°C for 30 seconds, 55°C for 30 seconds, and 72°C for 30 seconds; and a final extension at 72°C for 5 minutes. Gel electrophoresis was performed using a 1.0% low-melt agarose gel (National Diagnostics, Atlanta, GA), and amplified DNA was extracted from the gel using a ZR-96 Zymoclean Gel DNA Recovery Kit (Zymo Research, Irvine, CA). Extracted DNA was quantified in duplicate on 96-well microplates in accordance with the manufacturer’s instructions for the Quant-iT dsDNA Broad-Range Assay Kit, using reagents from a Qubit dsDNA Assay Kit (Thermo Fisher Scientific, Waltham, MA), read on a Synergy 2 Multi-Mode Reader (BioTek, Winooski, VT) after a programmed 3-second shaking period and a 2-minute incubation at 22°C. The extracted DNA was equimolarly pooled, combined with approximately 10% PhiX control DNA, and then sequenced on an Illumina MiSeq (Illumina, San Diego, CA) using an Illumina 2 × 250 bp paired-end v2 sequencing kit with custom sequencing primers as described by Kozich et al. (2013). After publication, all sequences from this project will be deposited in the National Center for Biotechnology Information’s Short Read Archive and made publicly available under accession number PRJNA953606.

### 2.6 Sequence cleanup

Sequence demultiplexing was performed according to sample-specific indices on the Illumina MiSeq. The program mothur (v.1.38.1) was used for sequence processing and data analysis (Schloss et al., 2009) following a protocol developed by Kozich et al. (2013). All code used in mothur is provided in **Supplemental Text 1**. Paired-end sequences were combined to form contigs and poor-quality sequences were removed using screen.seqs (maxhomop = 0, maxhomop = 8, minlength = 200, maxlength = 500) (e.g., sequences with ambiguous base pairs, homopolymers greater than 8 bp, and sequences shorter than 200 bp and longer than 500 bp were eliminated). The unique sequences were aligned with the SILVA 16S rRNA gene reference alignment database (v. 119) (Pruesse et al., 2007). Sequences with two or fewer different base pairs were considered the same and grouped by pre.cluster (diffs = 2) to reduce error, and chimeras were detected and removed by chimera.uchime and remove.seqs (Edgar et al., 2011). Sequences classified as Eukaryota, Archaea, Cyanobacteria, or unknown were removed from all subsequent analyses using the remove.lineage command. Singletons were removed by using the split.abund command with cutoff = 1. Sequences were clustered into 95% operational taxonomic units (OTUs) by the average neighbor algorithm using the cluster.split command. Classification of OTUs was performed based on the GreenGenes database (DeSantis et al., 2006), August 2013 release, with a bootstrap cutoff of 80.

### 2.7 Sequence analysis and statistical analysis

After grouping bacterial sequences into OTUs, Good’s coverage (Good, 1953) was calculated for all samples. Comparisons of taxa-relative abundances among the 12 cows, encompassing the six experimental diet types (A: 10.5% M + HiRDP, B: 5.25% M + HiRDP, C: 0% M + HiRDP, A(−): 10.5% M + LoRDP, B(−): 5.25% M + LoRDP, C(−): 0% M + LoRDP) and two ruminal phases (liquid and solid) were normalized using the normalized.shared command, with specified parameters (method = total group, norm = lowest number of sequences). The smallest number of reads per sample was 18,686. OTU counts and coverage metrics were obtained using summary.single (calc = bergerparker-chao-shannon-simpson-ace-coverage) from the normalized data. Different OTUs belonging to the same phyla, families, and genera in both liquid and solid phases were summarized in Python v.3.5.0 based on OTU counts, and taxonomy files were obtained using standard operating procedures (SOPs) for mothur (Kozich et al., 2013).

Alpha diversity (community diversity in each period within individual animals) was assessed using Chao’s estimate of species richness (Chao, 1984) and Shannon’s diversity index (Shannon, 1948) using three methods: mothur and the R packages vegan and pyloseq. Prior to alpha diversity metric analysis, the normality assumption of the data was verified statistically using the Shapiro–Wilk test. Because Simpson’s diversity is usually skewed, the inverse Simpson was determined. If Shannon’s diversity metric was found to be normally distributed, it was assessed by ANOVA. However, if Chao richness metrics were found to be non-normally distributed, these richness metrics were analyzed using a Kruskal–Wallis rank sum test, as the non-parametric equivalent of ANOVA (Kruskal, 1964), using four categorical variables of interest: digesta type (liquid, solid), RDP level (LoRDP and HiRDP), molasses level (0%, 5.25%, and 10.5%), and diet (RDP by molasses interaction). Alpha diversity and richness were assessed by ANOVA, with reduced models produced by stepwise removal of the highest *p*-value terms until the model had only individual variables and significant interaction terms.

Pairwise comparisons between Shannon diversity metrics of groups and corrections for their multiple comparisons were done by Tukey’s honest significance test for ANOVA. However, pairwise comparisons by *t*-test using false discovery rate (FDR) correction for multiple comparisons were computed using the Wilcoxon rank sum test as the non-parametric equivalent of a *t*-test for Chao richness metrics, with the two-level categorical variables of phase and RDP in R v.3.2.1 (R Development Core Team, 2011).

Beta diversity (differences in community composition between samples) was assessed by using non-metric multidimensional scaling to visualize differences as distance between samples calculated as the Bray–Curtis metric (Bray and Curtis, 1957) in R (vegan package, Oksanen et al., 2016).

Changes in total community structure (relative abundance, Bray–Curtis metric) and composition (presence/absence, Jaccard metric; Jaccard, 1912) were assessed using permutational multivariate analysis of variance (PERMANOVA). As with ANOVA/GLM of alpha-diversity, all variables that could interact were included in one model. Pairwise comparisons between each group were quantified with PERMANOVA, and *p*-values were FDR-corrected. As a statistical test of beta-diversity, non-parametric multidimensional scaling (nMDS) gives us a visual display of beta-diversity, but it does not test for statistical differences. Therefore, PERMANOVA and analysis of similarity (ANOSIM) were performed in R (vegan package). These tests indicate whether the overall microbial community differs by variables of interest, including digesta type, RDP level, and molasses level. R code is shown in **Supplementary Texts 2**, **3**.

The cumulative contribution of the most influential species was determined by testing OTUs that differ by digesta type, molasses level, and RDP level, and the first non-arbitrary selection of OTUs/taxa was obtained by similarity percentages (SIMPER) to decrease the number of OTUs of interest. SIMPER was performed using a cutoff of 0.01, which identifies any OTU that individually contributes more than 1% to SIMPER. B y doing so, OTUs that were the largest contributors to beta-diversity measures were identified by SIMPER as cumulatively explaining > 70% of the variation between pairwise comparisons (these OTUs were the most abundant and/or most variable OTUs in the data set). Running multiple similar tests requires an FDR correction, and correcting across all OTUs that contribute greater than 1% (793 in this data set) would most likely result in no significant results after the FDR correction (**Datasheet 1**). Given that OTUs are cumulative, the contribution of each OTU subtraction was determined using the value of the previous OTU. A total of 10 OTUs were found that may differ between liquid and solid fractions using a comparison with the percentage cutoff =1 (meaning up to a cumulative 100%) and removing OTUs that contribute less than 1% to diversity in the SIMPER analysis (FDR-Kruskal *p* < 0.05). However, these are only the OTUs that contribute most heavily to Bray–Curtis measures between digesta type groups, and they are not necessarily significantly different.

Rumen fermentation variables and rumen bacterial communities per cow per period were analyzed using the MIXED procedure of SAS v.9.4 (SAS Institute, Cary, NC). Period, RDP level, molasses level, and molasses × RDP interaction were designated as fixed effects in the model, and cows were assigned as a random effect. Contrasts were used to determine the linear and quadratic effects of molasses level and molasses × RDP interaction. For ruminal BCC, the 30 genera with the greatest relative abundance were analyzed using the MIXED procedure of SAS v.9.4 with a random cow effect and fixed effects of phase (solid vs. liquid), molasses level, and RDP level. Least squares means for fixed effects were determined and declared significant when *p *< 0.05 and as tendencies at 0.05 ≤ *p *< 0.01. Correlations between the relative abundance of microbial taxa at the genus level and pH, molar proportions of organic acids, and millimolar concentrations of NH_3_ and TAA were determined using the RCORR function in R.

## 3 Results

### 3.1 Rumen fermentation variables

The chemical analysis of rumen liquid samples taken 6 hours post feeding is summarized in **Table 2**, with original data in **Datasheet 4**. At 6 hours post feeding, ruminal pH, propionate and valerate molar proportions, and ammonia concentration were not affected by diet (**Table 2**). Acetate molar proportion had a tendency for a quadratic effect of molasses level (*p *=* *0.075) and was lowest at the highest molasses concentration. Butyrate molar proportion had a quadratic effect of molasses inclusion (*p *=* *0.017), with its molar proportion highest at 10.5% molasses. Lactate molar proportion increased with increased protein degradability (*p *=* *0.042). The concentration of TAA tended to be greater with high RDP (*p *=* *0.054).

**Table 2:** Dietary treatment effects on rumen fermentation responses at 6 hours post feeding for cannulated cows fed two levels of rumen-degradable protein and three levels of molasses as a percentage of dry matter.

		Molasses level				*p-*values^3^				
							Carbohydrate		Interaction	
Responses^1^	Protein^2^	10.5%	5.25%	0%	SEM	Protein	L	Q	L	Q
pH	HiRDP	6.06	6.07	6.04	0.098	0.882	0.986	0.623	0.125	0.307
	LoRDP	5.99	6.07	5.96						
Acetate (mol %)	HiRDP	65.2	65.70	66.4	1.272	0.478	0.432	0.075	0.938	0.698
	LoRDP	66.2	66.80	67.9						
Butyrate (mol %)	HiRDP	12.50	12.30	11.6	0.386	0.910	0.657	0.017	0.857	0.875
	LoRDP	12.40	12.30	11.6						
Propionate (mol %)	HiRDP	20.0	20.2	20.6	0.986	0.675	0.655	0.805	0.418	0.170
	LoRDP	20.2	19.60	19.2						
Valerate (mol %)	HiRDP	1.41	1.41	1.37	0.070	0.113	0.649	0.409	0.647	0.951
	LoRDP	1.27	1.32	1.25						
Lactate (mol %)	HiRDP	0.51	0.33	0.14	0.155	0.042	0.373	0.235	0.807	0.409
	LoRDP	0.10	0.00	0.00						
Ammonia (mM)	HiRDP	5.80	6.04	5.44	1.060	0.223	0.956	0.467	0.707	0.990
	LoRDP	4.52	4.19	3.86						
TAA (mM)	HiRDP	1.91	1.87	1.50	0.248	0.054	0.550	0.168	0.640	0.675
	LoRDP	1.49	1.23	1.15						

^1^ TAA, total amino acids.

^2^ HiRDP, higher rumen-degradable protein; LoRDP, lower rumen-degradable protein.

^3^ Contrasts: L, linear; Q, quadratic.

### 3.2 Bacterial community analysis

All ruminal liquid and solid digesta samples were used to assess bacterial community content by next-generation sequencing using Illumina’s MiSeq platform, with 2 × 250 bp reads of the V4 (variable 4) region of the 16S rRNA genes. The sequence cleanup process resulted in a total of 4,003,695 high-quality bacterial sequences from originally 4,650,921 sequences in mothur. Good’s coverage was greater than 99% for all samples.

### 3.3 Exploring diversity and richness metrics

#### 3.3.1 Alpha diversity

Alpha statistics calculated by three methods—mothur software and the vegan and pyloseq R packages—gave identical Shannon index values. According to the Shapiro–Wilk test distribution, the Shannon diversity metric was detected as normally distributed with *w-statistic* = 0.97 and a non-significant *p*-value of 0.151; however, Simpson diversity metrics and ACE and Chao richness metrics were not normally distributed. (**Table 3.1**).

**Figure 1:**
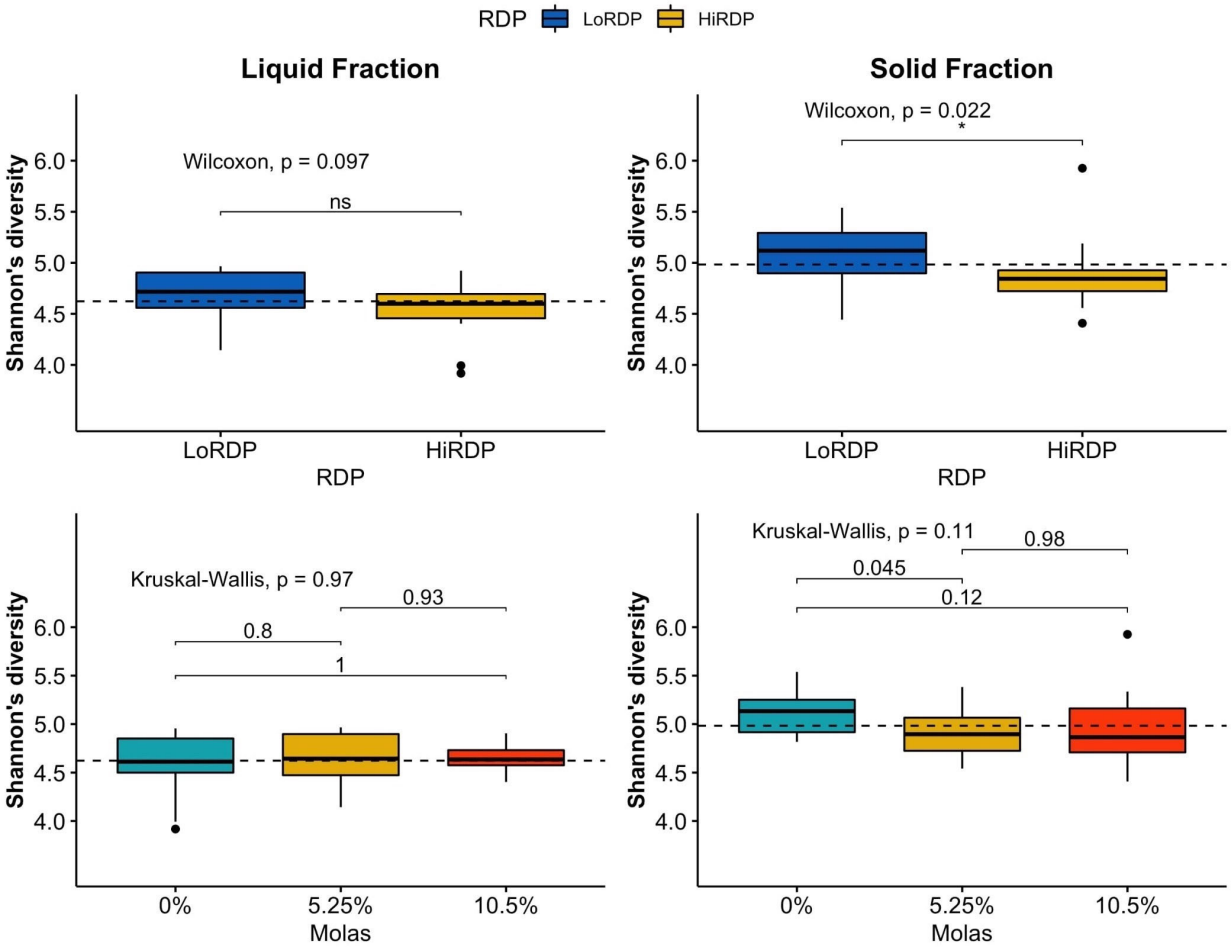
Boxplot of alpha diversity statistics expressing evenness (Shannon index) of bacterial communities in solid and liquid ruminal contents at two RDP levels (LoRDP: less degradable, HiRDP: more degradable) and three levels of molasses replacing corn grain. Values above the lines between responses for carbohydrate feed treatments are *p*-values from mean separations. Plotted are interquartile ranges (IQRs; boxes), medians (dark lines in the boxes), and the lowest and highest values within 1.5 times IQR from the first and third quartiles (whiskers above and below the boxes). Outliers are shown as dots. * = mean differences significant at *p* < 0.05. ns = mean differences not significant at *p* > 0.05.

**Figure 2:**
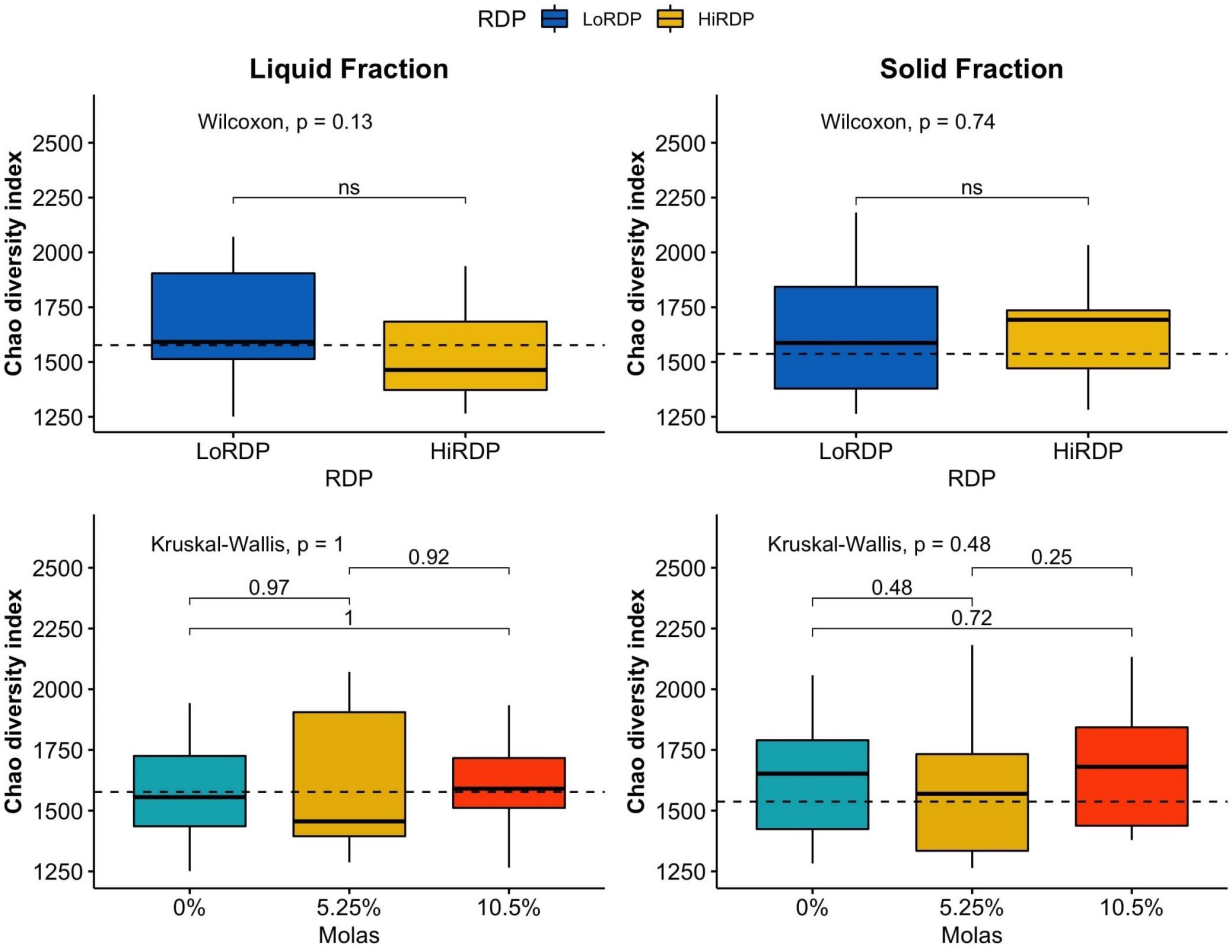
Boxplot of alpha diversity statistics expressing richness (Chao richness) of bacterial communities in solid and liquid ruminal contents at two RDP levels (LoRDP: less degradable, HiRDP: more degradable) and three levels of molasses substituted for corn grain. Values above the lines between responses for carbohydrate feed treatments are *p*-values from mean separations. Plotted are interquartile ranges (IQRs; boxes), medians (dark lines in the boxes), and the lowest and highest values within 1.5 times IQR from the first and third quartiles (whiskers above and below the boxes). Outliers are shown as dots. ns = mean differences not significant at *p* > 0.05.

The RDP level affected the alpha diversity of the ruminal bacterial community, as did digesta fraction, with *p*-values of 0.007 and < 0.001, respectively (**Table 3.2**, **Figures 1**, **2**). Interaction terms of the model were detected as digesta type:period and digesta type:period:molasses (**Table 3.2**). The bacterial community was more diverse in the rumen solids than in the rumen liquid (*p* < 0.001). When more RDP was provided in the diet, the community became less diverse (*p* = 0.015).

**Table 3.1:** Shapiro–Wilk normality test for alpha diversity and richness metrics.

	W-statistics	*p*-value
Shannon diversity metrics	0.97	0.151
Inverse Simpson diversity metrics	0.89	0.000
Chao richness metrics	0.96	0.015
Ace richness metrics	0.94	0.002

**Table 3.2:** ANOVA for alpha diversity (Shannon).

	df	Sums of squares	Mean Square	F-value	Pr(>F)
RDP	1	0.505	0.505	7.878	0.007
digesta.type	1	2.273	2.273	35.452	<0.001
digesta.type:Period	4	0.552	0.138	2.154	0.087
digesta.type:Period:molas	12	1.392	0.116	1.809	0.071
Residuals	52	3.334	0.0641		

Model fit: shannon ~ RDP + digesta.type + digesta.type: Period + digesta.type:molas:Period.

**Table 3.3:** Kruskal–Wallis rank sum test results for alpha richness (Chao).

	χ^2^-value	Pr (> χ^2^)
RDP	5.613	0.018
Molasses	1.335	0.513
Digesta type	0.805	0.370
Period	0.531	0.767

As the molasses level increased and the amount of corn grain declined, bacterial diversity was unaffected at both RDP levels, with no clear effect of the interaction of digesta type and molasses level on diversity. The effect of digesta type was clearer than the effect of the RDP level. Liquid digesta samples had lower Shannon’s diversity than solid samples. When compared by level of RDP, the highest community diversity was observed with LoRDP at zero or intermediate levels of molasses. When compared by fraction, the highest community diversity was obtained in solid fractions with no molasses.

Dietary supplementation with more RDP decreased the richness (*p* < 0.01) and diversity (*p *=* *0.01) of ruminal BCC in both liquid and solid fractions, although dietary molasses had no clear effect on these measures (**Figure 1B**). There was no detected effect of the interaction of RDP and dietary molasses level on the richness and diversity of ruminal BCC.

**Figure 3:**
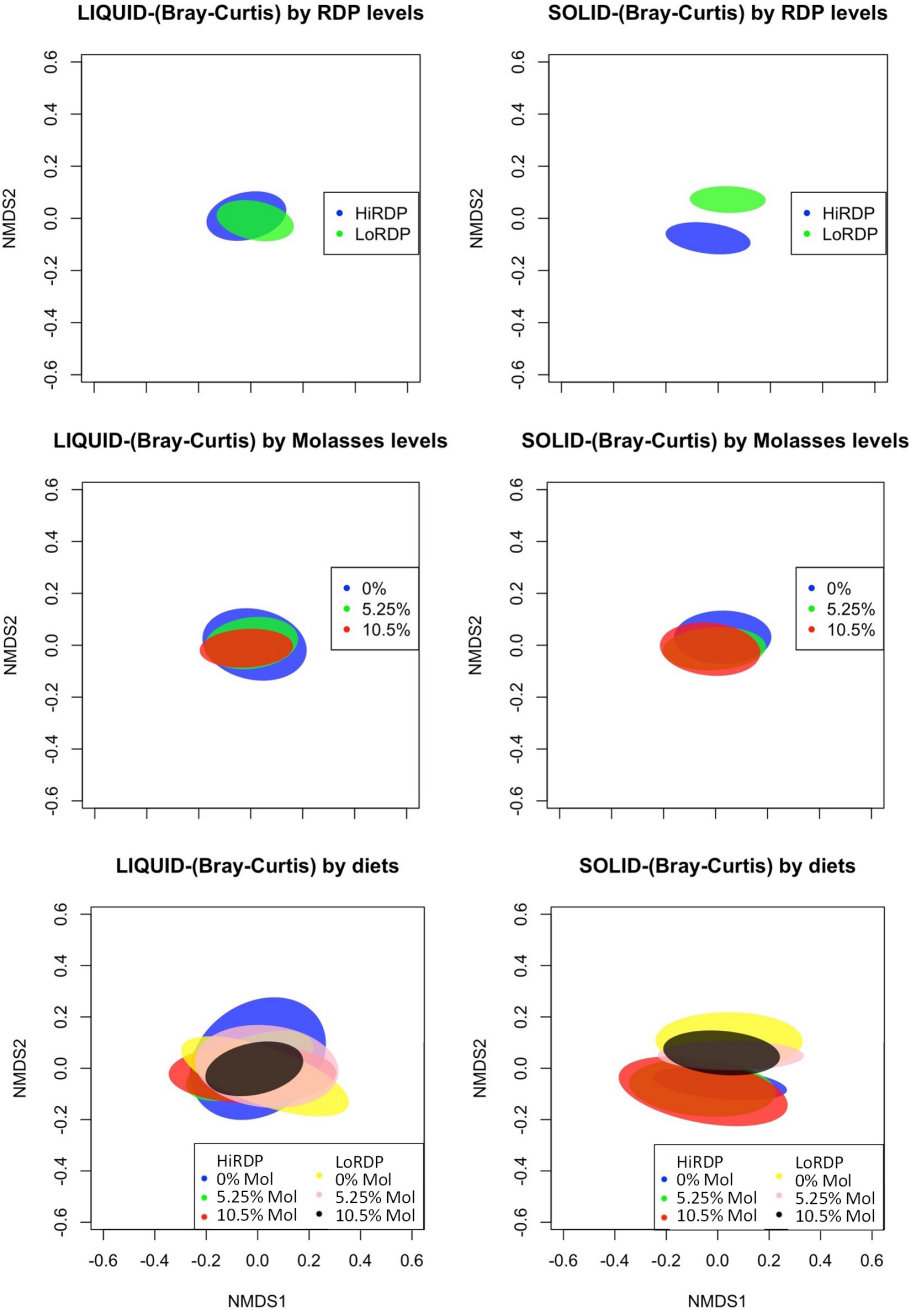
Standard error ellipses representing 99% confidence intervals of non-metric multidimensional scaling (NMDS) coordinates of the Bray–Curtis diversity metrics **(A)** For RDP level by digesta type in the liquid fraction. **(B)** For RDP level by digesta type in the solid fraction. **(C)** For molasses level by digesta type in the liquid fraction. **(D)** For molasses level by digesta type in the solid fraction. **(E)** For cross treatment of 3 × 2 factor (molasses level:RDP) by digesta type in the liquid fraction. **(F)** For cross treatment of 3 × 2 factor (molasses level:RDP) by digesta type in the solid fraction.

The bacterial community composition (BCC; i.e., which types of bacteria are present) and structure, in terms of abundance within the liquid and solid fractions of ruminal digesta, exhibited significant differences (**Figure 3** and **Datasheet 3** in the **Supplementary Material**). The nMDS plots showed that ruminal BCC did not clearly separate by the level of molasses supplementation in either liquid or solid fractions (*p* < 0.01, **Figure 3A**), indicating that ruminal BCC was not altered by molasses supplementation. However, dietary RDP level significantly changed ruminal BCC in the solid fraction, but not in the liquid fraction. (**Figures 3C, D**).

The richness of the bacterial community was also affected by the RDP level (*p* = 0.018) according to the Kruskal–Wallis rank sum test. Digesta type did not affect the richness of the bacterial community, unlike diversity (*p* = 0.37; **Table 3.3**). A high RDP level had a negative effect on the richness of the bacterial community (*p* = 0.016) when analyzed with a generalized linear model by selecting the quasi-Poisson distribution family to fit non-normally distributed Chao richness data.

#### 3.3.2 Beta diversity

Patterns of change in BCC across digesta types and RDP levels were apparent when analyzed using the non-metric multidimensional scaling (nMDS) plot of the Bray–Curtis dissimilarity metric using ellipses displaying 99% confidence intervals (**Figure 3**). Digesta type and RDP level were significant for the Bray–Curtis and Jaccard metrics, which indicates that the samples had different specific OTUs but similar taxa at the genus level.

The liquid- and solid-associated ruminal BCCs differed in both composition and structure (composition and abundance of ruminal BCC) (**Figure 3** and **Datasheet 3**). In the nMDS plots, shown in **Figures 3C, D**, ruminal BCCs with or without molasses supplementation were not clearly separated in either liquid or solid fractions (statistics shown in **Supplementary Table S1**), indicating that ruminal BCC was not altered by molasses supplementation. In contrast, dietary RDP levels changed ruminal BCC in the solid fraction, but not in the liquid fraction. (**Figure 3A**).

The PERMANOVA analysis results, shown in **Tables 4.1**, **4.2**, assess whether or not bacterial communities differed according to digesta type, molasses level, and RDP level. Bray–Curtis measures of community composition differed between solid and liquid samples (digesta type), with ANOSIM statistics of *R* = 0.90 and *p* = 0.0001. The homogeneity of dispersion among groups (digesta type and RDP level) was checked using betadisper, along with a subsequent permutation test.

**Table 4.1:** PERMANOVA for beta diversity (Bray–Curtis).

	df	Sums of squares	Mean Square	F.Model	*R* ^2^	Pr(>F)
RDP	1	0.127	0.127	3.082	0.027	0.021
molas	2	0.068	0.034	0.825	0.014	0.553
digesta.type	1	1.999	1.999	48.694	0.422	0.001
RDP:molas	2	0.036	0.018	0.439	0.008	0.943
RDP:digesta.type	1	0.037	0.037	0.905	0.008	0.425
molas:digesta.type	2	0.036	0.018	0.432	0.008	0.948
RDP:molas:digesta.type	2	0.016	0.008	0.191	0.003	1.000
Residuals	59	2.423	0.041		0.511	
Total	70	4.741			1.000	

**Table 4.2:** PERMANOVA for beta diversity (Jaccard).

	df	Sums of squares	Mean Square	F.Model	*R* ^2^	Pr(>F)
RDP	1	0.127	0.127	3.082	0.027	0.02
molas	2	0.068	0.034	0.825	0.014	0.576
digesta.type	1	1.999	1.999	48.694	0.422	0.001
RDP:molas	2	0.036	0.018	0.439	0.008	0.961
RDP:digesta.type	1	0.037	0.037	0.905	0.008	0.408
molas:digesta.type	2	0.036	0.018	0.432	0.008	0.944
RDP:molas:digesta.type	2	0.016	0.008	0.191	0.003	1.000
Residuals	59	2.423	0.041		0.511	
Total	70	4.741			1.000	

The results of that analysis were non-significant for digesta type (liquid vs. solid) and RDP level, at significance levels of *p* = 0.13 and *p* = 0.64, respectively (**Supplementary Table S1**). A non-significant result in betadisper is not necessarily related to a significant or non-significant result in adonis (PERMANOVA), because the two tests evaluate different hypotheses. The former tests the homogeneity of dispersion among groups (digesta type and RDP level, in our case), whereas the latter tests whether the composition among groups is similar or not. The centroids of two groups may have NMS at a very similar position in the ordination space, but if their dispersions are quite different, adonis will give a significant *p*-value; therefore, the result is heavily influenced not by the difference in composition between groups but by differences in composition within groups (heterogeneous dispersion) and, thus is a measure of beta diversity. In short, our results indicate that homogeneous dispersion for adonis was met, and, thus, it is certain that results from adonis are “real” and not an artifact of heterogeneous dispersions for digesta type and RDP levels. That said, the effect of digesta type on the Bray–Curtis and Jaccard metrics was different (*p* < 0.001 for both), with greater variation in liquid samples. Furthermore, BCC was shown to be slightly different in the high-RDP diet versus the low-RDP diet. The BCC of ruminal solids was distinct between the high-RDP and low-RDP diets (pairwise PERMANOVA, FDR-adjusted *p *=* *0.029, **Supplementary Table S3**), with greater variation within groups (**Figure 3B**).

### 3.4 Relative abundance at the genus level

Least-squares means of the relative abundance for 26 of 30 genera differed (*p* < 0.05) by fraction. The sum of significant genera in Bacteroidetes was found in greater abundance in the liquid (65.9%) and solid (44.4%) fractions and genera in Firmicutes were found in lesser abundance in the liquid (9.34%) and solid (21.1%) fractions.

In the solids, the most abundant genera averaged across samples, included *Prevotella* (42.2.%), *Succiniclasticum* (7.69%), *Butyrivibrio* (2.98%), *Ruminococcus* (2.93%), *Coprococcus* (2.79%), *Clostridium* (2.05%), *Treponema* (1.67%), *YRC22* (1.11%), and *CF231* (0.98%). In the liquid, the most abundant genera included *Prevotella* (62.11%), *Succiniclasticum* (4.87%), *CF231* (2.18%), *YRC22* (1.54%), *Treponema* (1.41%), *Ruminococcus* (1.15%), *Butyrivibrio* (0.76%), and *Coprococcus* (0.72%).

Unclassified genera were enriched in the solids at the low RDP level. At the phylum level, these unclassified genera were within the phyla Firmicutes (14 of 30 genera), Bacteroidetes (11 of 30), Spirochaetes (2 of 30), Proteobacteria (2 of 30), and Fibrobacterota (1 of 30). Within these phyla, the greatest percentages belonged to the Lachnospiraceae, Prevotellaceae, Ruminococcaceae, Succinivibrionaceae, and Spirochaetaceae families.

#### 3.4.1 Effects of digesta type and treatments on relative abundances at the genus level

Effects of dietary supplementation with two levels of RDP and three levels of dietary molasses replacing corn grain on the relative abundance of genera are presented in **Tables 5**, **6**. In the liquid, supplementation with more dietary RDP tended to decrease the relative abundances of *Coprococcus* (*p* = 0.099) and *Succinivibrio* (*p* = 0.10). However, *Prevotella* (*p* = 0.081) tended to increase with HiRDP. In addition, *Anaeroplasma* had the highest relative abundance with the 5.25%-molasses diet. There was a quadratic effect of molasses (*p* = 0.019) on *Anaeroplasma* and, at the extreme, the relative abundance of *Anaeroplasma* was also decreased by increasing molasses supplementation numerically. The relative abundances of *Succiniclasticum* (*p* = 0.018), *Ruminococcus* (*p* = 0.095), *Anaerovibrio* (*p* = 0.084), *Schwartzia* (*p* = 0.013), and *Shuttleworthia* (*p* = 0.054) decreased or tended to decrease with the supplementation of diets with molasses. Conversely, greater inclusion of corn grain in the diet increased the relative abundances of these genera. In the liquid fraction, *Butyrivibrio* (*p* = 0.089), *Anaerovibrio* (*p* = 0.036), *Selenomonas* (*p* = 0.081), and *Schwartzia* (*p* = 0.033) exhibited or tended to exhibit interaction effects from protein and carbohydrate feed treatments. In the solids, supplementation with more dietary RDP decreased the relative abundance of *YRC22* (*p* = 0.027) and *Pseudobutyrivibrio* (*p* =0.020) and also tended to decrease the relative abundance of *Succinivibrio* (*p* = 0.077). However, the relative abundance of *Succiniclasticum* (*p* = 0.003) increased with RDP supplementation. The relative abundance of *Desulfovibrio* (*p* = 0.006), *Oscillospira* (*p* = 0.009), *CF231* (*p* = 0.016), *YRC22* (*p* = 0.012), and *BF311* (*p* = 0.094) increased or tended to increase linearly with dietary molasses level. In contrast, the relative abundance of *Schwartzia* (*p* = 0.06) tended to decrease with increasing molasses level. Only one genus (*Moryella*) was affected by the interaction of dietary treatments (*p* = 0.090).

**Table 5:** Effect of RDP supplementation and dietary molasses levels on the relative abundance of ruminal bacterial genera in liquid digesta^1^.

	Experimental diet^2^					*p*-values^3^					
								Molasses		RD RDP × M	
Classification	RDP	10.5%M	5.25%M	0%M	SEM	M	RDP	L	Q	L	Q
*Prevotella*	HiRDP	68.02	63.05	60.60	2.405	0.274	0.081	0.127	0.635	0.142	0.902
	LoRDP	60.66	59.84	60.50							
*Succiniclasticum*	HiRDP	4.24	4.54	6.11	0.711	0.047	0.743	0.018	0.458	0.89	0.777
	LoRDP	4.03	4.58	5.70							
*Coprococcus*	HiRDP	0.57	0.559	0.73	0.097	0.667	0.099	0.388	0.835	0.125	0.192
	LoRDP	0.83	0.870	0.78							
*Butyrivibrio*	HiRDP	0.68	0.667	0.91	0.107	0.695	0.877	0.705	0.451	0.089	0.505
	LoRDP	0.84	0.763	0.699							
*Treponema*	HiRDP	1.09	1.67	1.49	0.259	0.283	0.951	0.226	0.302	0.767	0.538
	LoRDP	1.25	1.47	1.49							
*CF231*	HiRDP	2.17	2.32	2.23	0.299	0.785	0.697	0.646	0.608	0.478	0.923
	LoRDP	2.22	2.17	1.95							
*YRC22*	HiRDP	1.42	1.55	1.34	0.131	0.279	0.186	0.128	0.664	0.407	0.181
	LoRDP	1.80	1.58	1.54							
*Pseudobutyrivibrio*	HiRDP	0.238	0.196	0.203	0.037	0.748	0.146	0.453	0.955	0.735	0.353
	LoRDP	0.270	0.290	0.256							
*Ruminococcus*	HiRDP	1.02	1.00	1.26	0.132	0.205	0.358	0.095	0.557	0.785	0.457
	LoRDP	1.12	1.22	1.30							
*Lachnospira*	HiRDP	0.221	0.212	0.237	0.032	0.941	0.843	0.851	0.770	0.751	0.741
	LoRDP	0.219	0.218	0.215							
*Anaerovibrio*	HiRDP	0.149	0.179	0.143	0.025	0.055	0.617	0.084	0.077	0.036	0.256
	LoRDP	0.146	0.179	0.197							
*Clostridium*	HiRDP	0.461	0.451	0.513	0.061	0.971	0.109	0.848	0.884	0.239	0.509
	LoRDP	0.615	0.603	0.545							
*Selenomonas*	HiRDP	0.212	0.223	0.154	0.041	0.831	0.515	0.865	0.564	0.081	0.322
	LoRDP	0.207	0.221	0.255							
*Fibrobacter*	HiRDP	0.284	0.317	0.177	0.057	0.759	0.563	0.471	0.906	0.226	0.101
	LoRDP	0.240	0.179	0.267							
*Schwartzia*	HiRDP	0.119	0.137	0.124	0.021	0.040	0.486	0.013	0.899	0.033	0.195
	LoRDP	0.120	0.137	0.179							
*BF311*	HiRDP	0.073	0.121	0.089	0.019	0.084	0.667	0.813	0.029	0.392	0.356
	LoRDP	0.102	0.115	0.093							
*Shuttleworthia*	HiRDP	0.123	0.155	0.176	0.057	0.103	0.720	0.054	0.351	0.284	0.196
	LoRDP	0.183	0.157	0.199							
*Succinivibrio*	HiRDP	0.087	0.068	0.084	0.044	0.451	0.100	0.253	0.609	0.223	0.282
	LoRDP	0.106	0.197	0.190							
*Anaeroplasma*	HiRDP	0.186	0.282	0.258	0.032	0.015	0.686	0.066	0.019	0.673	0.836
	LoRDP	0.185	0.279	0.231							
*Oscillospira*	HiRDP	0.107	0.124	0.290	0.031	0.934	0.677	0.723	0.928	0.529	0.831
	LoRDP	0.138	0.133	0.132							
*RFN20*	HiRDP	0.109	0.194	0.176	0.029	0.091	0.669	0.163	0.082	0.424	0.868
	LoRDP	0.147	0.199	0.166							

^1^Only the genera with a relative abundance of higher than 0.1% and significantly affected by treatments are presented.

^2^10.5%M, 10.5% dietary molasses; 5.25%M, 5.25% dietary molasses; 0%M, 0% dietary molasses; HiRDP, higher rumen-degradable protein inclusion; LoRDP, lower rumen-degradable protein inclusion.

^3^Contrasts: L, linear; Q, quadratic.

**Table 6:** Effect of RDP supplementation and dietary molasses levels on the relative abundance of ruminal bacterial genera in solid digesta^1^.

	Experimental diet^2^					*p*-values^3^					
								Molasses		RDP × M	
Classification	Protein	10.5%M	5.25%M	0%M	SEM	M	Protein	L	Q	L	Q
*Prevotella*	HiRDP	42.55	44.13	43.52	1.906	0.442	0.245	0.211	0.838	0.482	0.585
	LoRDP	39.45	40.65	42.85							
*Succiniclasticum*	HiRDP	7.52	10.61	9.72	1.149	0.168	0.003	0.158	0.189	0.686	0.531
	LoRDP	5.31	6.64	6.54							
*Coprococcus*	HiRDP	2.98	2.75	2.93	0.279	0.557	0.556	0.295	0.797	0.410	0.444
	LoRDP	2.89	2.77	2.45							
*Butyrivibrio*	HiRDP	3.29	3.05	3.13	0.289	0.438	0.275	0.335	0.377	0.745	0.891
	LoRDP	3.03	2.66	2.71							
*Treponema*	HiRDP	1.73	1.85	1.49	0.207	0.085	0.981	0.137	0.099	0.961	0.869
	LoRDP	1.73	1.82	1.51							
*CF231*	HiRDP	0.975	0.904	0.775	0.109	0.052	0.135	0.016	0.897	0.925	0.486
	LoRDP	1.21	1.05	1.00							
*YRC22*	HiRDP	1.09	0.917	0.855	0.106	0.036	0.027	0.012	0.564	0.772	0.775
	LoRDP	1.39	1.26	1.19							
*Pseudobutyrivibrio*	HiRDP	0.583	0.449	0.449	0.096	0.401	0.020	0.202	0.650	0.689	0.587
	LoRDP	0.822	0.792	0.750							
*Ruminococcus*	HiRDP	3.17	2.58	2.86	0.245	0.459	0.485	0.415	0.333	0.808	0.316
	LoRDP	3.09	3.02	2.94							
*Lachnospira*	HiRDP	0.429	0.412	0.462	0.051	0.254	0.304	0.477	0.130	0.132	0.599
	LoRDP	0.565	0.454	0.478							
*Anaerovibrio*	HiRDP	0.168	0.166	0.163	0.041	0.351	0.181	0.316	0.276	0.231	0.275
	LoRDP	0.195	0.268	0.249							
*Clostridium*	HiRDP	2.19	2.00	1.92	0.179	0.094	0.942	0.032	0.821	0.865	0.697
	LoRDP	2.16	2.06	1.94							
*Selenomonas*	HiRDP	0.121	0.118	0.153	0.031	0.558	0.174	0.323	0.715	0.627	0.501
	LoRDP	0.177	0.188	0.188							
*Fibrobacter*	HiRDP	0.287	0.220	0.272	0.052	0.438	0.184	0.208	0.833	0.332	0.261
	LoRDP	0.375	0.358	0.261							
*Schwartzia*	HiRDP	0.102	0.130	0.125	0.018	0.066	0.461	0.066	0.110	0.981	0.957
	LoRDP	0.119	0.146	0.142							
*Desulfovibrio*	HiRDP	0.209	0.195	0.140	0.036	0.017	0.982	0.006	0.562	0.778	0.673
	LoRDP	0.223	0.185	0.140							
*BF311*	HiRDP	0.122	0.107	0.081	0.029	0.237	0.481	0.094	0.962	0.966	0.731
	LoRDP	0.151	0.123	0.111							
*Shuttleworthia*	HiRDP	0.313	0.373	0.333	0.117	0.624	0.974	0.341	0.891	0.180	0.228
	LoRDP	0.424	0.290	0.289							
*Ruminobacter*	HiRDP	0.067	0.040	0.059	0.032	0.328	0.312	0.197	0.424	0.340	0.643
	LoRDP	0.123	0.092	0.074							
*Moryella*	HiRDP	0.277	0.217	0.209	0.043	0.656	0.931	0.486	0.537	0.090	0.616
	LoRDP	0.215	0.227	0.245							
*Succinivibrio*	HiRDP	0.043	0.026	0.041	0.021	0.386	0.077	0.319	0.363	0.272	0.800
	LoRDP	0.068	0.077	0.101							
*Oscillospira*	HiRDP	0.157	0.116	0.124	0.026	0.013	0.281	0.009	0.087	0.660	0.707
	LoRDP	0.199	0.160	0.154							
*Bulleidia*	HiRDP	0.124	0.135	0.118	0.047	0.441	0.578	0.211	0.921	0.282	0.645
	LoRDP	0.132	0.085	0.059							
*Dialister*	HiRDP	0.012	0.085	0.040	0.050	0.674	0.943	0.462	0.653	0.130	0.125
	LoRDP	0.101	0.028	0.022							

^1^Only genera with a relative abundance higher than 0.1% and significantly affected by treatments were presented.

^2^RDP, effects of rumen degradable protein; M, effects of dietary molasses levels; RDP x M, the interaction between RDP and M; L, linear effect; Q, quadratic effect.

^3^Contrasts: L, linear; Q, quadratic.

#### 3.4.2 Effects of the sample fractions on the genus-level relative abundances

Of the top 30 genera detected, 8 were strongly associated with the liquid fraction, and 18 with the solid fraction, including *Butyrivibrio*, *Anaerostipes*, *Pseudobutyrivibrio*, *Clostridium*, *Coprococcus*, *Lachnospira*, *Bulleidia*, *Rumminococcus*, and *Succiniclasticum* (*p *< 0.0001). Interestingly, sample fraction had no effect on the relative abundance of *Fibrobacter*, which must attach to fiber in order to degrade it.

#### 3.4.3 Impact of RDP supplementation and molasses dietary inclusion on family-level relative abundances

The relative abundances of bacterial families in response to RDP supplementation and dietary molasses level were evaluated using OTU counts provided in **Supplementary Datasheet 2**, and the results are visualized in **Supplementary Figures 3A1, 3A2, 3B1,** and **3B2**. An increase in RDP supplementation reduced the relative abundance of the Succinivibrionaceae class and unclassified Gammaproteobacteria. In contrast, it resulted in a marked increase in Paraprevotellaceae, Prevotellaceae, and BS11 abundances, with marginal rise in Spirochaetaceae and order Clostridiales_unclassified abundances across both liquid and solid fractions. This is further represented in **Supplementary Figures 3A1, B**, where bar charts show the grouping of liquid and solid digesta samples by RDP levels and depict the top nine families. Furthermore, higher levels of RDP supplementation led to a decrease in the abundance of Paraprevotellaceae, unclassified phylum Firmicutes, Lachnospiraceae, unclassified order Bacteroidales, and phylum Bacteroidetes. It also contributed to a minor decrease in Prevotellaceae and Ruminococcaceae abundances, whereas no significant increase in the abundance of any family was observed. The inclusion of dietary molasses at 10.5% led to a decrease in the relative abundance of Prevotellaceae and unclassified Gammaproteobacteria, with a modest decline in Paraprevotellaceae. However, it resulted in an increase in Veillonellaceae abundance in the liquid fraction. On the other hand, in the solid fraction, the same level of dietary molasses led to an increase in the abundance of Prevotellaceae, Lachnospiraceae, Veillonellaceae, and unclassified phylum Firmicutes, while decreasing the abundance of phylum Bacteroidetes. These observations are further detailed in **Supplementary Figures 3B1, 3D**.

### 3.5 Cumulative contributions of most influential species

Using the SIMPER analysis, the OTUs that individually contributed more than 1% to Bray–Curtis measures between sample and dietary groups were selected, but the resulting OTUs may not be significantly different. To test significance, the relative abundance of OTUs across diet and sample groups was compared with a Kruskal–Wallis test; for example, OTU00001 occurred in all SIMPER digesta type comparisons and did, in fact, differ significantly by fraction (*p *< 0.0001). In this analysis, 10 OTUs were found to differ significantly between the solid and liquid fractions, whereas 4 OTUs differed by RDP level and 1 OTU differed by molasses level (**Datasheet 1**). A total of 5 of the 10 OTUs that differed significantly by sample fraction belonged to the genus *Prevotella*. One OTU each of *Coprococcus*, *Butyrivibrio*, *Succiniclasticum*, and the family BS11 were enriched in the solids (*p* < 0.0001), in addition to one OTU identified as *Clostridiales* (*p* < 0.0001). However, *Prevotella ruminicola* and other OTUs belonging to the genus *Prevotella* were enriched in the liquid. In a family-level taxonomic classification of this group, five OTUs belonged to the family Prevotellaceae, two belonged to Lachnospiraceae, and one belonged to Veillonellaceae, with the others belonging to BS11 and order Clostridiales (*p* < 0.0001). When grouped by RDP level, the significantly affected OTUs belonged to *Prevotella* (*p* < 0.05), and all were increased in the LoRDP diet (*p* < 0.05). An OTU classified as Lachnospiraceae was found at a significantly higher level in the 10.5% molasses diet than in the 0% molasses diet (*p* <.0001), and *Succiniclasticum* was decreased in the high-molasses diet (*p* < 0.05).

In testing OTUs that differed by digesta type, molasses level, and RDP level, the greatest number were found in the genus *Prevotella*, represented by five of the OTUs, followed by *Coprococcus*, *Succiniclasticum*, *Butyrivibrio*, and those unclassified at the genus level. These were classified at the order level as Clostridiales and Bacterioidales BS11 by SIMPER digesta type comparison and did, in fact, differ significantly by fraction (FDR-Kruskal *p* < 0.05, **Datasheet 1**). In contrast, at the same significance level of FDR-Kruskal *p* < 0.05, RDP and molasses levels did not contribute any specific OTUs, but at a threshold of FDR-Kruskal *p* < 0.10, there were some repeated or shared OTUs among the sample groups. For example, OTU840 belongs to the Tenericutes phylum with order RF39_unclassified, and OTU314 belongs to the phylum Firmicutes and order Clostridiales (see the list in **Datasheet 1**). Across the RDP levels, the relative abundances of OTUs that fell under the same genera were found to be primarily *Prevotella*, with 8 of 12 significant OTUs in this group. For molasses level, RF39_unclassified, which belongs to phylum Tenericutes, was significant with two OTUs, and Clostridiales of phylum Firmicutes was significant with three OTUs (**Datasheet 1**).

### 3.6 Correlations with ruminal BCC and molar proportion of short-chain fatty acids

**Figure 4:**
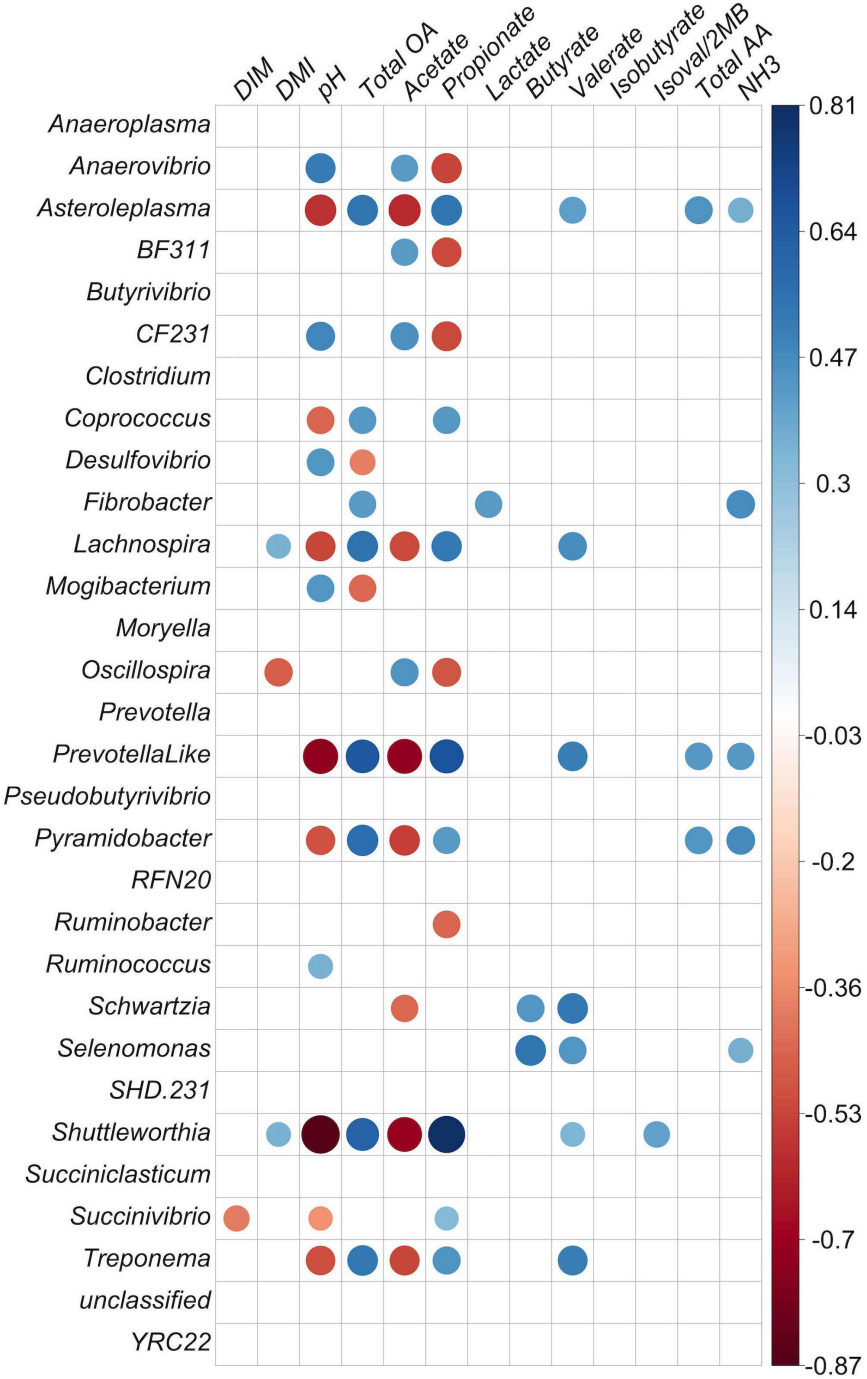
Correlation matrix of identified genera with at least 0.1% relative abundance and concentration of fermentation products in the liquid fraction. Positive correlations are shown in blue and negative correlations in red (*p* < 0.05). The color intensity and size of circles are proportional to the correlation values (*r*) within a correlation group. All ruminal short-chain-fatty acid (SCFA) data are presented as molar proportions except isoval/2MB: isovalerate/3-methylbutyrate and isobutyrate. Ammonia, TAA (total amino acids), and organic acids are given in mM. DIM, days in milk; DMI, dry matter intake; NH3, ammonia; total OA, total organic acids.

**Figure 5:**
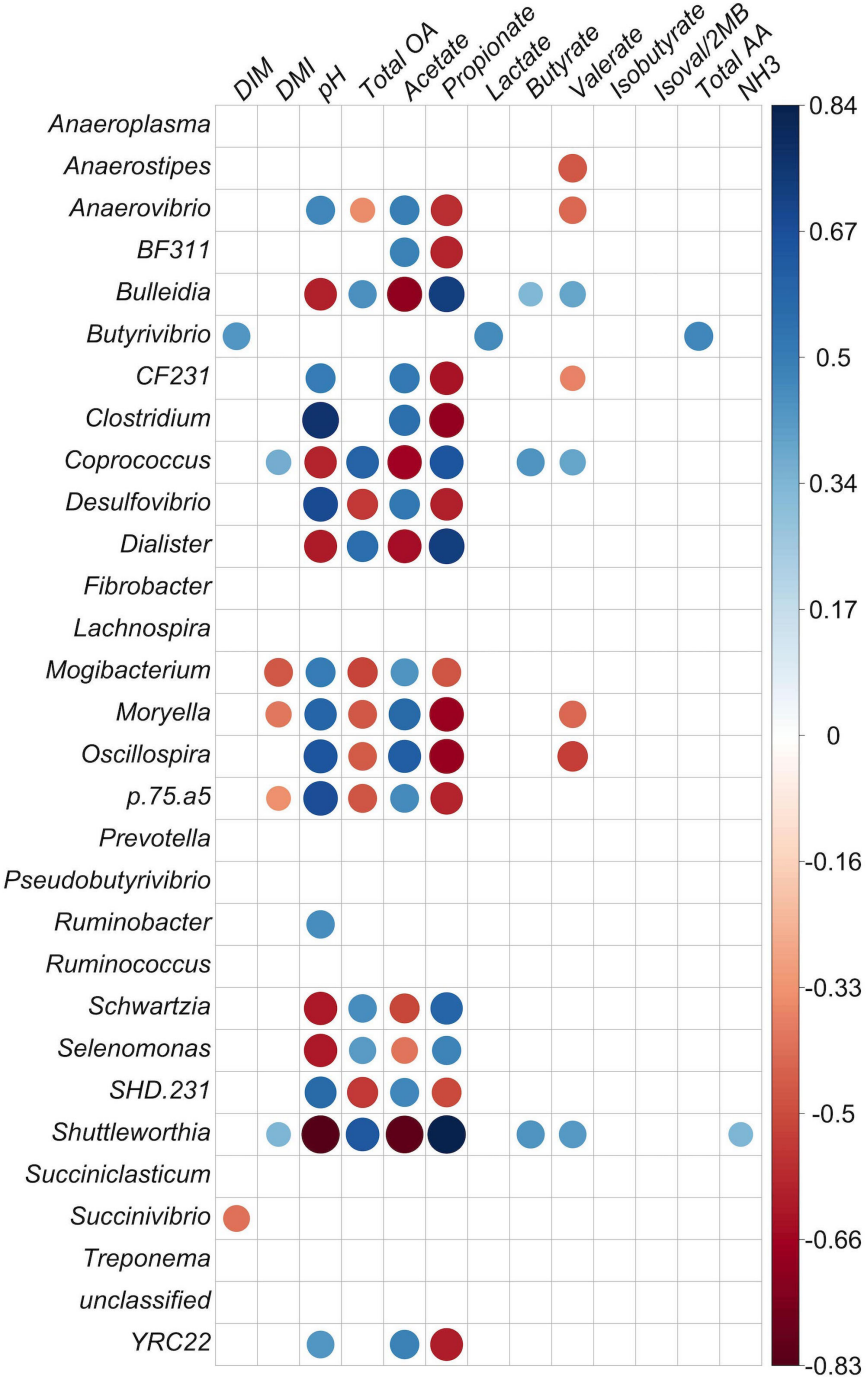
Correlation matrix of identified genera with at least 0.1% relative abundance and concentration of fermentation products in the solid particle-associated fraction. Positive correlations are shown in blue and negative correlations in red (*p* < 0.05). The color intensity and size of circles are proportional to the correlation values (*r*) within a correlation group. All ruminal short-chain-fatty acid (SCFA) data are presented as molar proportions except isoval/2MB: isovalerate/2-methylbutyrate and isobuytrate. Ammonia, TAA, total amino acids, and organic acids are given in mM. DIM, days in milk; DMI, dry matter intake; NH3, ammonia; total OA, total organic acids.

The correlation study was carried out at two different levels. One was the correlation between genus-level BCC and two rumen characteristics: pH and SCFA abundances. The other was the correlation between the out-level distance matrix and the same ruminal measures noted above, using the vegan::bioenv function in R with the Bray–Curtis similarity method and Kendall correlation values (*r*). Correlations between genus and rumen chemistry were also evaluated for communities from both the liquid and solid fractions (**Figures 4**, **5**, respectively).

In the liquid fraction, there were negative correlations between *Anaerovibrio* and both molar proportions of propionate (*p* = 0.001) and total SCFA (*p* = 0.038) molarities (**Figure 4**; *p* < 0.05). *Shuttleworthia*, *Prevotella*-like bacteria, *Lachnospira, Treponema*, *Lachnospira*, *Asteroleplasma*, *Pyramidobacter*, *Coprococcus, Succinivibrio*, and *Schwartzia* were positively correlated with propionate molar proportion (*p* < 0.05). Propionate molar proportion had a positive correlation with molar proportions of butyrate, valerate, dry-matter intake (DMI), and total concentrations of SCFA (*p* < 0.05). Propionate molar proportion was strongly negatively correlated with acetate molar proportion and pH, and negatively correlated with genera *CF231*, *Anaerovibrio, Oscillospira*, *Ruminobacter*, and *BF311* (*p* < 0.05). For acetate molar proportion, negative correlations were observed with the genera *Shuttleworthia*, *Asteroleplasma*, *Pyramidobacter*, *Treponema*, *Lachnospira*, *Schwartzia, Fibrobacter*, and *Prevotella*-like bacteria (*p* < 0.05), and also a tendency for a negative correlation with *Coprococcus* (*p* = 0.056). The same group, comprising *Asteroleplasma*, *Prevotella*-like bacteria, *Pyramidobacter*, *Treponema*, *Lachnospira*, *Schwartzia*, and *Shuttleworthia*, were found to be negatively correlated with acetate molar proportion and positively correlated with propionate molar proportion.

Butyrate molar proportion was negatively correlated with the genera *CF231* and *Mogibacterium* and with acetate and pH. Butyrate molar proportion was positively correlated with the genera *Asteroleplasma*, *Pyramidobacter*, and *Prevotella-*like bacteria, and also with molar proportion of propionate and total SCFA concentration. Lactate was positively correlated with *Fibrobacter* (*p* = 0.05) and strongly correlated with TAA (*r* = 0.90). However, it was negatively correlated with acetate (*p* = 0.03; *r* = −0.35). The importance of these observations is not apparent, as *Fibrobacter* produces acetate and succinate, but not lactate, as fermentation products, and has not been shown to either degrade or utilize amino acids for growth. Valerate was strongly negatively correlated with acetate (*p* < 0.001). Isovalerate/2-methylbutyrate and isobutyrate were positively correlated with each other (*p* < 0.001), which was not unexpected, as these SCFAs are all produced from the fermentation of branched-chain amino acids.

Strongly inter-correlated bacteria were detected in the liquid fraction. *Oscillospira* had positive correlations with *Moryella* (*r* = 0.55, *p* = 0.0005) and *BF311* (*r* = 0.53, *p* = 0.001) and a negative correlation with *Lachnospira* (*r* = −0.49, *p* = 0.002). *Mogibacterium* was strongly positively correlated with *SHD-231* (*r* = 0.78, *p* <.00001) and *Desulfovibrio* (*r* = 0.71, *p* <.00001). *Schwartzia* and *Selenomonas* were also strongly positively correlated (*r* = 0.76, *p* <.00001). The most abundant genera in liquid, *Prevotella*, was strongly negatively correlated with *Treponema* and *Clostridium*. *Treponema* had a strong positive correlation with *RFN20*. The genera *Shuttleworthia*, *Asteroleplasma*, *Pyramidobacter*, *Treponema*, and *Prevotella*-like bacteria were positively and strongly correlated with each other (*p* < 0.0001; **Figure 4**). *Asteroleplasma*, *Pyramidobacter*, and *Treponema* were positively and strongly correlated with *RFN20* and *Lachnospira* (*p* < 0.0001; **Figure 4**).

In the solid fraction, the genera *Shuttleworthia*, *Coprococcus*, *Selenomonas*, *Dialister*, and *Bulleidia* had strong negative correlations with pH and acetate molar proportion and strong positive correlations with propionate molar proportion and total SCFA concentration (*p* < 0.001). However, *Mogibacterium*, *YRC22*, *CF231*, *Anaerovibrio*, *Clostridium*, *SHD-231*, *BF311, Oscillospira*, *Moryella*, *Desulfovibrio*, *Mogibacter*, *p75-a5* (*p* < 0.001), and *Ruminobacter* (*p* < 0.05) had positive correlations with acetate molar percentage and pH and negative correlations with propionate molar proportion. In addition, *Fibrobacter* was positively correlated with molar proportion of propionate (*p* < 0.05).

In the solid fraction, there was a positive correlation between valerate molar proportion and the genera *Coprococccus*, *Succiniclasticum*, *Shuttleworthia*, and *Bulleidia* (*p* < 0.01); these genera were also positively correlated with propionate. Three of these four genera also had negative correlations with acetate and pH, but *Succiniclasticum* did not (*p* < 0.01). Valerate molar proportion had negative correlations with *YRC22*, *CF231*, *Anaerovibrio*, *Clostridium*, *BF311* (*p* < 0.05), *Oscillospira*, *Moryella*, and *Anaerostipes* (*p* < 0.01). These genera also showed negative correlations with propionate and positive correlations with acetate and pH, except for *Anaerostipes*.

*Butyrivibrio* was positively correlated with the molar proportion of lactate, DMI, and TAA concentration (*p* < 0.01) in the solid fraction. *Shuttleworthia* was positively correlated with the molar proportions of butyrate, valerate (*p* < 0.01), propionate, DMI, ammonia concentration, and total organic acid, and negatively correlated to acetate and pH (*p* < 0.001) in the solid fraction.

In the solid fraction, the genus *Treponema* was positively correlated with isovalerate concentration (*r* = 0.35, *p* = 0.05). *Coprococcus* and *Bulleidia* were also positively correlated with butyrate molar proportion (see **Figure 5** for correlation of solids; correlation *p*-value tables are given in **Supplemental Datasheet 5**).

## 4 Discussion

### 4.1 Sucrose vs. starch: water-soluble vs. insoluble carbohydrate

Before delving into a discussion of the results, we wish to address the constraints and assumptions associated with our study design. The use of fistulated cows allowed the collection of both liquid and authentic solid ruminal samples, whereas such samples are challenging or infeasible to obtain *via* alternative esophageal or buccal sampling methods. However, the use of such cows generally restricts the number of animals used in a study to surgically altered animals, which can potentially limit overall statistical power. The use of lactating animals also limits the duration of a study to that allowed by lactation and was the basis for selecting the split-plot Latin square design of 84 days rather than a 6 × 6 Latin square of 168 days; over the longer period, many cows would have completed lactation and failed to complete the study. The chosen design allowed evaluation of the 2 × 3 factorial arrangement of treatments within the constraints of balancing the duration of the experiment with the length of the cows’ lactations. Application of the Latin square design within the split plot strengthened the ability to detect differences among carbohydrate feed treatments by compensating for the small sample size by assessing multiple treatments within each animal. Assigning protein feed treatments to whole plots allowed evaluation of those treatments but with slightly less sensitivity. Despite these potential limitations, the study’s design stands out by characterizing the liquid and solid communities separately, an aspect often overlooked in similar rumen community studies, and detected differences among treatments. Future studies with alternate designs and foci may provide further resolution to the findings in the current study. Regarding our analysis of the microbiome data, we chose to employ a differential abundance testing approach to analyze the data in an effort to be more conservative regarding our conclusions, as this approach is known to be more effective in analyzing the sparse datasets that characterize these types of studies.

Sucrose is a water-soluble sugar that predominates in molasses, whereas starch in corn grain is primarily water-insoluble. In the current study, molasses replaced corn grain in the diet, which decreased the starch and increased the WSC+TSI levels in the diet as molasses increased. An expected result of increasing sucrose would be an increased relative abundance of plant sugar-utilizing microbes in the liquid fraction. However, all of the microbes in the liquid fraction that were affected by carbohydrate source in the diet declined in relative abundance with increasing molasses. In the solid fraction, six of the seven genera affected by carbohydrate source increased with increasing molasses. The basis of these results is unclear. Other factors such as bacteriocins (Piwonka and Firkins, 1996) or changes in the rate of passage could alter the relative abundance of the genera; however, these measures were not assessed in this study.

The relatively consistent effect of dietary plant sugars on milk fat production (butterfat) in lactating dairy cows was addressed by (Penner and Oba, 2009; with sucrose) and (McKain et al., 2010; with glucose, maltose, and cellobiose). One hypothesis to partially explain this response is that microbes utilizing these sugars may biohydrogenate the dietary unsaturated fatty acids to the point where they no longer exist as the unsaturated isomers that cause milk fat depression. However, interestingly, in the present study, the only positive relative abundance responses observed to molasses supplementation were in the solid fraction. We had expected to see positive responses in the liquid fraction because sucrose is the main sugar in molasses and is water-soluble. Ruminal bacterial populations involved in lipolysis or biohydrogenation may be liquid- or solid-associated (Legay-Carmier and Bauchart, 1989).

### 4.2 Substantial shifts in the composition of bacterial phyla associated with dietary changes

Through meta-analysis of all the deposited 16S rRNA sequences in the Ribosomal Database Project (RDP: The RDP 11.1, released in March 2014), substantial shifts in the composition of the bacterial phyla associated with dietary changes, host, and different environmental conditions have been reported by several researchers (Brulc et al., 2009; Pitta et al., 2010; Uyeno et al., 2010; Hess et al., 2011; Pitta et al., 2014). The animals used in these studies were non-lactating (steers and heifers), and although the study by Uyeno et al. (2010) fed 50% concentrate, the other studies used predominantly forage diets and none supplemented with sucrose or other plant sugars. Even so, the predominant bacterial genera in phylum Firmicutes included *Butyrivibrio*, *Acetivibrio*, *Ruminococcus*, and *Succiniclasticum*, which is very similar to the predominant genera in the present study: *Prevotella*, *Succiniclasticum*, *Butyrivibrio*, *Ruminococcus, Coprococcus*, *Clostridium*, *Treponema, YRC22*, and *CF23*. In the present study, 13 genera were affected by carbohydrate source, compared with only six affected by dietary changes in protein degradability. Dietary supplementation with more RDP altered the ruminal BCC and decreased the richness and diversity of bacteria in both the liquid and solid fractions. When Dai et al. (2016) compared solids and liquid, solids had more of a decrease in diversity as ammonia concentrations increased; this was attributed to more RDP supplementation in the diet and the digestion of solids becoming limited by the hydrolysis step when ammonium increased (Dai et al., 2016). In the current study, especially in the solid fraction with its greater fiber content, the fibrolytic microbes *Butyrivibrio*, *Ruminococcus*, *Prevotella*, *Treponema*, and *Lachnospira* (Cornick and Stanton, 2015; Emerson and Weimer, 2017; Tokuda et al., 2018) had notably higher relative abundances (relative abundance of sequence > 1% in at least one animal). Because fibrolytic microbes require ammonia for growth, they would be expected to obtain a specific advantage with increasing ammonia in the rumen if ammonia concentration limited growth.

### 4.3 RDP effect on microbial community

In the liquid fraction of the current study, the relative abundance of *Prevotella* tended to be positively affected by more RDP, and *Coprococcus* tended to decline. *Prevotella* is known to include proteolytic species that could make use of additional RDP (Russell, 2002). In contrast, *Prevotella* in the solid fraction was unaffected by RDP level, but four other genera were affected: *Succiniclasticum* increased with increasing RDP, and *YRC22*, *Pseudobutyrivibrio*, and *Succinivibrio* decreased with increasing RDP. The positive effects of RDP on *Succiniclasticum* contrast with the study of van Gylswyk (1995), who reported negative effects on *Succiniclasticum ruminis* after protein degradation *via* tests performed for casein hydrolysis and production of propionate from threonine. Because information about this genus is scarce in the literature, a basis is lacking for the interpretation of the positive effect on the relative abundance of *Succiniclasticum*.

Among the bacterial genera in the rumen, *Prevotella* (phylum Bacteroidetes) has been reported to be one of the most numerous and metabolically diverse (Avgustin et al., 1997; Petri et al., 2013). In the current study, the most abundant genus in the solid fraction from the medial-ventral part of the rumen was *Prevotella*. This is in agreement with other studies, which found that *Prevotella*-related sequences were predominant in total 16S rRNA gene sequences recovered from the particle-associated ruminal community (Koike et al., 2003). *Prevotella* was also a member of the core microbiome in ruminal fluid in the present study, with a relative abundance of 62% in the liquid fraction and 42% in the solid fraction, which differed from the findings of Lima et al. (2015) and Petri et al. (2013) that showed *Prevotella* spp. in equal proportions in liquid and solid rumen contents. *Prevotella* abundance may have a relationship with degradable protein availability in the rumen fluid; RDP may have stimulated the growth of *Prevotella* more than fibrolytic activity because, in the liquid fraction, there was a tendency for increasing RDP to increase the relative abundance of *Prevotella*. By being highest in the HiRDP diet with 10.5% M, the relative abundance of the genus *Prevotella* was concordant with the most rumen-degradable diet and likely with increased carbohydrate and protein availabilities. Data from another study showed that *Prevotella* species play an important role in dietary protein breakdown in the rumen (Wallace and McKain, 1991; Wallace et al., 1999) and might benefit from the high level of available protein content of the HiRDP diet.

### 4.4 Genera competing for available substrates

The relative abundance of *Prevotella* was negatively correlated with that of *Succiniclasticum*, *Clostridium* (proteolytic; Russell, 2002), *Anaeroplasma*, *RFN20*, *Schwartzia*, *Treponema* (fibrolytic; Russell, 2002), *Pyramidobacter* (proteolytic; Downes et al., 2009), *Asteroloplasma*, *Mogibacterium*, *Oscillospira* (plant sugar-utilizing; Lee et al., 2013), *BF311*, *Desulfovibrio*, *SDH-231*, *Moryella*, and *Ruminococcus* in the liquid fraction. These results suggest that *Prevotella*, the most abundant genus, might have consumed a greater amount of available substrate (fiber and protein sources; Russell, 2002) than the three abundant genera *Treponema*, *Clostridium*, and *Pyramidobacter*, and may limit their growth by competing for available substrate in the liquid fraction.

The relative abundance of *Butyrivibrio* was negatively correlated with that of *Treponema*; this can be interpreted as competition for substrate because both are fiber digesters (Gradel and Dehority, 1972; Russell, 2002) and were two of the most abundant genera in solids in this study.

Another interesting correlation was seen in the negative relationship between the relative abundance of *Succiniclasticum* and both *Selenomonas* (*p* = 0.058) and *Ruminococcus* in solids. This negative correlation between *Succiniclasticum* and *Selenomonas* may indicate that there is competition between these two genera for succinate because *Selenomonas ruminantium* may be responsible for most of this activity in rumina, according to Wolin and Miller (1988).

### 4.5 Intercorrelations between genera and proposal of possible cross-feeding

We note that correlations between individual genera of bacteria indicate possible interdependencies due to the cross-feeding of hydrolysis products (Sawanon et al., 2006), which was independent of the treatments. In the present study, we found correlations among genera that were accompanied by groupings of these same genera based on their correlations to various fermentation products. In the solids, *Succiniclasticum* was negatively correlated with *Ruminococcus*, *Anaerostipes*, *Pseudobutyrivibrio*, *BF311*, *YRC22*, and *CF231*. Of these genera, four of six (*Anaerostipes*, *BF311*, *YRC22*, and *CF231*) were also negatively correlated with the molar proportion of valerate, whereas *Succiniclasticum* was positively correlated with valerate.

In the solid fraction, the genera negatively correlated with valerate molar proportion (*Anaerovibrio*, *Mogibactrium*, *SHD.231*, *p.75.a5*, *BF311*, *CF231*, *Clostridium*, *Moryella*, *Oscillospira*, and *YRC22*) were also negatively correlated with propionate molar proportion and positively correlated with acetate molar proportion. These results suggest that these 10 genera are primarily acetate producers, and do not participate in the production of valerate from propionate *via* the reversed β-oxidation pathway. The same pattern of ruminal responses was seen in liquid as in solids, only with different organisms involved (this can be seen clearly in **Table 7** for the liquid fraction and **Table 8** for the solid fraction).

**Table 7:** Correlation table for liquid fraction, between the relative abundances of genera (relative abundance > 0.1% at least one sample) and rumen fermentation product.

Genus	pH		Ammonia (mM)		TAA (mM)		Lactate (%)		Acetate (%)		Propionate (%)		Butyrate (%)		Valerate (%)	
	*p*	r	*p*	r	*p*	r	*p*	r	*p*	r	*p*	r	*p*	r	*p*	r
*PrevotellaLike*	<0.001	-0.73	0.008	0.43	0.007	0.44	0.083	0.29	<0.001	-0.71	<0.001	0.68	0.023	0.38	0.001	0.51
*Shuttleworthia*	<0.001	-0.87	0.058	0.32					<0.001	-0.71	<0.001	0.81	0.101	0.28	0.034	0.36
*Asteroleplasma*	0.000	-0.57	0.026	0.37	0.006	0.45	0.061	0.31	0.000	-0.60	0.001	0.55	0.067	0.31	0.011	0.42
*Pyramidobacter*	0.002	-0.50	0.003	0.48	0.007	0.44	0.076	0.30	0.001	-0.54	0.009	0.43	0.012	0.42	0.013	0.41
*Treponema*	0.002	-0.51	0.046	0.34	0.076	0.30			0.001	-0.52	0.005	0.46	0.028	0.37	0.001	0.52
*Lachnospira*	0.001	-0.52							0.001	-0.52	0.001	0.53	0.098	0.28	0.004	0.47
*Schwartzia*	0.039	-0.35							0.008	-0.44	0.011	0.42	0.006	0.45	0.001	0.54
*Fibrobacter*			0.004	0.47	0.011	0.42	0.008	0.43	0.035	-0.35						
*Selenomonas*			0.028	0.37					0.050	-0.33			0.001	0.55	0.007	0.44
*Coprococcus*	0.007	-0.44							0.056	-0.32	0.007	0.44				
*Mogibacterium*	0.007	0.44							0.027	0.37	0.068	-0.31	0.046	-0.33		
*Ruminobacter*	0.053	0.33	0.016	-0.40					0.010	0.42	0.006	-0.45			0.019	-0.39
*Anaerovibrio*	0.001	0.52							0.009	0.43	0.001	-0.53				
*BF311*	0.051	0.33							0.008	0.43	0.001	-0.51				
*Oscillospira*	0.016	0.40							0.005	0.46	0.003	-0.49			0.011	-0.42
*CF231*	0.002	0.49							0.005	0.46	0.001	-0.52	0.049	-0.33		

Correlation is shown for *p* < 0.05.

*p, p*-value; r = correlation.

Correlation was shown for *P* < 0.05.

**Table 8:** Correlation table for solid fraction, between the relative abundances of genera (relative abundance > 0.1% at least one sample) and rumen fermentation product.

Genus	pH		Ammonia (mM)		TAA (mM)		Lactate (%)		Acetate (%)		Propionate (%)		Butyrate (%)		Valerate (%)	
	*p*	r	*p*	r	*p*	r	*p*	r	*p*	r	*p*	r	*p*	r	*p*	r
*Shuttleworthia*	<0.001	-0.83	0.045	0.34					<0.001	-0.81	<0.001	0.84	0.008	0.44	0.010	0.43
*Bulleidia*	0.000	-0.60							<0.001	-0.70	<0.001	0.73	0.050	0.33	0.020	0.39
*Coprococcus*	0.000	-0.59							<0.001	-0.67	<0.001	0.65	0.008	0.44	0.022	0.39
*Dialister*	<0.001	-0.62							<0.001	-0.65	<0.001	0.72				
*Schwartzia*	<0.001	-0.63							0.002	-0.51	0.000	0.59				
*Selenomonas*	<0.001	-0.63							0.017	-0.40	0.004	0.48				
*Fibrobacter*									0.079	0.30	0.025	-0.38				
*Ruminobacter*	0.006	0.45	0.016	-0.40					0.027	0.37	0.017	-0.40				
*Mogibacterium*	0.002	0.50							0.008	0.44	0.004	-0.48			0.077	-0.30
*p.75.a5*	<0.001	0.68							0.005	0.47	0.000	-0.59			0.068	-0.31
*SHD.231*	0.000	0.57							0.004	0.47	0.002	-0.50			0.089	-0.29
*YRC22*	0.010	0.43							0.003	0.49	0.000	-0.61			0.021	-0.39
*BF311*	0.013	0.42							0.003	0.49	0.000	-0.59			0.020	-0.39
*Anaerovibrio*	0.004	0.47			0.082	-0.30			0.002	0.50	0.000	-0.56			0.011	-0.43
*CF231*	0.002	0.51							0.002	0.51	<0.001	-0.63			0.033	-0.36
*Desulfovibrio*	<0.001	0.68							0.001	0.53	0.000	-0.60				
*Clostridium*	<0.001	0.77							0.001	0.55	<0.001	-0.69			0.041	-0.35
*Moryella*	0.000	0.59							0.000	0.57	<0.001	-0.67			0.012	-0.42
*Oscillospira*	<0.001	0.65	0.078	-0.30					<0.001	0.62	<0.001	-0.69			0.001	-0.52
*Butyriovibrio*					0.004	0.47	0.005	0.46								

A relation was shown for *p* < 0.05. *p, p*-value; r = correlation.

Correlation was shown for *P* < 0.05.

### 4.6 Dietary effect on rumen fermentation

Increases in the molar proportions of butyrate in the rumen when increased amounts of sucrose are fed are well documented in *in vivo* and *in vitro* studies, and these studies were important in terms of butyrate for two reasons: (1) the increased butyrate could be used in *de novo* fatty acid production for milk fat (Palmquist et al., 1969) and (2) butyrate constitutes 30% of the fatty acids found in the sn-3 position of milk triglycerides (Jensen, 2002). In the present study, there was a group of genera in the liquid phase, including *Schwartzia*, *Treponema*, *Pyramidobacter*, *Selenomonas*, and *Prevotella*-like bacteria, that were positively correlated with butyrate molar proportion, and they were also negatively correlated with acetate molar proportion (*Selenomonas* not shown in **Figure 4**, but *p* = 0.0502). Of these genera, only one, *Schwartzia*, was positively affected by the inclusion of molasses in the diet. The other four were below the relative abundance of 0.1% and were therefore unlikely to have a significant impact on VFA composition. In the solid fraction, three genera were detected that were positively correlated with the molar proportion of butyrate while negatively correlated with acetate molar proportion. These genera, *Coprococcus*, *Shuttleworthia*, and *Bulleidia*, were unaffected by the inclusion of molasses in the diet. Even at the genus level, it was unexpected that there was not a positive correlation between butyrate molar proportion and the microbes positively affected by molasses inclusion. This conflicts with the general finding, including from this study, that increased ruminal molar proportions of butyrate are observed when ruminants are fed sucrose. However, all of these genera behaved similarly in both liquid and solid fractions, with molar proportions of butyrate and acetate being negatively correlated, which might suggest the inter-conversion of acetate to butyrate (Bergman et al., 1965) as an indirect effect of sucrose fermentation. In addition, it is possible that the butyrate molar proportion could be increased by ciliate protozoa during sucrose fermentation (Teixeira et al., 2017) when molasses was included in the diet.

### 4.7 Molasses’ effect on rumen fermentation

In a study (Baurhoo and Mustafa, 2014) similar to ours, three ruminally cannulated lactating Holstein cows were used to determine the effects of dietary treatments on ruminal fermentation with the addition of dried molasses to high-alfalfa silage diets at 0%, 3%, and 6% of dietary DM. The authors reported that the molar proportion of propionate decreased linearly as the level of molasses increased. This conflicts with the present study, in which we found the treatment effect of molasses on propionate molar percentage to be non-significant; however, diets in the present study contained substantial amounts of starch, which could obscure such effects. A higher molar proportion of propionate is expected for cows fed the 0% molasses diet, due to its greater starch content relative to the other (5.25% and 10.5% molasses) diets. In the same study, Baurhoo and Mustafa (2014) found that the molar proportion of acetate increased linearly as the level of dried molasses increased, which again conflicts with the results of the present study because there was an increase in the molar proportion of acetate as molasses level decreased (and corn grain level increased), and the dietary effect of molasses showed a quadratic tendency. These results for molar proportions of acetate and propionate might have been affected by the cumulative effect of dietary inclusion of RDP and molasses and might have been altered in the opposite direction of Baurhoo and Mustafa (2014), but there was no observed dietary interaction with acetate and propionate molar proportions.

In the present study, there was a tendency for a quadratic effect of molasses supplementation on butyrate; less corn grain increased the molar proportion of ruminal butyrate, whereas it tended to decrease the molar proportion of ruminal acetate. This result was strongly correlated with increases in the genera *Coprococcus* and *Shuttleworthia* in the solid fraction, which is consistent with the known role of *Coprococcus* in producing butyrate (Louis and Flint, 2009; Vital et al., 2014) by the butyryl-CoA: acetate CoA-transferase pathway. The butyryl-CoA: acetate CoA-transferase route is far more prevalent in the human gut ecosystem than the butyrate kinase route (Louis et al., 2004). This is in agreement with stable isotope studies that have demonstrated the incorporation of acetate into butyrate (Duncan et al., 2004). For *Coprococcus eutactus* L2-50, only 28% of butyrate was produced from acetate by the butyrate kinase route, whereas for several other strains, the butyryl-CoA: acetate CoA- transferase route was used >85% of the time. Our ruminal *Coprococcus* in high-molasses diets might include several strains that use the butyryl-CoA: acetate CoA-transferase route. In addition, a high level of acetate incorporation was also found in fecal incubations on several substrates, which appears to be consistent with a dominant role for the butyryl-CoA: acetate CoA-transferase route in the mixed community (Duncan et al., 2004). In our study, there is further evidence for this transferase route from the liquid fraction, as members of three different phyla, Firmicutes, Bacteroidetes, and Spirochaetes, showed highly positive correlations with butyrate, but highly negative correlations with acetate. This agrees with the Genomes Study of the Human Microbiome Project, in which 225 bacteria with the potential to produce butyrate were identified, including many unknown candidates. The majority of candidates belong to distinct families within Firmicutes, but there are also members of nine other phyla, especially Bacteroidetes, Proteobacteria, and Spirochaetes. Diversity analysis of the acetyl-CoA pathway showed that the same few Firmicutes groups associated with Lachnospiraceae and Ruminococcaceae dominated in most individuals, whereas the other pathways were associated primarily with Bacteroidetes. Similar results were found in our study, with the following genera strongly correlated with increasing butyrate and decreasing acetate molar proportions in both liquid and solid fractions (**Tables 7**, **8**): within Firmicutes, *Coprococcus*, *Lachnospira*, *Shuttleworthia*, *Schwartzia*, and *Selenomonas*; within Bacteroidetes, *Prevotella* and *CF231*; and within Spirochaetes, *Treponema*.

In conclusion, replacing corn with molasses at different levels of rumen-degradable protein caused significant alterations in the rumen bacterial community in the liquid and solid fractions at all taxonomic levels, presumably by changing the substrate source and availability. Ruminal BCC with molasses supplementation was not clearly separated from ruminal BCC without molasses supplementation in both liquid and solid fractions, or at different levels of RDP. However, the levels of dietary RDP significantly affected the ruminal BCC in the solid but not in the liquid fraction of the ruminal contents. The only evidence for increased proteolytic activity with increased availability of ruminally degradable protein was the increase in the concentration of total amino acids and increased lactic acid molar percentage; ammonia was unaffected. It is possible that the protein sources were used directly for microbial cell growth or products and so would not have appeared as a breakdown product in the rumen fluid. Furthermore, there were no differences observed in some of the most active proteolytic genera, such as *Ruminobacter* and *Butyrivibrio*, across the treatments. *Prevotella* was the most abundant genus across all treatments. Interestingly, *Prevotella* was found to be more abundant in the liquid than the solid fraction, which is also contrary to expectations. Furthermore, some other genera were unexpectedly abundant, such as *Succiniclasticum*, which was second in abundance in both the liquid and solid fractions. Newly identified effects of *Schwartzia* on succinate degradation were confirmed. Negative dietary effects of more degradable protein were seen on many carbohydrate utilizers, such as *Clostridium*, *Succinivibrio*, *Pseudobutyrivibrio*, and *Coprococcus.* Despite substantial differences in BCC induced by different dietary treatments, fermentation profiles displayed only minor differences, consistent with the high level of functional redundancy within the rumen microbial community (Taxis et al., 2015).

## Data availability statement

The datasets presented in this study can be found in online repositories. The names of the repository and accession number are https://www.ncbi.nlm.nih.gov/ and PRJNA953606, respectively.

## Ethics statement

The animal study was reviewed and approved by the University of Wisconsin College of Agriculture and Life Sciences Institutional Animal Care and Use Committee.

## Author contributions

EG and AS performed the sequencing analysis of samples. GZ and MH performed the animal study from which the samples were obtained. EG, KW, GS, PW, GZ, and MH collaborated on the design of the experiment. EG performed the statistical analyses and wrote the manuscript. EG, PW, MH, GS, and GZ reviewed and revised the manuscript for content, interpretation, and form. All authors contributed to the article and approved the submitted version.
