# Supplementary material for: Characterization of rumen microbiota in lactating Holstein cows fed molasses versus corn grain at two levels of rumen-degradable protein
Source: Front Microbiomes. 2023 Aug 15;2:1204988. doi: 10.3389/frmbi.2023.1204988 (PMC12993626; doi:10.3389/frmbi.2023.1204988)
Supplement: Supplementary file 1 [file DataSheet_1.zip › Data sheet 1. Code for Whole Analysis.docx]

# Supplementary Text

**Supplementary Text 1.** Sequence data processing code in mothur:

make.contigs(file=elif.txt,processors=10)

summary.seqs(fasta=elif.trim.contigs.fasta)

screen.seqs(fasta=elif.trim.contigs.fasta,group=elif.contigs.groups,, maxambig=0, maxhomop=8, minlength=200, maxlength=500)

summary.seqs(fasta=elif.trim.contigs.good.fasta)

unique.seqs(fasta=elif.trim.contigs.good.fasta)

count.seqs(name=elif.trim.contigs.good.names,group=elif.contigs.good.groups)

summary.seqs(fasta=elif.trim.contigs.good.unique.fasta,count=elif.trim.contigs.good.count_table)

align.seqs(fasta=elif.trim.contigs.good.unique.fasta, reference= silva.nr_v123.align, flip=T)

summary.seqs(fasta=elif.trim.contigs.good.unique.align,count=elif.trim.contigs.good.count_table)

screen.seqs(fasta=elif.trim.contigs.good.unique.align,count=elif.trim.contigs.good.count_table, summary=elif.trim.contigs.good.unique.summary, start=13862, end=23444)

summary.seqs(fasta=elif.trim.contigs.good.unique.good.align, count=elif.trim.contigs.good.good.count_table)

filter.seqs(fasta=elif.trim.contigs.good.unique.good.align, vertical=T, trump=.)

unique.seqs(fasta=elif.trim.contigs.good.unique.good.filter.fasta, count=elif.trim.contigs.good.good.count_table)

pre.cluster(fasta=elif.trim.contigs.good.unique.good.filter.unique.fasta,count=elif.trim.contigs.good.unique.good.filter.count_table, diffs=2)

summary.seqs(fasta=elif.trim.contigs.good.unique.good.filter.unique.precluster.fasta, count=elif.trim.contigs.good.unique.good.filter.unique.precluster.count_table)

chimera.uchime(fasta=elif.trim.contigs.good.unique.good.filter.unique.precluster.fasta, count=elif.trim.contigs.good.unique.good.filter.unique.precluster.count_table)

remove.seqs(fasta=elif.trim.contigs.good.unique.good.filter.unique.precluster.fasta, count=elif.trim.contigs.good.unique.good.filter.unique.precluster.count_table, accnos=elif.trim.contigs.good.unique.good.filter.unique.precluster.denovo.uchime.accnos)

summary.seqs(fasta=elif.trim.contigs.good.unique.good.filter.unique.precluster.pick.fasta, count=elif.trim.contigs.good.unique.good.filter.unique.precluster.pick.count_table)

classify.seqs(fasta=elif.trim.contigs.good.unique.good.filter.unique.precluster.pick.fasta, count=elif.trim.contigs.good.unique.good.filter.unique.precluster.uchime.pick.count_table,reference= silva.nr_v123.align, taxonomy= silva.nr_v123.tax, cutoff=80)

remove.lineage(fasta=elif.trim.contigs.good.unique.good.filter.unique.precluster.pick.fasta, count=elif.trim.contigs.good.unique.good.filter.unique.precluster.pick.count_table, taxonomy=elif.trim.contigs.good.unique.good.filter.unique.precluster.pick.nr_v123.wang.taxonomy,taxon=unknown;-Archaea;-Eukaryota;-Bacteria;Cyanobacteria;-Bacteria;Proteobacteria;Alphaproteobacteria;Rickettsiales;mitochondria;)

summary.seqs(fasta=elif.trim.contigs.good.unique.good.filter.unique.precluster.pick.pick.fasta, count=elif.trim.contigs.good.unique.good.filter.unique.precluster.pick.pick.count_table)

count.groups(count=elif.trim.contigs.good.unique.good.filter.unique.precluster.pick.pick.count_table)

split.abund(fasta=elif.trim.contigs.good.unique.good.filter.unique.precluster.pick.pick.fasta, count=elif.trim.contigs.good.unique.good.filter.unique.precluster.pick.pick.count_table, cutoff=1)

count.groups(count=elif.trim.contigs.good.unique.good.filter.unique.precluster.pick.pick.abund.count_table)

summary.seqs(fasta=elif.trim.contigs.good.unique.good.filter.unique.precluster.pick.pick.abund.fasta, count=elif.trim.contigs.good.unique.good.filter.unique.precluster.pick.pick.abund.count_table)

system(cp elif.trim.contigs.good.unique.good.filter.unique.precluster.pick.pick.abund.fasta elif.final.fasta)

system (cp elif.trim.contigs.good.unique.good.filter.unique.precluster.pick.pick.abund.count_table elif.final.count_table)

dist.seqs(fasta=elif.final.fasta)

cluster.split(column=elif.final.dist, count=elif.final.count_table, method=average, cutoff=0.1)

make.shared(list=elif.final.an.unique_list.list, count=elif.final.count_table, label=0.03)

normalize.shared(shared=elif.final.an.unique_list.shared, norm=18,286)

summary.single(shared=elif.final.an.unique_list.0.03.norm.shared, label=0.03, calc=nseqs-sobs-coverage-ace-chao-shannon-simpson-invsimpson)

classify.seqs(fasta=elif.final.fasta,count=elif.final.count_table, template=gg_13_8_99.fasta,taxonomy=gg_13_8_99.gg.tax, cutoff=80)

classify.otu(list=elif.final.an.unique_list.list,taxonomy=elif.final.gg.wang.taxonomy,count=elif.final.count_table,label=0.03, cutoff=80, basis=otu, probs=F)

get.oturep(column=elif.final.dist,list=elif.final.an.unique_list.list,count=elif.final.count_table,fasta=elif.final.fasta,label=0.03, large=true)

**Supplementary Text 2**. Code-1 for Microbiota Analysis in R.

source("https://bioconductor.org/biocLite.R")

biocLite("phyloseq")

source("https://bioconductor.org/biocLite.R")

biocLite("DESeq2")

install.packages("extrafont")#exporting figures

library(extrafont)

library(ape)

library(DESeq2)

library(dplyr)

library(ggplot2)

library(gplots)

library(lme4)

library(miLineage)

library(phangorn)

library(phyloseq)

library(plotly)

library(tidyr)

library(vegan)

library(VennDiagram)

library(venneuler)

library(phyloseq)

PREPARING THE DATA

OTU = read.table("elif.final.abund.an.unique_list.0.03.norm.shared",header = TRUE, sep="\t")

tax = read.table("elif.final.abund.an.unique_list.0.03.cons.taxonomy",header = TRUE, sep="\t")

diversity = read.table("elif.final.abund.an.unique_list.0.03.norm.groups.summary",header = TRUE, sep="\t")

meta = read.table("elif.metadata.txt", header=TRUE, sep="\t")

SCFA = read.table("elif.SCFA.txt", header=TRUE, sep="\t")

row.names(SCFA)<-SCFA$group

row.names(meta)<-meta$group

SUBSETTING DATA: subset data by sample type

meta.L <- meta[which(meta$sample.type=="Liquid"),]

meta.S <- meta[which(meta$sample.type=="Solid"),]

meta.M <- meta[which(meta$RDP=="M"),]

meta.LS <- meta[which(meta$RDP=="L"),]

meta.1L <- meta[which((meta$molas=="0%") & (meta$sample.type=="Liquid")),]

meta.2L <- meta[which((meta$molas=="5.25%")& (meta$sample.type=="Liquid")),]

meta.3L <- meta[which((meta$molas=="10.5%")& (meta$sample.type=="Liquid")),]

meta.1 <- meta[which(meta$molas=="0%"),]

meta.2 <- meta[which(meta$molas=="5.25%"),]

meta.3 <- meta[which(meta$molas=="10.5%"),]

OTU.clean.L <- OTU.clean[which(meta$sample.type=="Liquid"),]

OTU.clean.S <- OTU.clean[which(meta$sample.type=="Solid"),]

OTU.clean.M <- OTU.clean[which(meta$RDP=="M"),]

OTU.clean.LS <- OTU.clean[which(meta$RDP=="L"),]

OTU.RDP <- rbind(OTU.clean.M, OTU.clean.LS )

OTU.clean.1 <- OTU.clean[which(meta$molas=="0%"),]

OTU.clean.2 <- OTU.clean[which(meta$molas=="5.25%"),]

OTU.clean.3 <- OTU.clean[which(meta$molas=="10.5%"),]

tax.clean.L <- tax.clean[which(meta$sample.type=="Liquid"),]

tax.clean.S <- tax.clean[which(meta$sample.type=="Solid"),]

SUBSETTING DATA: to a group of samples to the same samples in another file

#Use the value-matching function %in%. The names are a little different between the #tables so we also add “.F” to the SCFA names to make them match.

#OTU.SCFA = OTU.clean[row.names(OTU.clean) %in% paste(row.names(SCFA), ".F", #sep=""),]

#meta.SCFA = meta[row.names(meta) %in% paste(row.names(SCFA), ".F", sep=""),]

OTU.SCFA = OTU.clean[row.names(OTU.clean) %in% row.names(SCFA),]

meta.SCFA = meta[row.names(meta) %in% row.names(SCFA),]

Remove rare taxa

OTU.clean.abund=OTU.clean[,which(apply(OTU.clean,2,max)>10)]

Number of columns before

ncol(OTU.clean) #3210

Number of columns after

ncol(OTU.clean.abund) #713

Convert OTU counts to proportions: In short, we want to divide all cells in a row by #the total of that row. We can accomplish this using the sweep function. We want to #sweep all rows (1) of the OTU.clean data frame, dividing (/) each value by the #rowSum.

OTU.clean.relabund <- sweep(OTU.clean,1,rowSums(OTU.clean),"/")

rowSums(OTU.clean)

rowSums(OTU.clean.relabund)

Set seed

We will be running some processes that rely on the random number generater. To make #your analysis reproducible, we set the random seed.

set.seed(8765)

#ALPHA DIVERSITY

#create a phyloseq object

OTU.physeq = otu_table(as.matrix(OTU.clean), taxa_are_rows=FALSE)

OTU.physeq.L = otu_table(as.matrix(OTU.clean.L), taxa_are_rows=FALSE) # LIQUIDS

OTU.physeq.S = otu_table(as.matrix(OTU.clean.S), taxa_are_rows=FALSE) # SOLIDS

tax.physeq = tax_table(as.matrix(tax.clean))

tax.physeq.L = tax_table(as.matrix(tax.clean.L))

tax.physeq.S = tax_table(as.matrix(tax.clean.S))

meta.physeq = sample_data(meta)

meta.physeq.L = sample_data(meta.L)

meta.physeq.S = sample_data(meta.S)

physeq.alpha = phyloseq(OTU.physeq, tax.physeq, meta.physeq)

physeq.alpha.L = phyloseq(OTU.physeq.L, tax.physeq.L, meta.physeq.L)

physeq.alpha.S = phyloseq(OTU.physeq.S, tax.physeq.S, meta.physeq.S)

#generate alpha diversity column

sample_data(physeq.alpha)$shannon.physeq <- estimate_richness(physeq.alpha, measures="Shannon")

sample_data(physeq.alpha.L)$shannon.physeq <- estimate_richness(physeq.alpha.L, measures="Shannon")

sample_data(physeq.alpha.S)$shannon.physeq <- estimate_richness(physeq.alpha.S, measures="Shannon")

# phyloseq also allows you to easily plot alpha diversity, both by sample and by group.

pdf("Fig2.pdf", width = 7, height = 3, colormodel = "rgb", family = "ArialMT")

#layout(matrix(c(1,1,2,3), 2, 2, byrow = TRUE)))

#plot_richness(physeq.alpha, measures="Shannon") #PLOT1.1

#par(mfrow=c(1,3))

plot_richness(physeq.alpha.L, measures="Shannon") #PLOT1.2

dev.off()

pdf("Fig3.pdf", width = 7, height = 3, colormodel = "rgb", family = "ArialMT")

plot_richness(physeq.alpha.S, measures="Shannon") #PLOT1.3

dev.off()

plot_richness(physeq.alpha, "sample.type", measures="Shannon") #PLOT2

dev.off()

plot_richness(physeq.alpha, "diets", measures="Shannon") #PLOT3

plot_richness(physeq.alpha, "RDP", measures="Shannon") #PLOT3.1

plot_richness(physeq.alpha, "Period", measures="Shannon") #PLOT3.2

plot_richness(physeq.alpha, "molas", measures="Shannon") #PLOT3.3

#vegan calculations

meta$shannon.vegan <- diversity(OTU.clean, index="shannon")

#Compare methods

#Now, lets plot these three versions of shannon vs. sample index

#view and compare to mothur calculated Shannon Index #PLOT4: Suffice to say, they are #very similar.

#par(mfrow=c(3,1))

pdf("Fig1.pdf", width = 7, height = 6, colormodel = "rgb", family = "ArialMT")

layout(matrix(c(1,2,3), 3, 1), widths=c(1,1), heights=c(1,1))

plot(x=c(1:nsamples(physeq.alpha)), y=meta$shannon, main="Shannon diversity (mothur)", xlab='Samples', ylab='Alpha Diversity Measure')

plot(x=c(1:nsamples(physeq.alpha)), y=sample_data(physeq.alpha)$shannon.physeq$Shannon, main="Shannon diversity (phyloseq)", xlab='Samples',ylab='Alpha Diversity Measure')

plot(x=c(1:nsamples(physeq.alpha)), y=meta$shannon.vegan, main="Shannon diversity (vegan)", xlab='Samples', ylab='Alpha Diversity Measure')

#Alpha statistics: alpha-diversity and richness plots

#Create 2x2 plot environment so that we can see all 4 metrics at once.

par(mfrow = c(2, 2))

#Then plot each metric. FIGURE 1

hist(meta$shannon, main="Shannon diversity", xlab="", breaks=10)

hist(meta$simpson, main="Simpson diversity", xlab="", breaks=10)

hist(meta$chao, main="Chao richness", xlab="", breaks=15)

hist(meta$ace, main="ACE richness", xlab="", breaks=15)

#You want the data to be roughly normal so that you can run ANOVA or t-tests. If it #is not normally distributed, you will need to consider non-parametric tests such as #Kruskal-Wallis.

#Here, we see that none of the data are normally distributed. This occurs with the #subset but not the full data set because I’ve specifically selected samples with #divergent alpha metrics. In general, you will see roughly normal data for Shannon’s #diversity as well as most richness metrics. Simpson’s diversity, on the other hand, #is usually skewed as seen here.

#So most will use inverse Simpson (1/Simpson) instead. This not only increases #normalcy but also makes the output more logical as a higher inverse Simpson value #corresponds to higher diversity.

#Let’s look at inverse Simpson instead.

#Create 2x2 plot environment

par(mfrow = c(2, 2))

#Plots FIGURE 2

hist(meta$shannon, main="Shannon diversity", xlab="", breaks=10)

hist(1/meta$simpson, main="Inverse Simpson diversity", xlab="", breaks=10)

hist(meta$chao, main="Chao richness", xlab="", breaks=15)

hist(meta$ace, main="ACE richness", xlab="", breaks=15)

#Now we see a bimodal distribution for Simpson similar to the richness metrics.

#To test for normalcy statistically, we can run the Shapiro-Wilk test of normality.

shapiro.test(meta$shannon)

shapiro.test(1/meta$simpson)

shapiro.test(meta$chao)

# shapiro.test(meta$shannon)

# Shapiro-Wilk normality test

# data: meta$shannon

# W = 0.97421, p-value = 0.1514

# > shapiro.test(1/meta$simpson)

# Shapiro-Wilk normality test

# data: 1/meta$simpson

# W = 0.88764, p-value = 1.149e-05

# > shapiro.test(meta$chao)

# Shapiro-Wilk normality test

# data: meta$chao

# W = 0.95653, p-value = 0.01511

# > shapiro.test(meta$ace)

# Shapiro-Wilk normality test

# data: meta$ace

# W = 0.93769, p-value = 0.001585

#COMMENTS: none are normaL: run statistical tests that don’t assume our data is normal, because we don’t have any evidence (graphs, Shapiro-Wilk) that it is normal. Theoretically. In reality, if your data is roughly hill-shaped, not heavily skewed, and not bimodal, most tests are robust enough to handle it. But, when in doubt, use a test that does not assume normality (nonparametric). None of these metrics are normal-looking enough that I would use a parametric test. However, we are going to show both parametric and nonparametric examples for demonstration purposes.

# Overall, for alpha-diversity:

# ANOVA, t-test, or general linear models with the normal distribution are used when the data is roughly normal

# Kruskal-Wallis, Wilcoxon rank sum test, or general linear models with another distribution are used when the data is not normal

# Our main variables of interest are

# Categorical: SAMPLE.TYPE: (L,S) AND DIETS(A,B,C,A-,B-,C-)

# Continuous: NONE

# normally distributed metrics

# Since it’s the closest to normalcy, we will use Shannon’s diversity as an example. First, we will test age, which is a categorical variable with more than 2 levels. Thus, we run ANOVA. If age were only two levels, we could run a t-test

# Does SAMPLE TYPE impact the Shannon diversity of the fecal microbiota?

#ANOVA FOR SAMPLE TYPE

#Run the ANOVA and save it as an object

aov.shannon.sample = aov(shannon ~ sample.type, data=meta)

#Call for the summary of that ANOVA, which will include P-values

summary(aov.shannon.sample)

# Df Sum Sq Mean Sq F value Pr(>F)

# sample.type 1 2.303 2.3033 27.62 1.56e-06 ***

# Residuals 69 5.753 0.0834

# ---

# Signif. codes: 0 ‘***’ 0.001 ‘**’ 0.01 ‘*’ 0.05 ‘.’ 0.1 ‘ ’ 1

# To do all the pairwise comparisons between groups and correct for multiple comparisons, we run Tukey’s honest significance test of our ANOVA.

TukeyHSD(aov.shannon.sample)

# Tukey multiple comparisons of means

# 95% family-wise confidence level

# Fit: aov(formula = shannon ~ sample.type, data = meta)

# $sample.type

# diff lwr upr p adj

# Solid-Liquid 0.360262 0.223521 0.497003 1.6e-06

# We clearly see that all sample groups have significantly different diversity. When we plot the data, we see that diversity increases as the solids.

#Re-order the groups

meta$sample.type.ord = factor(meta$sample.type, c("Liquid","Solid"))

#Return the plot area to 1x1

par(mfrow = c(1, 1)) #PLOT 5

#Plot

boxplot(shannon ~ sample.type.ord, data=meta, ylab="Shannon's diversity")

#ANOVA FOR DIETS

#Does DIETS impact the Shannon diversity of the fecal microbiota?

aov.shannon.diets = aov(shannon ~ diets, data=meta)

summary(aov.shannon.diets)

# Df Sum Sq Mean Sq F value Pr(>F)

# diets 5 0.686 0.1373 1.211 0.314

# Residuals 65 7.370 0.1134

TukeyHSD(aov.shannon.diets)

# Tukey multiple comparisons of means

# 95% family-wise confidence level

# Fit: aov(formula = shannon ~ diets, data = meta)

# $diets

# diff lwr upr p adj

# A (-)-A 0.26028649 -0.1524462 0.6730192 0.4407075

# B-A -0.02804726 -0.4407800 0.3846855 0.9999542

# B (-)-A 0.16298741 -0.2497453 0.5757201 0.8540839

# C-A 0.05476724 -0.3579655 0.4675000 0.9987840

# C (-)-A 0.11031566 -0.3024171 0.5230484 0.9691547

# B-A (-) -0.28833375 -0.6919943 0.1153268 0.3015097

# B (-)-A (-) -0.09729908 -0.5009596 0.3063615 0.9803590

# C-A (-) -0.20551925 -0.6091798 0.1981413 0.6685902

# C (-)-A (-) -0.14997083 -0.5536314 0.2536897 0.8832532

# B (-)-B 0.19103467 -0.2126259 0.5946952 0.7329804

# C-B 0.08281450 -0.3208461 0.4864751 0.9905028

# C (-)-B 0.13836292 -0.2652976 0.5420235 0.9141391

# C-B (-) -0.10822017 -0.5118807 0.2954404 0.9687475

# C (-)-B (-) -0.05267175 -0.4563323 0.3509888 0.9988786

# C (-)-C 0.05554842 -0.3481121 0.4592090 0.9985511

meta$diets.ord = factor(meta$diets)

#Return the plot area to 1x1

par(mfrow = c(1, 1)) #PLOT 6

#ANOVA FOR RDP

#Does RDP impact the Shannon diversity of the fecal microbiota?

aov.shannon.RDP = aov(shannon ~ RDP, data=meta)

summary(aov.shannon.RDP)

# Df Sum Sq Mean Sq F value Pr(>F)

# RDP 1 0.505 0.5051 4.615 0.0352 *

# Residuals 69 7.551 0.1094

# ---

# Signif. codes: 0 ‘***’ 0.001 ‘**’ 0.01 ‘*’ 0.05 ‘.’ 0.1 ‘ ’ 1

TukeyHSD(aov.shannon.RDP)

# Tukey multiple comparisons of means

# 95% family-wise confidence level

# Fit: aov(formula = shannon ~ RDP, data = meta)

# $RDP

# diff lwr upr p adj

# M-L -0.168702 -0.3253627 -0.01204139 0.035204

# meta$diets.ord = factor(meta$diets)

# #Return the plot area to 1x1

# par(mfrow = c(1, 1)) #PLOT 6

# #Plot

boxplot(shannon ~ RDP, data=meta, ylab="Shannon's diversity")

#ANOVA FOR MOLASSES LEVEL

#Does MOLASSES LEVEL impact the Shannon diversity of the fecal microbiota?

aov.shannon.molas = aov(shannon ~ molas, data=meta)

summary(aov.shannon.molas)

# # Df Sum Sq Mean Sq F value Pr(>F)

# molas 2 0.060 0.0301 0.256 0.775

# Residuals 68 7.996 0.1176

TukeyHSD(aov.shannon.molas)

# Tukey multiple comparisons of means

# 95% family-wise confidence level

# Fit: aov(formula = shannon ~ molas, data = meta)

# $molas

# diff lwr upr p adj

# 10.5%-0% -0.05326020 -0.2930148 0.1864944 0.8557737

# 5.25%-0% -0.06833157 -0.3080862 0.1714231 0.7742269

# 5.25%-10.5% -0.01507138 -0.2522617 0.2221190 0.9873044

boxplot(shannon ~ molas, data=meta, ylab="Shannon's diversity")

DTK.result<-DTK.test(x=meta$shannon, f=meta$molas,a=0.05)

DTK.plot(DTK.result)

DTK.result<-DTK.test(x=meta.L$shannon, f=meta.L$molas, a=0.05)

TK.result<-TK.test(x=meta$shannon, f=meta$molas, a=0.05)

DTK.result<-DTK.test(x=meta$shannon, f=meta$diets,a=0.05)

DTK.plot(DTK.result)

DTK.result<-DTK.test(x=meta.L$shannon, f=meta.L$diets, a=0.05)

TK.result<-TK.test(x=meta.L$shannon, f=meta.L$diets, a=0.05)

TK.result<-TK.test(x=x,f=f,a=0.05)

DTK.result

TK.result

TK.result<-TK.test(x=meta$shannon, f=meta$Period,a=0.05)

> TK.result

# Tukey multiple comparisons of means

# 95% family-wise confidence level

# Fit: aov(formula = x ~ f)

# $f

# diff lwr upr p adj

# P2-P1 0.08602754 -0.1497976 0.3218527 0.6583907

# P3-P1 0.10675268 -0.1316220 0.3451274 0.5339466

# P3-P2 0.02072514 -0.2176495 0.2590998 0.9763673

#Return the plot area to 1x1

par(mfrow = c(1, 1)) #PLOT 8

#Plot

boxplot(shannon ~ RDP, data=meta, ylab="Shannon's diversity")

#PLOT 7 WITH SUBSAMPLED DATA BY SAMPLE TYPE

par(mfrow=c(1,2))

boxplot(shannon ~ diets, data=meta.L, xlab="Liquid", ylab="Shannon's diversity")

boxplot(shannon ~ diets, data=meta.S, xlab="Solid", ylab="Shannon's diversity")

#Non-normally distributed metrics: chao

# Since our variables are categorical (sample type, diets and RDP levels, molasses) ; Kruskal-Wallis (non-parametric equivalent of ANOVA). If we have only two levels, we would run Wilcoxon rank sum # test (non-parametric equivalent of t-test)

#K.W. takes only one group at a time.

kruskal.test(chao ~ diets, data=meta)

kruskal.test(chao ~ sample.type, data=meta)

kruskal.test(chao ~ Period, data=meta)

kruskal.test(chao ~ RDP, data=meta)

kruskal.test(chao ~ molas, data=meta)

# Kruskal-Wallis rank sum test

# data: chao by diets

# Kruskal-Wallis chi-squared = 7.3185, df = 5, p-value = 0.198

pairwise.wilcox.test(meta$chao, meta$diets, p.adjust.method="fdr")

# Pairwise comparisons using Wilcoxon rank sum test

# data: meta$chao and meta$diets

# A A (-) B B (-) C

# A (-) 0.58 - - - -

# B 0.58 0.29 - - -

# B (-) 0.85 0.98 0.57 - -

# C 0.85 0.58 0.77 0.58 -

# C (-) 0.58 0.86 0.26 0.80 0.58

# P value adjustment method: fdr

#DIETS?

#Like diversity (p=0.314), we see that richness also not changes enough with diets p-value = 0.198 with Kruskal-Wallis rank sum test.

#Create 1x1 plot environment

#par(mfrow = c(1, 1)) ##PLOT10

#Plot

boxplot(chao ~ diets, data=meta, ylab="Chao richness")

#Plot

pdf("Fig4.pdf", width = 7, height = 8, colormodel = "rgb", family = "ArialMT")

#layout(matrix(c(1,2,3,4), 2, 2, byrow = TRUE), widths=c(1,1), heights=c(1,1))

par(mfrow = c(2, 2), # 2x2 layout

# oma = c(2, 2, 2, 2), # two rows of text at the outer left and bottom margin

mar = c(3, 3, 3, 3), # space for one row of text at ticks and to separate plots

mgp = c(2, 1, 0), # axis label at 2 rows distance, tick labels at 1 row

xpd = NA) # allow content to protrude into outer margin (and beyond)

boxplot(shannon ~ diets, data=meta.L, ylab="Shannon's diversity", main="Liquids")

boxplot(shannon ~ diets, data=meta.S, ylab="Shannon's diversity", main="Solids")

boxplot(chao ~ diets, data=meta.L, ylab="Chao richness")

boxplot(chao ~ diets, data=meta.S, ylab="Chao richness")

dev.off()

#Plot for RDP

pdf("Fig7.pdf", width = 7, height = 8, colormodel = "rgb", family = "ArialMT")

#layout(matrix(c(1,2,3,4), 2, 2, byrow = TRUE), widths=c(1,1), heights=c(1,1))

par(mfrow = c(2, 2), # 2x2 layout

# oma = c(2, 2, 2, 2), # two rows of text at the outer left and bottom margin

mar = c(3, 3, 3, 3), # space for one row of text at ticks and to separate plots

mgp = c(2, 1, 0), # axis label at 2 rows distance, tick labels at 1 row

xpd = NA) # allow content to protrude into outer margin (and beyond)

boxplot(shannon ~ RDP, data=meta.L, ylab="Shannon's diversity", main="Liquids")

boxplot(shannon ~ RDP, data=meta.S, ylab="Shannon's diversity", main="Solids")

boxplot(chao ~ RDP, data=meta.L, ylab="Chao richness")

boxplot(chao ~ RDP, data=meta.S, ylab="Chao richness")

dev.off()

#Plot for MOLASSES

pdf("Fig8.pdf", width = 7, height = 8, colormodel = "rgb", family = "ArialMT")

#layout(matrix(c(1,2,3,4), 2, 2, byrow = TRUE), widths=c(1,1), heights=c(1,1))

par(mfrow = c(2, 2), # 2x2 layout

# oma = c(2, 2, 2, 2), # two rows of text at the outer left and bottom margin

mar = c(3, 3, 3, 3), # space for one row of text at ticks and to separate plots

mgp = c(2, 1, 0), # axis label at 2 rows distance, tick labels at 1 row

xpd = NA) # allow content to protrude into outer margin (and beyond)

boxplot(shannon ~ molas, data=meta.L, ylab="Shannon's diversity", main="Liquids")

boxplot(shannon ~ molas, data=meta.S, ylab="Shannon's diversity", main="Solids")

boxplot(chao ~ molas, data=meta.L, ylab="Chao richness")

boxplot(chao ~ molas, data=meta.S, ylab="Chao richness")

dev.off()

#MOLASSES?

#Like diversity (p=0.775) anova, we see that richness also not changes correlated by the molasses level increases p-value = 0.5131, with Kruskal-Wallis rank sum test.

kruskal.test(chao ~ molas, data=meta)

# # Kruskal-Wallis rank sum test

# data: chao by molas

# Kruskal-Wallis chi-squared = 1.3347, df = 2, p-value = 0.5131

boxplot(chao ~ molas, data=meta, ylab="Chao richness") #PLOT12

#RDP LEVEL? #Like diversity (p= 0.0352) anova, we see that richness also increase by the less RDP p-value = 0.017, comparisons using Wilcoxon rank sum test.

pairwise.wilcox.test(meta$chao, meta$RDP, p.adjust.method="fdr")

# Pairwise comparisons using Wilcoxon rank sum test

# data: meta$chao and meta$RDP

# L

# M 0.017

# P value adjustment method: fdr

boxplot(chao ~ RDP, data=meta, ylab="Chao richness")

#SAMPLE TYPE?

#unLike diversity increases as the solids (p= 1.6e-06) anova, we see that richness wasn't affected by the sample size p-value = 0.38, comparisons using Wilcoxon rank sum test.

pairwise.wilcox.test(meta$chao, meta$sample.type, p.adjust.method="fdr")

# Pairwise comparisons using Wilcoxon rank sum test

# data: meta$chao and meta$sample.type

# Liquid

# Solid 0.38

# P value adjustment method: fdr

#Mixed models: RDP pvalue=0.013 * and sample.type pvalue=8.78e-07 have #impact on Shannon diversity value.

aov.shannon.all = aov(shannon ~ RDP*sample.type*molas*Period, data=meta)

aov.shannon.all.L = aov(shannon ~ RDP*molas*Period, data=meta.L)

aov.shannon.all.S = aov(shannon ~ RDP*molas*Period, data=meta.S)

aov.shannon.all = aov(shannon ~ RDP*sample.type*molas, data=meta)

summary(aov.shannon.all)

# Df Sum Sq Mean Sq F value

# Df Sum Sq Mean Sq F value Pr(>F)

# RDP 1 0.505 0.5051 6.219 0.0155 *

# sample.type 1 2.273 2.2730 27.987 1.88e-06 ***

# molas 2 0.069 0.0343 0.422 0.6578

# RDP:sample.type 1 0.007 0.0066 0.081 0.7768

# RDP:molas 2 0.112 0.0562 0.693 0.5043

# sample.type:molas 2 0.252 0.1258 1.548 0.2211

# RDP:sample.type:molas 2 0.047 0.0236 0.291 0.7487

# Residuals 59 4.792 0.0812

# ---

# Signif. codes: 0 ‘***’ 0.001 ‘**’ 0.01 ‘*’ 0.05 ‘.’ 0.1 ‘ ’ 1

TukeyHSD(aov.shannon.all)

# Tukey multiple comparisons of means

# 95% family-wise confidence level

# Fit: aov(formula = shannon ~ RDP * sample.type * molas, data = meta)

# $RDP

# diff lwr upr p adj

# M-L -0.168702 -0.3040699 -0.03333421 0.0154635

# $sample.type

# diff lwr upr p adj

# Solid-Liquid 0.357852 0.2224841 0.4932198 1.9e-06

0.0710742

# M:Solid:10.5%-M:Liquid:0% 0.45858483 -0.101233997 1.018403663 0.2123298

# L:Liquid:5.25%-M:Liquid:0% 0.26320000 -0.296618830 0.823018830 0.9023439

# M:Liquid:5.25%-M:Liquid:0% 0.14908417 -0.410734663 0.708902997 0.9988128

# L:Solid:5.25%-M:Liquid:0% 0.59024633 0.030427503 1.150065163 0.0302132

# M:Solid:5.25%-M:Liquid:0% 0.32229283 -0.237525997 0.882111663 0.7179831

# M:Solid:0%-L:Solid:0% -0.18412983 -0.771272776 0.403013109 0.9950067

# L:Liquid:10.5%-L:Solid:0% -0.55369767 -1.113516497 0.006121163 0.0551416

# M:Liquid:10.5%-L:Solid:0% -0.58592733 -1.145746163 -0.026108503 0.0325072

# L:Solid:10.5%-L:Solid:0% -0.22689650 -0.786715330 0.332922330 0.9631182

# M:Solid:10.5%-L:Solid:0% -0.30576367 -0.865582497 0.254055163 0.7789325

# L:Liquid:5.25%-L:Solid:0% -0.50114850 -1.060967330 0.058670330 0.1212080

# M:Liquid:5.25%-L:Solid:0% -0.61526433 -1.175083163 -0.055445503 0.0195720

# L:Solid:5.25%-L:Solid:0% -0.17410217 -0.733920997 0.385716663 0.9953473

# M:Solid:5.25%-L:Solid:0% -0.44205567 -1.001874497 0.117763163 0.2585131

# L:Liquid:10.5%-M:Solid:0% -0.36956783 -0.956710776 0.217575109 0.5963251

# M:Liquid:10.5%-M:Solid:0% -0.40179750 -0.988940442 0.185345442 0.4691340

# L:Solid:10.5%-M:Solid:0% -0.04276667 -0.629909609 0.544376276 1.0000000

# M:Solid:10.5%-M:Solid:0% -0.12163383 -0.708776776 0.465509109 0.9998902

# L:Liquid:5.25%-M:Solid:0% -0.31701867 -0.904161609 0.270124276 0.7910371

# M:Liquid:5.25%-M:Solid:0% -0.43113450 -1.018277442 0.156008442 0.3612573

# L:Solid:5.25%-M:Solid:0% 0.01002767 -0.577115276 0.597170609 1.0000000

# M:Solid:5.25%-M:Solid:0% -0.25792583 -0.845068776 0.329217109 0.9362493

# M:Liquid:10.5%-L:Liquid:10.5% -0.03222967 -0.592048497 0.527589163 1.0000000

# L:Solid:10.5%-L:Liquid:10.5% 0.32680117 -0.233017663 0.886619997 0.7004443

# M:Solid:10.5%-L:Liquid:10.5% 0.24793400 -0.311884830 0.807752830 0.9327857

# L:Liquid:5.25%-L:Liquid:10.5% 0.05254917 -0.507269663 0.612367997 1.0000000

# M:Liquid:5.25%-L:Liquid:10.5% -0.06156667 -0.621385497 0.498252163 0.9999998

# L:Solid:5.25%-L:Liquid:10.5% 0.37959550 -0.180223330 0.939414330 0.4833640

# M:Solid:5.25%-L:Liquid:10.5% 0.11164200 -0.448176830 0.671460830 0.9999245

# L:Solid:10.5%-M:Liquid:10.5% 0.35903083 -0.200787997 0.918849663 0.5685366

# M:Solid:10.5%-M:Liquid:10.5% 0.28016367 -0.279655163 0.839982497 0.8597436

# L:Liquid:5.25%-M:Liquid:10.5% 0.08477883 -0.475039997 0.644597663 0.9999954

# M:Liquid:5.25%-M:Liquid:10.5% -0.02933700 -0.589155830 0.530481830 1.0000000

# L:Solid:5.25%-M:Liquid:10.5% 0.41182517 -0.147993663 0.971643997 0.3585233

# M:Solid:5.25%-M:Liquid:10.5% 0.14387167 -0.415947163 0.703690497 0.9991430

# M:Solid:10.5%-L:Solid:10.5% -0.07886717 -0.638685997 0.480951663 0.9999978

# L:Liquid:5.25%-L:Solid:10.5% -0.27425200 -0.834070830 0.285566830 0.8756333

# M:Liquid:5.25%-L:Solid:10.5% -0.38836783 -0.948186663 0.171450997 0.4479760

# L:Solid:5.25%-L:Solid:10.5% 0.05279433 -0.507024497 0.612613163 1.0000000

# M:Solid:5.25%-L:Solid:10.5% -0.21515917 -0.774977997 0.344659663 0.9748748

# L:Liquid:5.25%-M:Solid:10.5% -0.19538483 -0.755203663 0.364433997 0.9879995

# M:Liquid:5.25%-M:Solid:10.5% -0.30950067 -0.869319497 0.250318163 0.7656724

# L:Solid:5.25%-M:Solid:10.5% 0.13166150 -0.428157330 0.691480330 0.9996262

# M:Solid:5.25%-M:Solid:10.5% -0.13629200 -0.696110830 0.423526830 0.9994820

# M:Liquid:5.25%-L:Liquid:5.25% -0.11411583 -0.673934663 0.445702997 0.9999063

# L:Solid:5.25%-L:Liquid:5.25% 0.32704633 -0.232772497 0.886865163 0.6994812

# M:Solid:5.25%-L:Liquid:5.25% 0.05909283 -0.500725997 0.618911663 0.9999999

# L:Solid:5.25%-M:Liquid:5.25% 0.44116217 -0.118656663 1.000980997 0.2611858

# M:Solid:5.25%-M:Liquid:5.25% 0.17320867 -0.386610163 0.733027497 0.9955463

# M:Solid:5.25%-L:Solid:5.25% -0.26795350 -0.827772330 0.291865330 0.8913379

summary(aov.shannon.all)

# Df Sum Sq Mean Sq F value Pr(>F)

# RDP 1 0.505 0.5051 6.219 0.0155 *

# sample.type 1 2.273 2.2730 27.987 1.88e-06 ***

# molas 2 0.069 0.0343 0.422 0.6578

# RDP:sample.type 1 0.007 0.0066 0.081 0.7768

# RDP:molas 2 0.112 0.0562 0.693 0.5043

# sample.type:molas 2 0.252 0.1258 1.548 0.2211

# RDP:sample.type:molas 2 0.047 0.0236 0.291 0.7487

# Residuals 59 4.792 0.0812

# ---

# Signif. codes: 0 ‘***’ 0.001 ‘**’ 0.01 ‘*’ 0.05 ‘.’ 0.1 ‘ ’ 1

aov.shannon.all2 = aov(shannon ~ RDP + sample.type + molas + RDP:molas + sample.type:molas + RDP:sample.type:molas , data=meta)

summary(aov.shannon.all2)

# Df Sum Sq Mean Sq F value Pr(>F)

# RDP 1 0.505 0.5051 6.219 0.0155 *

# sample.type 1 2.273 2.2730 27.987 1.88e-06 ***

# molas 2 0.069 0.0343 0.422 0.6578

# RDP:molas 2 0.112 0.0558 0.687 0.5070

# sample.type:molas 2 0.253 0.1266 1.559 0.2188

# RDP:sample.type:molas 3 0.053 0.0176 0.217 0.8841

# Residuals 59 4.792 0.0812

# ---

# Signif. codes: 0 ‘***’ 0.001 ‘**’ 0.01 ‘*’ 0.05 ‘.’ 0.1 ‘ ’ 1

aov.shannon.all3 = aov(shannon ~ RDP + sample.type + molas + RDP:molas + sample.type:molas , data=meta)

summary(aov.shannon.all3)

# Df Sum Sq Mean Sq F value Pr(>F)

# RDP 1 0.505 0.5051 6.464 0.0135 *

# sample.type 1 2.273 2.2730 29.089 1.15e-06 ***

# molas 2 0.069 0.0343 0.438 0.6470

# RDP:molas 2 0.112 0.0558 0.714 0.4935

# sample.type:molas 2 0.253 0.1266 1.621 0.2060

# Residuals 62 4.845 0.0781

# ---

# Signif. codes: 0 ‘***’ 0.001 ‘**’ 0.01 ‘*’ 0.05 ‘.’ 0.1 ‘ ’ 1

aov.shannon.all4 = aov(shannon ~ RDP + sample.type + RDP:molas + sample.type:molas , data=meta)

summary(aov.shannon.all4)

# Df Sum Sq Mean Sq F value Pr(>F)

# RDP 1 0.505 0.5051 6.464 0.0135 *

# sample.type 1 2.273 2.2730 29.089 1.15e-06 ***

# RDP:molas 4 0.180 0.0450 0.576 0.6808

# sample.type:molas 2 0.253 0.1266 1.621 0.2060

# Residuals 62 4.845 0.0781

# ---

# Signif. codes: 0 ‘***’ 0.001 ‘**’ 0.01 ‘*’ 0.05 ‘.’ 0.1 ‘ ’ 1

aov.shannon.all5 = aov(shannon ~ RDP + sample.type + sample.type:molas , data=meta)

summary(aov.shannon.all5)

# Df Sum Sq Mean Sq F value Pr(>F)

# RDP 1 0.505 0.5051 6.531 0.013 *

# sample.type 1 2.273 2.2730 29.393 9.61e-07 ***

# sample.type:molas 4 0.329 0.0822 1.063 0.382

# Residuals 64 4.949 0.0773

# ---

# Signif. codes: 0 ‘***’ 0.001 ‘**’ 0.01 ‘*’ 0.05 ‘.’ 0.1 ‘ ’ 1

aov.shannon.all6 = aov(shannon ~ RDP + sample.type, data=meta)

summary(aov.shannon.all6)

# Df Sum Sq Mean Sq F value Pr(>F)

# RDP 1 0.505 0.5051 6.507 0.013 *

# sample.type 1 2.273 2.2730 29.284 8.78e-07 ***

# Residuals 68 5.278 0.0776

# ---

# Signif. codes: 0 ‘***’ 0.001 ‘**’ 0.01 ‘*’ 0.05 ‘.’ 0.1 ‘ ’ 1

rs1 <-regsubsets( shannon~ as.factor(RDP)*as.factor(sample.type)*as.factor(molas), data=meta, nbest = 3, nvmax = 3)

plot(rs1, scale="bic")

#CHAO MODEL SELECTION

rs2 <-regsubsets( chao~ RDP*sample.type*molas, data=meta, nbest = 3, nvmax = 3)

plot(rs2, scale="bic")

> aov.chao.all = aov(chao ~ RDP*sample.type*molas, data=meta)

> summary(aov.chao.all)

# Df Sum Sq Mean Sq F value Pr(>F)

# RDP 1 457291 457291 5.802 0.0191 *

# sample.type 1 31465 31465 0.399 0.5299

# molas 2 49461 24730 0.314 0.7319

# RDP:sample.type 1 0 0 0.000 0.9993

# RDP:molas 2 33428 16714 0.212 0.8095

# sample.type:molas 2 7288 3644 0.046 0.9549

# RDP:sample.type:molas 2 324797 162398 2.060 0.1365

# Residuals 59 4650150 78816

# ---

# Signif. codes: 0 ‘***’ 0.001 ‘**’ 0.01 ‘*’ 0.05 ‘.’ 0.1 ‘ ’ 1

> aov.shannon.all2 = aov(shannon ~ RDP + sample.type + molas + RDP:molas + sample.type:molas + RDP:sample.type:molas , data=meta)

> summary(aov.shannon.all2)

# Df Sum Sq

kruskal.pretty(OTU.clean.S, meta.S, simper.results.S, c('RDP','molas','diets'), 'Simper.S', tax)

If we import the Kruskal-Wallis back into R and select only OTUs there were significantly different after fdr correction (fdr_krusk_p.val)…

#Import

KW.results.L = data.frame(read.csv("Simper.L_krusk_simper.csv"))

#Remove non-significant

KW.results.signif.L = KW.results.L[KW.results.L$fdr_krusk_p.val < 0.05,]

#Order by OTU#

KW.results.signif.L = KW.results.signif.L[with(KW.results.signif.L, order(OTU)),]

head(KW.results.signif.L)

#Import

KW.results.S = data.frame(read.csv("Simper.S_krusk_simper.csv"))

#Remove non-significant

KW.results.signif.S = KW.results.S[KW.results.S$fdr_krusk_p.val < 0.05,]

#Order by OTU#

KW.results.signif.S = KW.results.signif.S[with(KW.results.signif.S, order(OTU)),]

head(KW.results.signif.S)

ALL SAMPLE

simper.pretty(OTU.clean, meta, c('RDP','molas','diets'), perc_cutoff=1, low_cutoff = 'y', low_val=0.01, '2.Simper')

simper.results = data.frame(read.csv("2.Simper_clean_simper.csv"))

kruskal.pretty(OTU.clean, meta, simper.results, c('RDP','molas','diets'), 'Simper', tax)

#Import

KW.results = data.frame(read.csv("2.Simper_krusk_simper.csv"))

#Remove non-significant

KW.results.signif = KW.results[KW.results$fdr_krusk_p.val < 0.05,]

#Order by OTU#

KW.results.signif = KW.results.signif[with(KW.results.signif, order(OTU)),]

head(KW.results.signif)

PERIOD

simper.pretty(OTU.clean, meta, c('Period'), perc_cutoff=1, low_cutoff = 'y', low_val=0.01, 'Period')

Period.results = data.frame(read.csv("Period_clean_simper.csv"))

kruskal.pretty(OTU.clean, meta, Period.results, c('Period'), 'Period', tax)

#Import

KW.results2 = data.frame(read.csv("Period_krusk_simper.csv"))

#Remove non-significant

KW.results.signif2 = KW.results2[KW.results2$fdr_krusk_p.val < 0.05,]

#Order by OTU#

KW.results.signif2 = KW.results.signif2[with(KW.results.signif2, order(OTU)),]

head(KW.results.signif2)

SAMPLE.TYPE (THERE ARE SIGNIFICANT OTUs)

simper.pretty(OTU.clean, meta, c('sample.type'), perc_cutoff=1, low_cutoff = 'y', low_val=0.01, 'S.type')

S.type.results = data.frame(read.csv("S.type_clean_simper.csv"))

kruskal.pretty(OTU.clean, meta, S.type.results, c('sample.type'), 'S.type', tax)

#Import

KW.results1 = data.frame(read.csv("S.type_krusk_simper.csv"))

#Remove non-significant

KW.results.signif1 = KW.results1[KW.results1$fdr_krusk_p.val < 0.05,]

#Order by OTU#

KW.results.signif1 = KW.results.signif1[with(KW.results.signif1, order(OTU)),]

head(KW.results.signif1)

#Second version

simper.pretty(OTU.clean, meta, c('sample.type'), perc_cutoff=1, low_cutoff = 'y', low_val=0.001, 'S.type2')

S.type.results = data.frame(read.csv("S.type2_clean_simper.csv"))

kruskal.pretty(OTU.clean, meta, S.type.results, c('sample.type'), 'S.type2', tax)

#Import

KW.results1 = data.frame(read.csv("S.type2_krusk_simper.csv"))

#Remove non-significant

KW.results.signif1 = KW.results1[KW.results1$fdr_krusk_p.val < 0.05,]

#Order by OTU#

KW.results.signif1 = KW.results.signif1[with(KW.results.signif1, order(OTU)),]

head(KW.results.signif1)

abund = OTU.clean/rowSums(OTU.clean)*100

#plot

boxplot(abund$Otu00001 ~ meta$sample.type, ylab="% Relative abundance", main="OTU1")

boxplot(abund$Otu00007 ~ meta$sample.type, ylab="% Relative abundance", main="OTU1")

boxplot(abund$Otu00007 ~ meta$sample.type, ylab="% Relative abundance", main="OTU1")

boxplot(abund$Otu00007 ~ meta$sample.type, ylab="% Relative abundance", main="OTU1")

boxplot(abund$Otu00007 ~ meta$sample.type, ylab="% Relative abundance", main="OTU1")

boxplot(abund$Otu00007 ~ meta$sample.type, ylab="% Relative abundance", main="OTU1")

boxplot(abund$Otu00007 ~ meta$sample.type, ylab="% Relative abundance", main="OTU1")

boxplot(abund$Otu00007 ~ meta$sample.type, ylab="% Relative abundance", main="OTU1")

DEseq2

pds <- phyloseq_to_deseq2(physeq, ~RDP) #REQUIRES PHSEQ.TREE

Correlations

So, you can also approach continuous variables as correlations. Generally, only strong correlations (r > 0.5 or r < -0.5) should be reported and if you have a lot that fall into the “strong” category, you can up the cut off, say, to r > 0.75 or r < -0.75. There are many correlation options. I like Kendall-Tau because it does not assume linearity or normality. Type ??cor in the R console to learn others that are available.

Also, consider options to decrease the number of OTUs tested or you will be dealing with a huge table. Like only ones at >X% abundance? Only ones found in SIMPER and/or KW analyses of other important variables?

Here, we will correlate RDP to OTUs with at least 5% relative abundance in at least one sample in our data set.

#Remember we calculated abundance before with

#abund = OTU.clean/rowSums(OTU.clean)*100

#Subset OTUs to abundance cutoff

OTU.abund = OTU.clean[, apply(abund, MARGIN=2, function(x) any(x > 5))]

cor.kendall = cor(OTU.abund, meta$RDP, method = "kendall")

cor.kendall

Working in phyloseq

phyloseq is an incredibly powerful package, and we will not have time to go over everything that it is able to do. There is abundant documentation and tutorials online. Here is a link to the phyloseq [bible.](http://joey711.github.io/phyloseq-demo/) But I would like to go over some of the ways you can manipulate data in phyloseq which might come in handy. We will make a new phyloseq object first, using our previously generated phyloseq-formatted input files. The object is much smaller without the tree, so I am not going to include it.

physeq.demo <- phyloseq(OTU.UF, meta.UF, tax.UF)

Here are some accessors to take a look at your phyloseq object.

#View one of the data frames within the object

View(sample_data(physeq.demo))

#number of taxa

ntaxa(physeq.demo)

nsamples(physeq.demo)

sample_names(physeq.demo)

rank_names(physeq.demo)

sample_variables(physeq.demo)

Beta-diversity HEATMAP

The other common use for heatmaps is to show distances between samples (i.e. beta-diversity) similar to what is shown in nMDS. We have all of the same metric options as we did for nMDS.

We do not want to use the plot_heatmap function from phyloseq because it requires the input of a physeq object. Instead, we can use our distance matrices as inputs for a gplots command. This command will automatically group samples by similarity (trees)

#Bray-Curtis

pdf("heatmap.RDP.L.pdf", width = 7, height = 3, colormodel = "rgb", family = "ArialMT")

heatmap.2(as.matrix(BC.dist.L))

**Supplementary Text 5.** Code-4 for Microbiota Analysis in R.

---

title: "Supplementary Text 5"

output: html_document

---

#2D variables

These analyses are for comparing the microbiota to metadata that cannot fit in a single column and therefore, must be represented as a matrix of its own. For example, PERMANOVA can only tell you that the microbiota differs according to a single short chain fatty acid (SCFA), but other tests can tell you that the microbiota differs according to the overall SCFA profile. This section is also useful for comparing data if you have multiple OTU tables, like for bacteria, archaea, and fungi.

Mantel from vegan tests if two distance matrices co-vary e.g. does the data in matrix 1 change in the same way as the data in matrix 2. Like PERMANOVA, this test only tells you that the overall data co-vary, not which specific OTUs or SCFAs matter. You can only compare samples were you have both types of data so we must use the subsetted SCFA OTU table we made earlier.

We then calculate distance matrices separately for each matrix. It is not necessary to do Bray-Curtis, Jaccard and UniFrac here since our SCFA data does not have any taxonomy to it.

```{r}

write.table(OTU.clean.relabund, file = "OTU.clean.relabund.csv", sep = ",", col.names = NA,

qmethod = "double")

dist1 = vegdist(OTU.clean.L)

dist2 = vegdist(SCFA.L)

mantel(dist1, dist2, permutations=100)

Mantel statistic based on Pearson's product-moment correlation

Call:

mantel(xdis = dist1, ydis = dist2, permutations = 100)

Mantel statistic r: 0.1863

Significance: 0.019802

Upper quantiles of permutations (null model):

90% 95% 97.5% 99%

0.104 0.142 0.167 0.180

Permutation: free

Number of permutations: 100

```{r, echo=FALSE}

dist3 = vegdist(OTU.clean.S)

dist4 = vegdist(SCFA.S)

mantel(dist3, dist4, permutations=100)

```

Mantel statistic based on Pearson's product-moment correlation

Call:

mantel(xdis = dist3, ydis = dist4, permutations = 100)

Mantel statistic r: 0.159

Significance: 0.049505

Upper quantiles of permutations (null model):

90% 95% 97.5% 99%

0.0892 0.1458 0.1749 0.2166

Permutation: free

Number of permutations: 100

```{r}

library(car)

leveneTest(OTU.clean.relabund$Otu00001 ~ Period*RDP*molas, data=meta)

```

Levene's Test for Homogeneity of Variance (center = median)

Df F value Pr(>F)

group 17 1.5784 0.1041

53

```{r}

leveneTest(OTU.clean.relabund$Otu00001 ~ RDP*molas, data=meta)

```

So Variances are homogenious: we can use the PERMANOVA FOR

Levene's Test for Homogeneity of Variance (center = median)

Df F value Pr(>F)

group 5 1.2253 0.3074

65

```{r}

library("lme4")

library("lsmeans")

OTU.lm<-lmer(OTU.clean.relabund$Otu00001~sample.type+Period+RDP+molas+RDP:molas + (1|Animal), data =meta)

(OTU.rg1 <- ref.grid(OTU.lm) )

# 'ref.grid' object with variables:

# sample.type = Liquid, Solid

# Period = P1, P2, P3

# RDP = L, M

# molas = 0%, 10.5%, 5.25%

str(lsmeans(OTU.rg1,~RDP*molas*Period*sample.type))

# 'lsmobj' object with variables:

# RDP = L, M

# molas = 0%, 10.5%, 5.25%

# Period = P1, P2, P3

# sample.type = Liquid, Solid

OTU1.lsm<- lsmeans(OTU.rg1,~RDP*molas*Period*sample.type)

(OTU1.sum <- summary(OTU1.lsm, infer = c(TRUE,TRUE),

level = .95, adjust = "bon", by = " sample.type" ))

class(OTU1.sum)

```

```{r}

OTU.clean2.relabund = OTU.clean.relabund[,which(names(OTU.clean.relabund) %in% c("Otu00840", "Otu00314","Otu00676","Otu00114", "Otu00865", "Otu00087","Otu00667","Otu00899","Otu00678","Otu00936","Otu00370","Otu00282","Otu00652","Otu00716","Otu00946","Otu00412","Otu00001","Otu00005","Otu00014","Otu0002","Otu00003","Otu00006","Otu00015","Otu00013","Otu00002","Otu00019"))]

tax.clean.ST.relabund = tax.clean[which(row.names(tax.clean)%in%c("Otu00001","Otu00005","Otu00014","Otu00022","Otu00003","Otu00006","Otu00015","Otu00013","Otu00019","Otu00002")),]

write.table(tax.clean.ST.relabund, file = "tax.ST.csv", sep = ",", col.names = NA,

qmethod = "double")

tax.clean.RDP.relabund = tax.clean[which(row.names(tax.clean)%in% row.names(KW.results.signif)),]

write.table(tax.clean.RDP.relabund, file = "tax.RDP.csv", sep = ",", col.names = NA,

qmethod = "double")

row.names(KW.results.signif) <- KW.results.signif$OTU # MAKE OTU NAMES ROW NAMES FOR NEXT %in% STEP

tax.clean.MOL.relabund = tax.clean[which(row.names(tax.clean)%in% row.names(KW.results.signif)),]

write.table(tax.clean.MOL.relabund, file = "tax.MOL.csv", sep = ",", col.names = NA,

qmethod = "double")

```

SIMPER FOR ALL SAMPLE

```{r}

simper.pretty(OTU.clean, meta, c('RDP','molas','diets'), perc_cutoff=1, low_cutoff = 'y', low_val=0.01, 'Simper2')

simper.results = data.frame(read.csv("Simper2_clean_simper.csv"))

kruskal.pretty(OTU.clean, meta, simper.results, c('RDP','molas','diets'), 'Simper2', tax)

#Import

KW.results = data.frame(read.csv("Simper2_krusk_simper.csv"))

#Remove non-significant

KW.results.signif = KW.results[KW.results$fdr_krusk_p.val < 0.05,]

#Order by OTU#

KW.results.signif = KW.results.signif[with(KW.results.signif, order(OTU)),]

head(KW.results.signif)

```

PERIOD

```{r}

simper.pretty(OTU.clean, meta, c('Period'), perc_cutoff=1, low_cutoff = 'y', low_val=0.01, 'SimperPERIOD')

simper.results = data.frame(read.csv("SimperPERIOD_clean_simper.csv"))

kruskal.pretty(OTU.clean, meta, simper.results, c('Period'), 'SimperPERIOD', tax.clean)

#Import

KW.results = data.frame(read.csv("SimperPERIOD_krusk_simper.csv"))

#Remove non-significant

KW.results.signif = KW.results[KW.results$fdr_krusk_p.val < 0.1,]

#Order by OTU#

KW.results.signif = KW.results.signif[with(KW.results.signif, order(OTU)),]

head(KW.results.signif)

```

MOLASSES

```{r}

simper.pretty(OTU.clean, meta, c('molas'), perc_cutoff=1, low_cutoff = 'y', low_val=0.01, 'SimperMOLAS')

simper.results = data.frame(read.csv("SimperMOLAS_clean_simper.csv"))

kruskal.pretty(OTU.clean, meta, simper.results, c('molas'), 'SimperMOLAS', tax.clean)

#Import

KW.results = data.frame(read.csv("SimperMOLAS_krusk_simper.csv"))

#Remove non-significant

KW.results.signif = KW.results[KW.results$fdr_krusk_p.val < 0.1,]

#Order by OTU#

KW.results.signif = KW.results.signif[with(KW.results.signif, order(OTU)),]

head(KW.results.signif)

```

DIETS

```{r}

simper.pretty(OTU.clean, meta, c('diets'), perc_cutoff=1, low_cutoff = 'y', low_val=0.01, 'SimperDIETS')

simper.results = data.frame(read.csv("SimperDIETS_clean_simper.csv"))

kruskal.pretty(OTU.clean, meta, simper.results, c('diets'), 'SimperDIETS', tax.clean)

#Import

KW.results = data.frame(read.csv("SimperDIETS_krusk_simper.csv"))

#Remove non-significant

KW.results.signif = KW.results[KW.results$fdr_krusk_p.val < 0.1,]

#Order by OTU#

KW.results.signif = KW.results.signif[with(KW.results.signif, order(OTU)),]

head(KW.results.signif)

```

RDP

```{r}

simper.pretty(OTU.clean, meta, c('RDP'), perc_cutoff=1, low_cutoff = 'y', low_val=0.01, 'SimperRDP')

simper.results = data.frame(read.csv("SimperRDP_clean_simper.csv"))

kruskal.pretty(OTU.clean, meta, simper.results, c('RDP'), 'SimperRDP', tax.clean)

#Import

KW.results = data.frame(read.csv("SimperRDP_krusk_simper.csv"))

#Remove non-significant

KW.results.signif = KW.results[KW.results$fdr_krusk_p.val < 0.1,]

#Order by OTU#

KW.results.signif = KW.results.signif[with(KW.results.signif, order(OTU)),]

head(KW.results.signif)

```

SAMPLE TYPE

```{r}

simper.pretty(OTU.clean, meta, c('sample.type'), perc_cutoff=1, low_cutoff = 'y', low_val=0.01, 'SimperST')

simper.results = data.frame(read.csv("SimperST_clean_simper.csv"))

kruskal.pretty(OTU.clean, meta, simper.results, c('sample.type'), 'SimperST', tax.clean)

#Import

KW.results = data.frame(read.csv("SimperST_krusk_simper.csv"))

#Remove non-significant

KW.results.signif = KW.results[KW.results$fdr_krusk_p.val < 0.1,]

#Order by OTU#

KW.results.signif = KW.results.signif[with(KW.results.signif, order(OTU)),]

head(KW.results.signif)

```

LOW_VAL=0.005

PERIOD

```{r}

simper.pretty(OTU.clean, meta, c('Period'), perc_cutoff=1, low_cutoff = 'y', low_val=0.005, 'SimperPERIOD05')

simper.results = data.frame(read.csv("SimperPERIOD05_clean_simper.csv"))

kruskal.pretty(OTU.clean, meta, simper.results, c('Period'), 'SimperPERIOD05', tax.clean)

#Import

KW.results = data.frame(read.csv("SimperPERIOD05_krusk_simper.csv"))

#Remove non-significant

KW.results.signif = KW.results[KW.results$fdr_krusk_p.val < 0.1,]

#Order by OTU#

KW.results.signif = KW.results.signif[with(KW.results.signif, order(OTU)),]

head(KW.results.signif)

```

MOLASSES

```{r}

simper.pretty(OTU.clean, meta, c('molas'), perc_cutoff=1, low_cutoff = 'y', low_val=0.001, 'SimperMOLAS01')

simper.results = data.frame(read.csv("SimperMOLAS01_clean_simper.csv"))

kruskal.pretty(OTU.clean, meta, simper.results, c('molas'), 'SimperMOLAS01', tax.clean)

#Import

KW.results = data.frame(read.csv("SimperMOLAS01_krusk_simper.csv"))

#Remove non-significant

KW.results.signif = KW.results[KW.results$fdr_krusk_p.val < 0.1,]

#Order by OTU#

KW.results.signif = KW.results.signif[with(KW.results.signif, order(OTU)),]

head(KW.results.signif)

```

DIETS

```{r}

simper.pretty(OTU.clean, meta, c('diets'), perc_cutoff=1, low_cutoff = 'y', low_val=0.001, 'SimperDIETS01')

simper.results = data.frame(read.csv("SimperDIETS01_clean_simper.csv"))

kruskal.pretty(OTU.clean, meta, simper.results, c('diets'), 'SimperDIETS01', tax.clean)

#Import

KW.results = data.frame(read.csv("SimperDIETS01_krusk_simper.csv"))

#Remove non-significant

KW.results.signif = KW.results[KW.results$fdr_krusk_p.val < 0.1,]

#Order by OTU#

KW.results.signif = KW.results.signif[with(KW.results.signif, order(OTU)),]

head(KW.results.signif)

```

RDP

```{r}

simper.pretty(OTU.clean, meta, c('RDP'), perc_cutoff=1, low_cutoff = 'y', low_val=0.001, 'SimperRDP01')

simper.results = data.frame(read.csv("SimperRDP01_clean_simper.csv"))

kruskal.pretty(OTU.clean, meta, simper.results, c('RDP'), 'SimperRDP01', tax.clean)

#Import

KW.results = data.frame(read.csv("SimperRDP01_krusk_simper.csv"))

#Remove non-significant

KW.results.signif = KW.results[KW.results$fdr_krusk_p.val < 0.1,]

#Order by OTU#

KW.results.signif = KW.results.signif[with(KW.results.signif, order(OTU)),]

head(KW.results.signif)

```

SAMPLE TYPE

```{r}

simper.pretty(OTU.clean, meta, c('sample.type'), perc_cutoff=1, low_cutoff = 'y', low_val=0.001, 'SimperST01')

simper.results = data.frame(read.csv("SimperST01_clean_simper.csv"))

kruskal.pretty(OTU.clean, meta, simper.results, c('sample.type'), 'SimperST01', tax.clean)

#Import

KW.results = data.frame(read.csv("SimperST01_krusk_simper.csv"))

#Remove non-significant

KW.results.signif = KW.results[KW.results$fdr_krusk_p.val < 0.1,]

#Order by OTU#

KW.results.signif = KW.results.signif[with(KW.results.signif, order(OTU)),]

head(KW.results.signif)

```

CORRELATION STUDY

```{r}

tax.genus.count<-data.frame(read.table("tax_summed_genus.txt"))#READ .TXT FILE

tax.genus.count.S<-tax.genus.count[,-(1:36)]

tax.genus.count.L<-tax.genus.count[, 1:36]

genus.relabund.L<-t(tax.genus.count.L)/rowSums(t(tax.genus.count.L))*100

genus.relabund.S<-t(tax.genus.count.S)/rowSums(t(tax.genus.count.S))*100

genus.relabund.L <- data.frame(genus.relabund.L)

genus.relabund.S <- data.frame(genus.relabund.S)

genus.abund.L01 <- t(tax.genus.count.L)[, apply(genus.relabund.L, MARGIN=2, function(x) any(x > 0.1))]

genus.abund.S01 <- t(tax.genus.count.S)[, apply(genus.relabund.S, MARGIN=2, function(x) any(x > 0.1))]

genus.abund.L01<-data.frame(genus.abund.L01)

genus.abund.S01<-data.frame(genus.abund.S01)

genus.abund1 = genus.relabund[, apply(genus.relabund, MARGIN=2, function(x) any(x > 1))]

> SCFA.2<-data.frame(read.csv("RumenData.csv"))

> RCORR.L<-rcorr(as.matrix(cbind(genus.abund.L01,SCFA.2.COR)))

> RCORR.S<-rcorr(as.matrix(cbind(genus.abund.S01,SCFA.2.COR[-26,])))

>table.L<-flattenCorrMatrix*(RCORR.L$r, RCORR.L$P)

> table.S<-flattenCorrMatrix(RCORR.S$r, RCORR.S$P)

> write.table(RCORR.S$r, file = "RCORR.S.csv", sep = ",")

> write.table(RCORR.L$P, file = "COR.TABLE.L.pval.csv", sep = ",")

> write.table(RCORR.S$P, file = "COR.TABLE.S.pval.csv", sep = ",")

*SOURCE CODE IN microbiome_corrAn.R

Prevotella Succiniclasticum unclassified Coprococcus Butyrivibrio Treponema CF231 YRC22 Pseudobutyrivibrio Ruminococcus Lachnospira Anaerovibrio Clostridium Selenomonas Fibrobacter Schwartzia Desulfovibrio BF311 Shuttleworthia Ruminobacter Moryella Succinivibrio Mogibacterium Anaeroplasma Prevotella. Oscillospira RFN20 Pyramidobacter SHD.231 Asteroleplasma DMI DIM OrganicAcids pH Isobutyrate Isoval Ammonia TAA Lactate Acetate Propionate Butyrate Valerate

##############################################################################################

# 2.A. CORRELATION PLOT USING CORRPLOT

###############################################################################################

install.packages("corrplot")

library(corrplot)

corrplot(RCORR.L$r, p.mat= RCORR.L$P, sign.level=0.05, insig ="blank",type = "lower", order = "FPC", tl.srt = 0.5)

corrplot(RCORR.L$r, type = "upper", order = "original",

p.mat = RCORR.L$P,, sig.level = 0.05, insig = "blank", tl.col = "black", tl.srt = 90)

png("c4_solid.png",

width = 5*300, # 5 x 300 pixels

height = 5*300,

res = 300, # 300 pixels per inch

pointsize = 8.75)

pmatt_s<-read.csv("pvalue_s.csv", sep=",", row.names=1)

pmat11_s <- as.matrix(pmatt_s)

corrplot(RCORR_2$r, type = "upper", order = "original",

p.mat = pmat11_s, sig.level = 0.01, insig = "blank", tl.col = "black", tl.srt = 90)

#KENDALL CORRELATION

cor.kendall29.S = cor(genus.abund02[-(1:36),], SCFA.S, method = "kendall")

cor.kendall29.L = cor(genus.abund02[1:36,], SCFA.L, method = "kendall")

#PEARSON CORR

cor.pearson29.S = cor(genus.abund02[-(1:36),], SCFA.S, method = "pearson")

cor.pearson29.L = cor(genus.abund02[1:36,], SCFA.L, method = "pearson")

#WRITE THEM INTO .CSV

write.table(cor.kendall29.S, file = "cor_kendall29.S.csv", sep = ",")

write.table(cor.kendall29.L, file = "cor_kendall29.L.csv", sep = ",")

write.table(cor.pearson29.S, file = "cor_pearson29.S.csv", sep = ",")

write.table(cor.pearson29.L, file = "cor_pearson29.L.csv", sep = ",")

#################################################################################

#################################################################################

#g__Dialister boxplot

boxplot(genus.relabund$g__Dialister ~ meta$RDP, ylab="% Relative abundance", main="Dialister")#NOT SIGNIFICANT BY RDP

tax.genus.count.S<-tax.genus.count[,-(1:36)]

tax.genus.count.L<-tax.genus.count[,(1:36)]

cor.L<-bioenv(comm=t(tax.genus.count.L), env=SCFA.L, method="kendall", index="bray")

cor.S<-bioenv(comm=t(tax.genus.count.S), env=SCFA.S, method="kendall", index="bray")

```

OTHER VISUALISATIONS

```{r}

#LIQUIDS

OTU.UF.L = otu_table(as.matrix(OTU.clean.L), taxa_are_rows=FALSE)

tax.UF.L = tax_table(as.matrix(tax.clean.L))

meta.UF.L = sample_data(meta.L)

physeq.L = phyloseq(OTU.UF.L, tax.UF.L, meta.UF.L)

plot_bar(physeq.L, x="RDP", fill="Phylum") + geom_bar(aes(color=Phylum, fill=Phylum), stat="identity", position="stack")

#SOLIDS

OTU.UF.S = otu_table(as.matrix(OTU.clean.S), taxa_are_rows=FALSE)

tax.UF.S = tax_table(as.matrix(tax.clean.S))

meta.UF.S = sample_data(meta.S)

physeq.S = phyloseq(OTU.UF.S, tax.UF.S, meta.UF.S)

#PLOTS

#ALLSAMPLE LIQ VS SOLID

#par(mfrow(3,2))DOES NOT WORK SO THEN,

###########################################

# Multiple plot function

###########################################

# ggplot objects can be passed in ..., or to plotlist (as a list of ggplot objects)

# - cols: Number of columns in layout

# - layout: A matrix specifying the layout. If present, 'cols' is ignored.

#

# If the layout is something like matrix(c(1,2,3,3), nrow=2, byrow=TRUE),

# then plot 1 will go in the upper left, 2 will go in the upper right, and

# 3 will go all the way across the bottom.

#

multiplot <- function(..., plotlist=NULL, file, cols=1, layout=NULL) {

library(grid)

# Make a list from the ... arguments and plotlist

plots <- c(list(...), plotlist)

numPlots = length(plots)

# If layout is NULL, then use 'cols' to determine layout

if (is.null(layout)) {

# Make the panel

# ncol: Number of columns of plots

# nrow: Number of rows needed, calculated from # of cols

layout <- matrix(seq(1, cols * ceiling(numPlots/cols)),

ncol = cols, nrow = ceiling(numPlots/cols))

}

if (numPlots==1) {

print(plots[[1]])

} else {

# Set up the page

grid.newpage()

pushViewport(viewport(layout = grid.layout(nrow(layout), ncol(layout))))

# Make each plot, in the correct location

for (i in 1:numPlots) {

# Get the i,j matrix positions of the regions that contain this subplot

matchidx <- as.data.frame(which(layout == i, arr.ind = TRUE))

print(plots[[i]], vp = viewport(layout.pos.row = matchidx$row,

layout.pos.col = matchidx$col))

}

}

}

####################################################################################

multiplot(p1, p2, p3, p4, cols=2)

pdf("Fig9.pdf", width = 7, height = 8, colormodel = "rgb", family = "ArialMT")

plot_bar(physeq.L, fill="Phylum") + geom_bar(aes(color=Phylum, fill=Phylum), stat="identity", position="stack")

dev.off()

pdf("Fig10.pdf", width = 7, height = 8, colormodel = "rgb", family = "ArialMT")

plot_bar(physeq.S, fill="Phylum") + geom_bar(aes(color=Phylum, fill=Phylum), stat="identity", position="stack")

dev.off()

pdf("Fig11.pdf", width = 7, height = 8, colormodel = "rgb", family = "ArialMT")

plot_bar(physeq.L, x="RDP", fill="Phylum") + geom_bar(aes(color=Phylum, fill=Phylum), stat="identity", position="stack")

dev.off()

pdf("Fig12.pdf", width = 7, height = 8, colormodel = "rgb", family = "ArialMT")

plot_bar(physeq.S, x="RDP", fill="Phylum") + geom_bar(aes(color=Phylum, fill=Phylum), stat="identity", position="stack")

dev.off()

pdf("Fig13.pdf", width = 7, height = 8, colormodel = "rgb", family = "ArialMT")

plot_bar(physeq.L, x="molas", fill="Phylum") + geom_bar(aes(color=Phylum, fill=Phylum), stat="identity", position="stack")

dev.off()

pdf("Fig14.pdf", width = 7, height = 8, colormodel = "rgb", family = "ArialMT")

plot_bar(physeq.S, x="molas", fill="Phylum") + geom_bar(aes(color=Phylum, fill=Phylum), stat="identity", position="stack")

dev.off()

pdf("Fig15.pdf", width = 7, height = 8, colormodel = "rgb", family = "ArialMT")

plot_bar(physeq, fill="Phylum") + geom_bar(aes(color=Phylum, fill=Phylum), stat="identity", position="stack")

dev.off()

#Sort the GENUS by abundance and pick the top 13

top5P.names.L = sort(tapply(taxa_sums(physeq.L), tax_table(physeq.L)[, "Genus"], sum), TRUE)[1:13]

top5P.names.S = sort(tapply(taxa_sums(physeq.S), tax_table(physeq.S)[, "Genus"], sum), TRUE)[1:13]

#Cut down the physeq.tree data to only the top 13 GENUS

top5P.L = subset_taxa(physeq.L, Genus %in% names(top5P.names.L))

top5P.S = subset_taxa(physeq.S, Genus %in% names(top5P.names.S))

top5P.names.13 = sort(tapply(taxa_sums(physeq), tax_table(physeq)[, "Genus"], sum), TRUE)[1:13]

top5P.13 = subset_taxa(physeq, Genus %in% names(top5P.names.13))

#Plot

pdf("Fig16.pdf", width = 17, height = 10, colormodel = "rgb", family = "ArialMT")

plot_bar(top5P.L, x="RDP", fill="Genus") + geom_bar(aes(color=Genus, fill=Genus), stat="identity", position="stack")

dev.off()

#BY DIETS

pdf("Fig17.pdf", width = 17, height = 10, colormodel = "rgb", family = "ArialMT")

plot_bar(top5P.L, x="RDP", fill="Genus", facet_grid = ~Phylum) + geom_bar(aes(color=Genus, fill=Genus), stat="identity", position="stack")

dev.off()

pdf("Fig18.pdf", width = 17, height = 10, colormodel = "rgb", family = "ArialMT")

plot_bar(top5P.S, x="diets", fill="Genus", facet_grid = ~Phylum) + geom_bar(aes(color=Genus, fill=Genus), stat="identity", position="stack")

dev.off()

pdf("Fig17.pdf", width = 17, height = 10, colormodel = "rgb", family = "ArialMT")

plot_bar(top5P.L, x="diets", fill="Genus", facet_grid = ~Phylum) + geom_bar(aes(color=Genus, fill=Genus), stat="identity", position="stack")

dev.off()

#BY RDP

pdf("Fig19.pdf", width = 17, height = 10, colormodel = "rgb", family = "ArialMT")

plot_bar(top5P.L, x="RDP", fill="Genus", facet_grid = ~Phylum) + geom_bar(aes(color=Genus, fill=Genus), stat="identity", position="stack")

dev.off()

pdf("Fig20.pdf", width = 17, height = 10, colormodel = "rgb", family = "ArialMT")

plot_bar(top5P.S, x="RDP", fill="Genus", facet_grid = ~Phylum) + geom_bar(aes(color=Genus, fill=Genus), stat="identity", position="stack")

dev.off()

plot_bar(top5P.L, "diets", fill="Genus", facet_grid=~Family)

dev.off()

#BY MOLAS

pdf("Fig21.pdf", width = 17, height = 10, colormodel = "rgb", family = "ArialMT")

plot_bar(top5P.L, x="molas", fill="Genus", facet_grid = ~Phylum) + geom_bar(aes(color=Genus, fill=Genus), stat="identity", position="stack")

dev.off()

pdf("Fig22.pdf", width = 17, height = 10, colormodel = "rgb", family = "ArialMT")

plot_bar(top5P.S, x="molas", fill="Genus", facet_grid = ~Phylum) + geom_bar(aes(color=Genus, fill=Genus), stat="identity", position="stack")

dev.off()

#WITH MULTIPLE PLOT

pdf("Fig27.pdf", width = 24, height = 15, colormodel = "rgb", family = "ArialMT")

#p1<-plot_bar(top5P.13, x="sample.type", fill="Genus", facet_grid = ~Phylum) + geom_bar(aes(color=Genus, fill=Genus), stat="identity", position="stack")

p1<-plot_bar(top5P.L, x="RDP", fill="Genus", facet_grid = ~Phylum), stat="identity", position="stack")

p2<-plot_bar(top5P.S, x="RDP", fill="Genus", facet_grid = ~Phylum) + geom_bar(aes(color=Genus, fill=Genus), stat="identity", position="stack")

p3<-plot_bar(top5P.L, x="molas", fill="Genus", facet_grid = ~Phylum), stat="identity", position="stack")

p4<-plot_bar(top5P.S, x="molas", fill="Genus", facet_grid = ~Phylum) + geom_bar(aes(color=Genus, fill=Genus), stat="identity", position="stack")

multiplot(p1, p2, p3, p4, cols=2)

dev.off()

pdf("Fig29.pdf", width = 24, height = 15, colormodel = "rgb", family = "ArialMT")

plot_bar(top5P.13, x="sample.type", fill="Genus", facet_grid = ~Phylum) + geom_bar(aes(color=Genus, fill=Genus), stat="identity", position="stack")

dev.off()

pdf("Fig30.pdf", width = 24, height = 15, colormodel = "rgb", family = "ArialMT")

plot_bar(prevotella, x="RDP", fill="Genus", facet_grid = ~sample.type) + geom_bar(aes(color=Genus, fill=Genus), stat="identity", position="stack")

dev.off()

pdf("Fig31.pdf", width = 24, height = 15, colormodel = "rgb", family = "ArialMT")

c1<-plot_bar(top5P.L, x="RDP", fill="Genus", facet_grid = ~Genus) + geom_bar(aes(color=Genus, fill=Genus), stat="identity", position="stack")

c2<-plot_bar(top5P.S, x="RDP", fill="Genus", facet_grid = ~Genus) + geom_bar(aes(color=Genus, fill=Genus), stat="identity", position="stack")

multiplot(c1, c2, cols=2)

dev.off()

plot_bar(top5P.L, "diets", fill="Genus", facet_grid=~Family)

dev.off()

#Sort the FAMILY by abundance and pick the top 10

top5P.names.FL = sort(tapply(taxa_sums(physeq.L), tax_table(physeq.L)[, "Family"], sum), TRUE)[1:10]

top5P.names.FS = sort(tapply(taxa_sums(physeq.S), tax_table(physeq.S)[, "Family"], sum), TRUE)[1:10]

#Cut down the physeq.tree data to only the top 10 GENUS

top5P.FL = subset_taxa(physeq.L, Family %in% names(top5P.names.FL))

top5P.FS = subset_taxa(physeq.S, Family %in% names(top5P.names.FS))

#Plot

#BY RDP FAMILY FILL

pdf("Fig23.pdf", width = 17, height = 10, colormodel = "rgb", family = "ArialMT")

plot_bar(top5P.FL, x="RDP", fill="Family", facet_grid = ~Phylum) + geom_bar(aes(color=Family, fill=Family), stat="identity", position="stack")

dev.off()

pdf("Fig24.pdf", width = 17, height = 10, colormodel = "rgb", family = "ArialMT")

plot_bar(top5P.FS, x="RDP", fill="Family", facet_grid = ~Phylum) + geom_bar(aes(color=Family, fill=Family), stat="identity", position="stack")

dev.off()

#BY MOLAS

pdf("Fig25.pdf", width = 17, height = 10, colormodel = "rgb", family = "ArialMT")

plot_bar(top5P.FL, x="molas", fill="Family", facet_grid = ~Phylum) + geom_bar(aes(color=Family, fill=Family), stat="identity", position="stack")

dev.off()

pdf("Fig26.pdf", width = 17, height = 10, colormodel = "rgb", family = "ArialMT")

plot_bar(top5P.FS, x="molas", fill="Family", facet_grid = ~Phylum) + geom_bar(aes(color=Family, fill=Family), stat="identity", position="stack")

dev.off()

# plot_bar(top5P.FL, "diets", fill="Genus", facet_grid=~Family)

# dev.off()

# plot_bar(top5P, x="diets", fill="Genus")

```

#UNCLASSIFIED

```{r}

L1 = subset_taxa(physeq.L, Genus == "f__Succinivibrionaceae_unclassified")

L2 = subset_taxa(physeq.L, Genus == "c__Gammaproteobacteria_unclassified")#phylum Proteobacteria

L3 = subset_taxa(physeq.L, Genus == "f__BS11_unclassified") #phylum Bacteroidetes

L4 = subset_taxa(physeq.L, Genus == "o__Clostridiales_unclassified")

L5 = subset_taxa(physeq.L, Genus == "f__Lachnospiraceae_unclassified")

L6 = subset_taxa(physeq.L, Genus == "f__Lachnospiraceae_unclassified")

S1 = subset_taxa(physeq.S, Genus == "p__Firmicutes_unclassified") #ntaxa(S1) = 1

S2 = subset_taxa(physeq.S, Genus == "p__Firmicutes_unclassified")

S3 = subset_taxa(physeq.S, Genus == "p__Bacteroidetes_unclassified")

S4 = subset_taxa(physeq.S, Genus == "p__Bacteroidetes_unclassified")

S5 = subset_taxa(physeq.S, Genus == "f__Lachnospiraceae_unclassified")

S6 = subset_taxa(physeq.S, Genus == "p__Bacteroidetes_unclassified")

S7 = subset_taxa(physeq.S, Genus == "p__Bacteroidetes_unclassified")

S8 = subset_taxa(physeq.S, Genus == "o__Bacteroidales_unclassified")

nL = subset_taxa(physeq.L, Genus == "") #"o__Clostridiales_unclassified")

n = subset_taxa(physeq.S, Genus == "") #"o__Bacteroidales_unclassified"

```

sadece en az bir sample icin yuzde 1 uzerindeki contribution i saglayan OTUs ve analizi(50 ADET OTU)

```{r}

abund = OTU.clean/rowSums(OTU.clean)*100

OTU.abund1p = OTU.clean[, apply(abund, MARGIN=2, function(x) any(x > 1))]

tax.clean[row.names(tax.clean)%in%colnames(OTU.abund1p),]$Genus #en az bir sample icin yuzde 1 uzerindeki contribution i saglayan OTUs generasi

top50.relabund<-OTU.clean.relabund[,colnames(OTU.clean.relabund)%in%colnames(OTU.abund1p)]

OTU.abund02p = OTU.clean[, apply(abund, MARGIN=2, function(x) any(x > 0.2))]

OTU.clean.relabund[,colnames(OTU.clean.relabund)%in%colnames(OTU.abund02p)]

tax.clean[row.names(tax.clean)%in%colnames(OTU.abund02p),]$Genus

tax.clean[row.names(tax.clean)%in%colnames(OTU.abund02p),]$Family

colnames(OTU.abund05p)

[1] "Otu00001" "Otu00002" "Otu00003" "Otu00004" "Otu00005"

[6] "Otu00006" "Otu00007" "Otu00008" "Otu00009" "Otu00010"

[11] "Otu00011" "Otu00012" "Otu00013" "Otu00014" "Otu00015"

[16] "Otu00016" "Otu00017" "Otu00018" "Otu00019" "Otu00020"

[21] "Otu00021" "Otu00022" "Otu00023" "Otu00024" "Otu00025"

[26] "Otu00026" "Otu00027" "Otu00028" "Otu00029" "Otu00030"

[31] "Otu00031" "Otu00032" "Otu00033" "Otu00034" "Otu00035"

[36] "Otu00036" "Otu00037" "Otu00038" "Otu00039" "Otu00040"

[41] "Otu00041" "Otu00042" "Otu00043" "Otu00044" "Otu00045"

[46] "Otu00046" "Otu00047" "Otu00048" "Otu00049" "Otu00050"

[51] "Otu00051" "Otu00052" "Otu00053" "Otu00054" "Otu00055"

[56] "Otu00056" "Otu00057" "Otu00058" "Otu00059" "Otu00060"

[61] "Otu00062" "Otu00063" "Otu00064" "Otu00065" "Otu00066"

[66] "Otu00067" "Otu00068" "Otu00069" "Otu00070" "Otu00071"

[71] "Otu00072" "Otu00076" "Otu00080" "Otu00084" "Otu00088"

[76] "Otu00090" "Otu00091" "Otu00092" "Otu00093" "Otu00094"

[81] "Otu00095" "Otu00100" "Otu00103" "Otu00106" "Otu00110"

[86] "Otu00116" "Otu00117" "Otu00119" "Otu00120" "Otu00123"

[91] "Otu00128" "Otu00129" "Otu00138" "Otu00141" "Otu00153"

[96] "Otu00184" "Otu00353" "Otu00380" "Otu00416" "Otu00486"

[101] "Otu00664"

colnames(OTU.abund04p)

[1] "Otu00001" "Otu00002" "Otu00003" "Otu00004" "Otu00005"

[6] "Otu00006" "Otu00007" "Otu00008" "Otu00009" "Otu00010"

[11] "Otu00011" "Otu00012" "Otu00013" "Otu00014" "Otu00015"

[16] "Otu00016" "Otu00017" "Otu00018" "Otu00019" "Otu00020"

[21] "Otu00021" "Otu00022" "Otu00023" "Otu00024" "Otu00025"

[26] "Otu00026" "Otu00027" "Otu00028" "Otu00029" "Otu00030"

[31] "Otu00031" "Otu00032" "Otu00033" "Otu00034" "Otu00035"

[36] "Otu00036" "Otu00037" "Otu00038" "Otu00039" "Otu00040"

[41] "Otu00041" "Otu00042" "Otu00043" "Otu00044" "Otu00045"

[46] "Otu00046" "Otu00047" "Otu00048" "Otu00049" "Otu00050"

[51] "Otu00051" "Otu00052" "Otu00053" "Otu00054" "Otu00055"

[56] "Otu00056" "Otu00057" "Otu00058" "Otu00059" "Otu00060"

[61] "Otu00061" "Otu00062" "Otu00063" "Otu00064" "Otu00065"

[66] "Otu00066" "Otu00067" "Otu00068" "Otu00069" "Otu00070"

[71] "Otu00071" "Otu00072" "Otu00073" "Otu00076" "Otu00078"

[76] "Otu00080" "Otu00084" "Otu00087" "Otu00088" "Otu00089"

[81] "Otu00090" "Otu00091" "Otu00092" "Otu00093" "Otu00094"

[86] "Otu00095" "Otu00096" "Otu00098" "Otu00099" "Otu00100"

[91] "Otu00101" "Otu00103" "Otu00104" "Otu00105" "Otu00106"

[96] "Otu00109" "Otu00110" "Otu00112" "Otu00114" "Otu00115"

[101] "Otu00116" "Otu00117" "Otu00119" "Otu00120" "Otu00121"

[106] "Otu00123" "Otu00125" "Otu00127" "Otu00128" "Otu00129"

[111] "Otu00134" "Otu00138" "Otu00139" "Otu00140" "Otu00141"

[116] "Otu00144" "Otu00146" "Otu00147" "Otu00153" "Otu00161"

[121] "Otu00177" "Otu00184" "Otu00189" "Otu00234" "Otu00353"

[126] "Otu00380" "Otu00393" "Otu00416" "Otu00428" "Otu00441"

[131] "Otu00486" "Otu00664" "Otu00960"

colnames(OTU.abund1p)

[1] "Otu00001" "Otu00002" "Otu00003" "Otu00004" "Otu00005"

[6] "Otu00006" "Otu00007" "Otu00008" "Otu00009" "Otu00010"

[11] "Otu00011" "Otu00012" "Otu00013" "Otu00014" "Otu00015"

[16] "Otu00016" "Otu00017" "Otu00018" "Otu00019" "Otu00020"

[21] "Otu00021" "Otu00022" "Otu00023" "Otu00026" "Otu00027"

[26] "Otu00029" "Otu00030" "Otu00031" "Otu00032" "Otu00034"

[31] "Otu00035" "Otu00036" "Otu00037" "Otu00038" "Otu00040"

[36] "Otu00041" "Otu00042" "Otu00043" "Otu00045" "Otu00046"

[41] "Otu00055" "Otu00056" "Otu00063" "Otu00064" "Otu00092"

[46] "Otu00094" "Otu00095" "Otu00106" "Otu00123" "Otu00380"

genus.abund = t(tax.genus.count)[, apply(genus.relabund, MARGIN=2, function(x) any(x > 0.1))]

colnames(genus.abund01)

[1] "g__Prevotella" "g__Succiniclasticum"

[3] "unclassified" "g__Coprococcus"

[5] "g__Butyrivibrio" "g__Treponema"

[7] "g__CF231" "g__YRC22"

[9] "g__Pseudobutyrivibrio" "g__Ruminococcus"

[11] "g__Lachnospira" "g__Anaerovibrio"

[13] "g__Clostridium" "g__Selenomonas"

[15] "g__Fibrobacter" "g__Schwartzia"

[17] "g__Desulfovibrio" "g__BF311"

[19] "g__Shuttleworthia" "g__Ruminobacter"

[21] "g__Moryella" "g__Succinivibrio"

[23] "g__p.75.a5" "g__Mogibacterium"

[25] "g__Anaeroplasma" "g__.Prevotella."

[27] "g__Oscillospira" "g__RFN20"

[29] "g__Anaerostipes" "g__Bulleidia"

[31] "g__Dialister" "g__Pyramidobacter"

[33] "g__SHD.231" "g__Asteroleplasma"

colnames(genus.abund1)

[1] "g__Prevotella" "g__Succiniclasticum"

[3] "unclassified" "g__Coprococcus"

[5] "g__Butyrivibrio" "g__Treponema"

[7] "g__CF231" "g__YRC22"

[9] "g__Pseudobutyrivibrio" "g__Ruminococcus"

[11] "g__Clostridium" "g__Shuttleworthia"

#GENUS RELABUND 0.1%

genus.relabund.L<-t(tax.genus.count.L)/rowSums(t(tax.genus.count.L))*100

genus.abund.L01 = t(tax.genus.count.L)[, apply(genus.relabund.L, MARGIN=2, function(x) any(x > 0.1))]

corr.L<-bioenv(comm=genus.abund.L01, env=SCFA.L, method="kendall", index="bray")

genus.relabund.S<-t(tax.genus.count.S)/rowSums(t(tax.genus.count.S))*100

genus.abund.S01 = t(tax.genus.count.S)[, apply(genus.relabund.S, MARGIN=2, function(x) any(x > 0.1))]

corr.S<-bioenv(comm=genus.abund.S01, env=s, method="kendall", index="bray")

#FAMILY RELABUND 0.1%

family.relabund.L<-t(tax.family.count.L)/rowSums(t(tax.family.count.L))*100

family.abund.L01 = t(tax.family.count.L)[, apply(family.relabund.L, MARGIN=2, function(x) any(x > 0.1))] # 0.1 rel abund of family

cor.family.L01<-bioenv(comm=family.abund.L01, env=SCFA.L[,-(1:7)], method="kendall", index="bray")

family.relabund.S<-t(tax.family.count.S)/rowSums(t(tax.family.count.S))*100

family.abund.S01 = t(tax.family.count.S)[, apply(family.relabund.S, MARGIN=2, function(x) any(x > 0.1))]

cor.family.S01<-bioenv(comm=family.abund.S01, env=s[,-(1:7)], method="kendall", index="bray")

```

BETA DIVERSITY BY ENVIRONMENTAL VARIABLES AND SCFA

```{r}

BC.nmds = metaMDS(OTU.clean, distance="bray", k=2, trymax=1000)

BC.nmds.L = metaMDS(OTU.clean.L, distance="bray", k=2, trymax=1000)

BC.nmds.S= metaMDS(OTU.clean.S, distance="bray", k=2, trymax=1000)

BC.nmds.3D.L = metaMDS(OTU.clean.L, distance="bray", k=3, trymax=1000)

BCxyz.L = scores(BC.nmds.3D.L, display="sites")

BC.nmds.3D.S = metaMDS(OTU.clean.S, distance="bray", k=3, trymax=1000)

BCxyz.S = scores(BC.nmds.3D.S, display="sites")

P1<-plot_ly(x=BCxyz.L[,1], y=BCxyz.L[,2], z=BCxyz.L[,3], type="scatter3d", mode="markers", color=SCFA$Propionate.1, colors=c("blue", "green", "red"))

P2<-plot_ly(x=BCxyz.S[,1], y=BCxyz.S[,2], z=BCxyz.S[,3], type="scatter3d", mode="markers", color=s$Propionate__1, colors=c("blue", "green", "red"))

multiplot(P1, P2, cols=2)

```

COR SCATTER PLOT

```{r}

# Load data

#data("mtcars")

#df <- mtcars

#df$cyl <- as.factor(df$cyl)

df1<-cbind(genus.relabund.S01,SCFA.S[,-(4:10)])

df2<-cbind(genus.relabund.L01,SCFA.L)

# Scatter plot with correlation coefficient

#:::::::::::::::::::::::::::::::::::::::::::::::::

sp1 <- ggscatter(df1, x = "Propionate" , y = "Bulleidia",

add = "reg.line", # Add regressin line

add.params = list(color = "blue", fill = "lightgray"), # Customize reg. line

conf.int = TRUE # Add confidence interval

)

# Add correlation coefficient

sp1 + stat_cor(method = "pearson", label.x = 15, label.y = 0.5, label.x.npc = "center")

sp2 <- ggscatter(df2, x = "Propionate" , y = "Bulleidia",

add = "reg.line", # Add regressin line

add.params = list(color = "blue", fill = "lightgray"), # Customize reg. line

conf.int = TRUE # Add confidence interval

)

# Add correlation coefficient

sp2 + stat_cor(method = "pearson", label.x = 15, label.y = 0.2, label.x.npc = "center")

#sHUTTLEW.

sp3 <- ggscatter(df1, x = "Propionate" , y = "Shuttleworthia",

add = "reg.line", # Add regressin line

add.params = list(color = "blue", fill = "lightgray"), # Customize reg. line

conf.int = TRUE # Add confidence interval

)

# Add correlation coefficient

sp3 + stat_cor(method = "pearson", label.x = 15, label.y = 0.5, label.x.npc = "center")

sp4 <- ggscatter(df2, x = "Propionate" , y = "Shuttleworthia",

add = "reg.line", # Add regressin line

add.params = list(color = "blue", fill = "lightgray"), # Customize reg. line

conf.int = TRUE # Add confidence interval

)

# Add correlation coefficient

sp4 + stat_cor(method = "pearson", label.x = 15, label.y = 0.2, label.x.npc = "center")

#CF231

sp5 <- ggscatter(df1, x = "Propionate" , y = "CF231",

add = "reg.line", # Add regressin line

add.params = list(color = "blue", fill = "lightgray"), # Customize reg. line

conf.int = TRUE # Add confidence interval

)

# Add correlation coefficient

sp5 + stat_cor(method = "pearson", label.x = 15, label.y = 0.5, label.x.npc = "center")

sp6 <- ggscatter(df2, x = "Propionate" , y = "CF231",

add = "reg.line", # Add regressin line

add.params = list(color = "blue", fill = "lightgray"), # Customize reg. line

conf.int = TRUE # Add confidence interval

)

# Add correlation coefficient

sp6 + stat_cor(method = "pearson", label.x = 15, label.y = 0.2, label.x.npc = "center")

#Clostridium

sp7 <- ggscatter(df1, x = "Propionate" , y = "Clostridium",

add = "reg.line", # Add regressin line

add.params = list(color = "blue", fill = "lightgray"), # Customize reg. line

conf.int = TRUE # Add confidence interval

)

# Add correlation coefficient

sp7 + stat_cor(method = "pearson", label.x = 15, label.y = 2, label.x.npc = "center")

sp8 <- ggscatter(df2, x = "Propionate" , y = "Clostridium",

add = "reg.line", # Add regressin line

add.params = list(color = "blue", fill = "lightgray"), # Customize reg. line

conf.int = TRUE # Add confidence interval

)

# Add correlation coefficient

sp8 + stat_cor(method = "pearson", label.x = 15, label.y = 1, label.x.npc = "center")

#Pseudobutyrivibrio

sp11 <- ggscatter(df1, x = "Propionate" , y = "Pseudobutyrivibrio",

add = "reg.line", # Add regressin line

add.params = list(color = "blue", fill = "lightgray"), # Customize reg. line

conf.int = TRUE # Add confidence interval

)

# Add correlation coefficient

sp11 + stat_cor(method = "pearson", label.x = 15, label.y = 2, label.x.npc = "center")

sp12 <- ggscatter(df2, x = "Propionate" , y = "Pseudobutyrivibrio",

add = "reg.line", # Add regressin line

add.params = list(color = "blue", fill = "lightgray"), # Customize reg. line

conf.int = TRUE # Add confidence interval

)

# Add correlation coefficient

sp12 + stat_cor(method = "pearson", label.x = 15, label.y = 1, label.x.npc = "center")

#Coprococcus

sp9 <- ggscatter(df1, x = "Propionate" , y = "Coprococcus",

add = "reg.line", # Add regressin line

add.params = list(color = "blue", fill = "lightgray"), # Customize reg. line

conf.int = TRUE # Add confidence interval

)

# Add correlation coefficient

sp9 + stat_cor(method = "pearson", label.x = 15, label.y = 2, label.x.npc = "center")

sp10 <- ggscatter(df2, x = "Propionate" , y = "Coprococcus",

add = "reg.line", # Add regressin line

add.params = list(color = "blue", fill = "lightgray"), # Customize reg. line

conf.int = TRUE # Add confidence interval

)

# Add correlation coefficient

sp10 + stat_cor(method = "pearson", label.x = 15, label.y = 1, label.x.npc = "center")

#YRC22

sp13 <- ggscatter(df1, x = "Propionate" , y = "YRC22",

add = "reg.line", # Add regressin line

add.params = list(color = "blue", fill = "lightgray"), # Customize reg. line

conf.int = TRUE # Add confidence interval

)

# Add correlation coefficient

sp13 + stat_cor(method = "pearson", label.x = 15, label.y = 2, label.x.npc = "center")

sp14 <- ggscatter(df2, x = "Propionate" , y = "YRC22",

add = "reg.line", # Add regressin line

add.params = list(color = "blue", fill = "lightgray"), # Customize reg. line

conf.int = TRUE # Add confidence interval

)

# Add correlation coefficient

sp14 + stat_cor(method = "pearson", label.x = 15, label.y = 1, label.x.npc = "center")

```

PLOT-PROPIONATE (+) LIQUID & SOLID

BULLEIDIA

```{r}

sp7 <- ggscatter(df1, x = "Propionate" , y = "Bulleidia",

add = "reg.line", # Add regressin line

add.params = list(color = "blue", fill = "lightgray"), # Customize reg. line

conf.int = TRUE # Add confidence interval

)

# Add correlation coefficient

sp7s<-sp7 + stat_cor(method = "pearson", label.x = 16.2, label.y = 0.5, label.x.npc = "center", label.y.npc = "center")

sp8 <- ggscatter(df2, x = "Propionate" , y = "Bulleidia",

add = "reg.line", # Add regressin line

add.params = list(color = "blue", fill = "lightgray"), # Customize reg. line

conf.int = TRUE # Add confidence interval

)

# Add correlation coefficient

sp8s<-sp8 + stat_cor(method = "pearson", label.x = 16.2, label.y = 0.1, label.x.npc = "center", label.y.npc = "center")

```

COPROCOCCUS

```{r}

#Coprococcus

sp9 <- ggscatter(df1, x = "Propionate" , y = "Coprococcus",

add = "reg.line", # Add regressin line

add.params = list(color = "blue", fill = "lightgray"), # Customize reg. line

conf.int = TRUE # Add confidence interval

)

# Add correlation coefficient

sp9s<-sp9 + stat_cor(method = "pearson", label.x = 16.2, label.y = 2, label.x.npc = "center", label.y.npc = "center")

sp10 <- ggscatter(df2, x = "Propionate" , y = "Coprococcus",

add = "reg.line", # Add regressin line

add.params = list(color = "blue", fill = "lightgray"), # Customize reg. line

conf.int = TRUE # Add confidence interval

)

# Add correlation coefficient

sp10s<-sp10 + stat_cor(method = "pearson", label.x = 16.2, label.y = 1, label.x.npc = "center", label.y.npc = "center")

```

SHUTTLEWORTHIA

```{r}

sp3 <- ggscatter(df1, x = "Propionate" , y = "Shuttleworthia",

add = "reg.line", # Add regressin line

add.params = list(color = "blue", fill = "lightgray"), # Customize reg. line

conf.int = TRUE # Add confidence interval

)

# Add correlation coefficient

sp3s<-sp3 + stat_cor(method = "pearson", label.x = 16.2, label.y = 0.5, label.x.npc = "center", label.y.npc = "center")

sp4 <- ggscatter(df2, x = "Propionate" , y = "Shuttleworthia",

add = "reg.line", # Add regressin line

add.params = list(color = "blue", fill = "lightgray"), # Customize reg. line

conf.int = TRUE # Add confidence interval

)

# Add correlation coefficient

sp4s<-sp4 + stat_cor(method = "pearson", label.x = 16.2, label.y = 0.2, label.x.npc = "center", label.y.npc = "center")

```

TREPONEMA

```{r}

sp1 <- ggscatter(df2, x = "Propionate" , y = "Treponema",

add = "reg.line", # Add regressin line

add.params = list(color = "blue", fill = "lightgray"), # Customize reg. line

conf.int = TRUE # Add confidence interval

)

# Add correlation coefficient

sp1s<-sp1 + stat_cor(method = "pearson", label.x = 16.2, label.y = 0.5, label.x.npc = "center",label.y.npc = "center")

sp2 <- ggscatter(df1, x = "Propionate" , y = "Dialister",

add = "reg.line", # Add regressin line

add.params = list(color = "blue", fill = "lightgray"), # Customize reg. line

conf.int = TRUE # Add confidence interval

)

# Add correlation coefficient

sp2s<-sp2 + stat_cor(method = "pearson", label.x = 16.2, label.y = 0.2, label.x.npc = "center", label.y.npc = "center")

```

```{r}

multiplot(sp7s, sp9s, sp3s, sp1s,sp8s, sp10s, sp4s, sp2s, cols=2)

```
