# Supplementary material for: Characterization of rumen microbiota in lactating Holstein cows fed molasses versus corn grain at two levels of rumen-degradable protein
Source: Front Microbiomes. 2023 Aug 15;2:1204988. doi: 10.3389/frmbi.2023.1204988 (PMC12993626; doi:10.3389/frmbi.2023.1204988)
Supplement: Supplementary file 1 [file DataSheet_1.zip › Supplementary Figures and Tables.docx]

Supplementary Material

**Characterization of rumen microbiota in lactating Holstein cows fed molasses versus corn grain at different levels of rumen degradable protein.**

E. Guduk^1^, M. B. Hall^2^, G. I. Zanton^2^, A. J. Steinberger^3^, P. J. Weimer^3^, G. Suen^3^, K. A. Weigel^1^

^1^ Department of Dairy Science, University of Wisconsin-Madison, Madison, WI, USA

^2^ U. S. Dairy Forage Research Center, USDA-ARS, Madison, WI, USA

^3^ Department of Bacteriology, University of Wisconsin-Madison, Madison, WI, USA

*** Correspondence:**E. Guduk
[egunal@wisc.edu](mailto:egunal@wisc.edu)

**
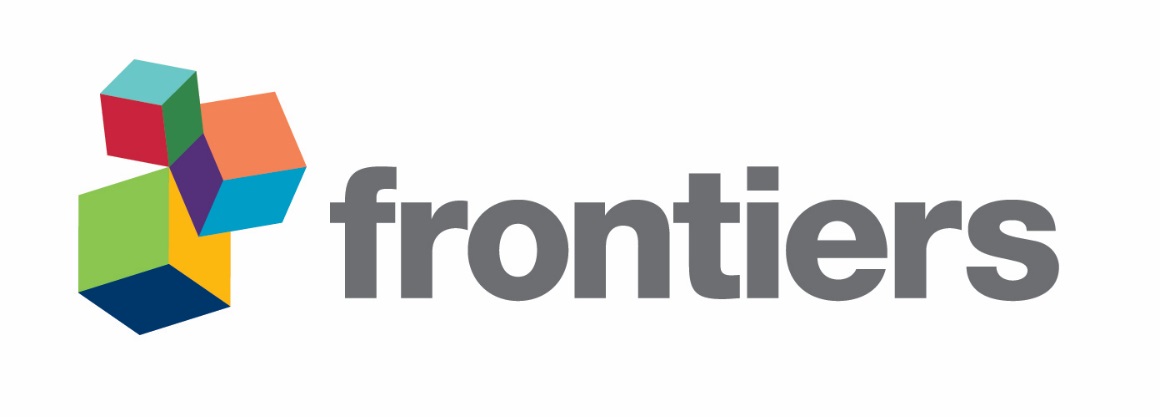
**

# Supplementary Data

**Captions respectively**:

## Data sheet 1. Code for Whole Analysis.docx

## Datasheet 2.xlsx

## Datasheet 3.Family level OTU counts.xlsx

## Datasheet 4. Liquid & Solid Together BCC Analysis.xlsx

## Datasheet 5. Ruminal Chemistry Data.xlsx

## COR.TABLE.S.pval.xlsx

## COR.TABLE.L.pval.xlsx

# Supplementary Figures and Tables

## Supplementary Figures

**Supplementary Figure 1.** Histogram of Alpha Statistics


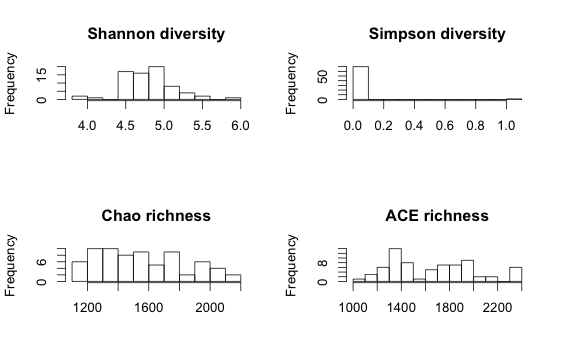


**Supplementary Figure 2**. Alpha Statistics with Transformed Simpson Diversity Metric


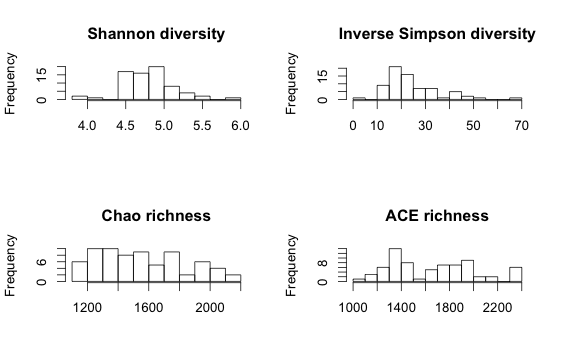


**Supplementary Figure 3A1.** Bar charts grouping liquid digesta samples by RDP levels only showing top 9 families within own their phyla.

**Supplementary Figure 3A2.** Bar charts grouping solid digesta samples by RDP levels only showing top 8 families within own their phyla.

**Supplementary Figure 3B1.** Bar charts grouping liquid digesta samples by molasses levels only showing top 9 families within own their phyla

**Supplementary Figure 3B2.** Bar charts grouping solid digesta samples by molasses levels only showing top 8 families within own their phyla.

## Supplementary Tables

| **Table S1.** P-values associated with PERMANOVA, ANOSIM, and ANOVA | | | | | | | | | | | | | | |  |
| --- | --- | --- | --- | --- | --- | --- | --- | --- | --- | --- | --- | --- | --- | --- | --- |
|  | Level | Index | Test | ST | RDP | M | Per | ST:Per | ST:Per:M | RDP:M | RDP:ST | M:ST | RDP:M:ST | M:Per |  |
| Digesta | OTU | Bray-Curtis | PERMANOVA | 0.001 | 0.021 | 0.552 | NA | NA | NA | 0.943 | 0.427 | 0.948 | 1.000 | NA | |
|  |  | Jaccard | PERMANOVA | 0.001 | 0.127 | 0.576 | NA | NA | NA | 0.961 | 0.408 | 0.944 | 1.000 | NA | |
|  | Diversity | Shannon | ANOVA | 2.27E-07 | 0.007 | 0.607 | 0.308 | 0.087 | 0.071 | 0.457 | 0.774 | 0.161 | NA | NA | |
|  | Richness | Chao | Kruskal-Wallis | 0.370 | 0.018 | 0.513 | 0.767 | NA | NA | NA | NA | NA | NA | NA | |
|  |  |  | Wilcoxon rank | 0.380 | 0.017 | NA | NA | NA | NA | NA | NA | NA | NA | NA | |
| Trees |  | Bray-Curtis | ANOSIM | 0.001 | 0.108 | 0.903 | 0.406 |  |  |  |  |  |  |  | |
|  |  | Jaccard | ANOSIM | 0.001 | 0.086 | 0.921 | 0.414 |  |  |  |  |  |  |  | |
| SCOA |  | Bray-Curtis | PERMANOVA | 0.976 | 0.153 | 0.446 | 0.019 | 1.000 | 1.000 | 0.978 | 0.976 | 0.997 | 0.996 | 0.021 | |
|  |  | Jaccard | PERMANOVA | 0.978 | 0.174 | 0.415 | 0.030 | 1.000 | 1.000 | 0.978 | 0.983 | 1.000 | 0.996 | 0.010 | |

ST: Sample type, Per: Period, M: molasses, RDP: rumen degradable protein.

| Table S2. Metadata of ruminal digesta from liquid- and Solid- fraction. Normalized to 18,286 sequences per sample^1^ | | | | | | | | |
| --- | --- | --- | --- | --- | --- | --- | --- | --- |
| Groups | Periods | Fractions | Total Sequences reads^1^ | Good's Coverage (%) | Ace | Chao | Shannon | Inverse-Simpson |
| L4251_P1 | 1 | Liquid | 135684 | 99.82 | 1610.5 | 1376.2 | 3.99 | 10.32 |
| L4251_P2 | 2 | Liquid | 64240 | 99.60 | 1959.4 | 1710.8 | 4.60 | 17.82 |
| L4251_P3 | 3 | Liquid | 90609 | 99.70 | 1383.6 | 1471.9 | 4.45 | 14.06 |
| L4628_P1 | 1 | Liquid | 98870 | 99.72 | 1833.5 | 1675.5 | 4.46 | 14.78 |
| L4628_P2 | 2 | Liquid | 58570 | 99.51 | 1449.6 | 1439.1 | 4.90 | 24.27 |
| L4628_P3 | 3 | Liquid | 53809 | 99.47 | 1326.3 | 1307.2 | 4.76 | 19.05 |
| L4668_P1 | 1 | Liquid | 109436 | 99.74 | 2192.4 | 1934.0 | 4.73 | 20.35 |
| L4668_P2 | 2 | Liquid | 66536 | 99.56 | 1995.4 | 1815.9 | 4.70 | 18.61 |
| L4668_P3 | 3 | Liquid | 41484 | 99.23 | 1396.0 | 1511.1 | 4.96 | 21.00 |
| L5000_P1 | 1 | Liquid | 95867 | 99.66 | 1966.9 | 1713.3 | 4.83 | 20.35 |
| L5000_P2 | 2 | Liquid | 71176 | 99.54 | 2342.9 | 2071.2 | 4.93 | 22.90 |
| L5000_P3 | 3 | Liquid | 55157 | 99.41 | 1793.2 | 1594.1 | 4.51 | 14.30 |
| L5017_P1 | 1 | Liquid | 97839 | 99.69 | 1825.4 | 1568.0 | 4.64 | 19.58 |
| L5017_P2 | 2 | Liquid | 50333 | 99.42 | 1450.3 | 1361.0 | 3.92 | 10.93 |
| L5017_P3 | 3 | Liquid | 48199 | 99.33 | 1301.0 | 1245.0 | 4.57 | 16.19 |
| L5019_P1 | 1 | Liquid | 84883 | 99.61 | 1248.4 | 1220.2 | 4.48 | 17.91 |
| L5019_P2 | 2 | Liquid | 33665 | 99.07 | 2383.7 | 1937.3 | 4.65 | 19.19 |
| L5019_P3 | 3 | Liquid | 48705 | 99.36 | 1348.7 | 1265.0 | 4.60 | 17.94 |
| L5020_P1 | 1 | Liquid | 64743 | 99.52 | 2355.3 | 1985.5 | 4.14 | 11.68 |
| L5020_P2 | 2 | Liquid | 57256 | 99.43 | 1510.9 | 1521.7 | 4.55 | 17.40 |
| L5020_P3 | 3 | Liquid | 54515 | 99.39 | 1699.6 | 1737.1 | 4.90 | 17.88 |
| L5046_P1 | 1 | Liquid | 79268 | 99.64 | 1348.3 | 1374.9 | 4.63 | 16.47 |
| L5046_P2 | 2 | Liquid | 60971 | 99.50 | 1307.5 | 1251.0 | 4.51 | 17.04 |
| L5046_P3 | 3 | Liquid | 38897 | 99.22 | 1292.3 | 1287.2 | 4.97 | 22.22 |
| L5047_P1 | 1 | Liquid | 75528 | 99.56 | 1334.9 | 1352.3 | 4.74 | 17.77 |
| L5047_P2 | 2 | Liquid | 63965 | 99.54 | 1985.6 | 1734.0 | 4.45 | 14.94 |
| L5047_P3 | 3 | Liquid | 62612 | 99.52 | 1821.8 | 1760.9 | 4.92 | 22.77 |
| L5048_P1 | 1 | Liquid | 106329 | 99.75 | 2105.2 | 1672.0 | 4.67 | 21.02 |
| L5048_P2 | 2 | Liquid | 60258 | 99.49 | 1777.5 | 1385.2 | 4.40 | 15.79 |
| L5048_P3 | 3 | Liquid | 44578 | 99.25 | 1410.8 | 1455.4 | 4.70 | 17.80 |
| L5054_P1 | 1 | Liquid | 72526 | 99.55 | 2307.7 | 1935.1 | 4.58 | 18.21 |
| L5054_P2 | 2 | Liquid | 96073 | 99.68 | 1494.8 | 1556.8 | 4.74 | 19.35 |
| L5054_P3 | 3 | Liquid | 49077 | 99.27 | 1618.5 | 1589.4 | 4.93 | 20.45 |
| L5067_P1 | 1 | Liquid | 73317 | 99.54 | 2350.6 | 1942.8 | 4.57 | 14.03 |
| L5067_P2 | 2 | Liquid | 86858 | 99.61 | 1692.5 | 1586.3 | 4.40 | 14.65 |
| L5067_P3 | 3 | Solid | 41715 | 99.15 | 1418.6 | 1423.0 | 4.90 | 21.09 |
| S4251_P1 | 1 | Solid | 65283 | 99.66 | 1732.3 | 1736.0 | 4.89 | 24.47 |
| S4251_P2 | 2 | Solid | 43555 | 99.47 | 1107.5 | 1125.3 | 4.72 | 22.22 |
| S4251_P3 | 3 | Solid | 28337 | 99.08 | 1816.0 | 2025.5 | 4.75 | 20.50 |
| S4628_P1 | 1 | Solid | 72473 | 99.70 | 1869.2 | 1652.1 | 5.16 | 34.40 |
| S4628_P2 | 2 | Solid | 44081 | 99.46 | 1099.7 | 1190.5 | 4.64 | 23.04 |
| S4628_P3 | 3 | Solid | 31181 | 99.34 | 1638.2 | 1438.1 | 4.41 | 17.38 |
| S4668_P1 | 1 | Solid | 60696 | 99.57 | 1429.4 | 1378.8 | 4.85 | 26.96 |
| S4668_P2 | 2 | Solid | 49865 | 99.44 | 1322.4 | 1263.9 | 4.94 | 30.26 |
| S4668_P3 | 3 | Solid | 37386 | 99.18 | 1941.0 | 1972.4 | 5.43 | 48.98 |
| S5000_P1 | 1 | Solid | 48529 | 99.51 | 1305.9 | 1244.1 | 4.94 | 29.28 |
| S5000_P2 | 2 | Solid | 42290 | 99.40 | 1351.8 | 1352.3 | 5.10 | 34.71 |
| S5000_P3 | 3 | Solid | 27379 | 98.93 | 2062.5 | 2133.0 | 4.88 | 27.15 |
| S5017_P1 | 1 | Solid | 42600 | 99.37 | 1194.2 | 1207.3 | 4.89 | 32.50 |
| S5017_P2 | 2 | Solid | 42939 | 99.35 | 1352.2 | 1382.0 | 5.19 | 48.61 |
| S5017_P3 | 3 | Solid | 24527 | 98.91 | 1232.6 | 1129.2 | 4.56 | 18.01 |
| S5019_P1 | 1 | Solid | 33615 | 99.27 | 1838.4 | 1569.5 | 4.87 | 31.11 |
| S5019_P2 | 2 | Solid | 37749 | 99.30 | 1272.2 | 1282.2 | 5.06 | 40.76 |
| S5019_P3 | 3 | Solid | 23168 | 98.76 | 1283.1 | 1162.7 | 4.78 | 27.55 |
| S5020_P1 | 1 | Solid | 19642 | 98.52 | 1359.8 | 1334.6 | 5.38 | 51.56 |
| S5020_P2 | 2 | Solid | 36363 | 99.23 | 1784.1 | 1789.9 | 5.31 | 44.43 |
| S5020_P3 | 3 | Solid | 31630 | 98.98 | 2330.9 | 1843.1 | 5.34 | 44.24 |
| S5046_P1 | 1 | Solid | 37140 | 99.38 | 1411.0 | 1404.8 | 4.44 | 16.44 |
| S5046_P2 | 2 | Solid | 34333 | 99.31 | 1712.3 | 1488.7 | 4.87 | 27.86 |
| S5046_P3 | 3 | Solid | 32097 | 99.16 | 1866.6 | 1586.9 | 4.54 | 17.33 |
| S5047_P1 | 1 | Solid | 33124 | 99.24 | 1992.2 | 1732.8 | 4.93 | 27.26 |
| S5047_P2 | 2 | Solid | 34147 | 99.19 | 2010.0 | 1741.8 | 5.07 | 28.15 |
| S5048_P1 | 1 | Solid | 28846 | 99.09 | 1996.5 | 2033.4 | 4.67 | 20.80 |
| S5048_P2 | 2 | Solid | 46108 | 99.42 | 1238.5 | 1194.8 | 4.84 | 27.96 |
| S5048_P3 | 3 | Solid | 24039 | 99.00 | 1198.9 | 1122.9 | 4.82 | 24.17 |
| S5054_P1 | 1 | Solid | 24556 | 98.78 | 1372.9 | 1263.4 | 5.05 | 31.96 |
| S5054_P2 | 2 | Solid | 55790 | 99.49 | 1970.6 | 1679.1 | 5.14 | 35.34 |
| S5054_P3 | 3 | Solid | 19239 | 98.43 | 1455.8 | 1423.8 | 5.54 | 66.59 |
| S5067_P1 | 1 | Solid | 29356 | 99.07 | 1728.1 | 2057.8 | 5.13 | 34.67 |
| S5067_P2 | 2 | Solid | 62126 | 99.59 | 1752.4 | 1680.6 | 5.23 | 40.53 |
| S5067_P3 | 3 | Solid | 26281 | 98.93 | 2044.8 | 2182.1 | 5.17 | 44.66 |

1 ACE and Chao, the richness index; Shannon and inverse-Simpson, the diversity index; 18,286, were selected based on the smallest sequence reads of the groups.

Table S3. FDR-adjusted P-values associated with PERMANOVA

|  |  |  | Index | |
| --- | --- | --- | --- | --- |
| Items^1^ | **Fractions** | **Test** | **Bray-Curtis** | **Jaccard** |
| RDP | Liquid | PERMANOVA | <0.01 | <0.01 |
|  | Solid | PERMANOVA | <0.01 | <0.01 |
| M | Liquid | PERMANOVA | 0.53 | 0.43 |
|  | Solid | PERMANOVA | 0.82 | 0.78 |
| RDP X M | Liquid | PERMANOVA | 0.43 | 0.45 |
|  | Solid | PERMANOVA | 0.58 | 0.7 |
| Fractions |  | PERMANOVA | <0.01 | <0.01 |

1 RDP=effects of rumen degradable protein; M= effects of dietary molasses levels; RDP X M=interaction RDP and M.

| **Table S4.**  Shapiro-Wilk normality test for Alpha diversity and richness metrics. | | |
| --- | --- | --- |
| Variable | W-statistics | p-value |
| Shannon diversity metrics | 0.974 | 0.1514 |
| Inverse Simpson diversity metrics | 0.888 | 1.15E-05 |
| Chao richness metrics | 0.957 | 0.0151 |
| Ace richness metrics | 0.938 | 0.0016 |
